# Developing a matrix to identify and prioritise research recommendations in HIV Prevention

**DOI:** 10.1186/1471-2458-11-381

**Published:** 2011-05-24

**Authors:** Sydney Anstee, Alison Price, Amanda Young, Katharine Barnard, Bob Coates, Simon Fraser, Rebecca Moran

**Affiliations:** 1 National Institute for Health Research (NIHR), Evaluation, Trials and Studies Coordinating Centre (NETSCC), Health Technology Assessment (HTA), University of Southampton, Southampton, SO16 7NS, UK; 2 National Institute for Health Research (NIHR), Evaluation, Trials and Studies Coordinating Centre (NETSCC), Public Health Programme, University of Southampton, Southampton, SO16 7NS, UK; 3 University of Southampton's Wessex Institute, Alpha House, University of Southampton Science Park, Southampton SO16 7NS, UK; 4 Public Health, Trust Headquarters, Southampton City PCT, Oakley Road, Southampton SO16 4GX, UK

## Abstract

**Background:**

HIV prevention continues to be problematic in the UK, as it does globally. The UK Department of Health has a strategic direction with greater focus on prevention as part of its World Class Commissioning Programme. There is a need for targeted evidence-based prevention initiatives. This is an exploratory study to develop an evidence mapping tool in the form of a matrix: this will be used to identify important gaps in contemporary HIV prevention evidence relevant to the UK. It has the potential to aid prioritisation in future research.

**Methods:**

Categories for prevention and risk groups were developed for HIV prevention in consultation with external experts. These were used as axes on a matrix tool to map evidence. Systematic searches for publications on HIV prevention were undertaken using electronic databases for primary and secondary research undertaken mainly in UK, USA, Canada, Australia and New Zealand, 2006-9. Each publication was screened for inclusion then coded. The risk groups and prevention areas in each paper were counted: several publications addressed multiple risk groups. The counts were exported to the matrix and clearly illustrate the concentrations and gaps of literature in HIV prevention.

**Results:**

716 systematic reviews, randomised control trials and other primary research met the inclusion criteria for HIV prevention. The matrix identified several under researched areas in HIV prevention.

**Conclusions:**

This is the first categorisation system for HIV prevention and the matrix is a novel tool for evidence mapping. Some important yet under-researched areas have been identified in HIV prevention evidence: identifying the undiagnosed population; international adaptation; education; intervention combinations; transgender; sex-workers; heterosexuals and older age groups.

Other research recommendations: develop the classification system further and investigate transferability of the matrix to other prevention areas; evidence syntheses may be appropriate in areas dense with research; have studies with positive findings been translated to practice?

The authors of this study invite research suggestions relating to the evidence gaps identified within remits of Public Health or any appropriate NETSCC programme.

Follow the 'Suggest Research' links from:

http://www.netscc.ac.uk/. Enter - **HIVProject **- in optional ID for HTA or in first information box for other programmes.

## Background

HIV/AIDS persists as a major global health priority with the number of people living with HIV continuing to increase[1]. A report from the Global HIV Prevention Working Group, a panel of leading AIDS experts, warned that prevention efforts are not keeping pace with the gains being made in treating people infected with HIV[2]. Revitalised global action is required for HIV prevention that supports a combination of behavioural, structural, and biomedical approaches[3].

In the UK 6,630 people were newly diagnosed as HIV-infected in 2009; half were diagnosed late in infection progression; approximately 22,000 are living with HIV but remain undiagnosed (from unlinked anonymous testing)[4]. Although new diagnoses appear to have declined slightly in recent years this reflects undiagnosed infection and diagnoses made abroad[5]. Undiagnosed infection presents two major problems: i) unaware onward transmission of HIV to others, ii) complications in presentation and treatment when late stage HIV is detected.

The scale and nature of sexual ill health and inequalities in England are of concern to policy makers[6] and consideration is currently being given to key action points in the national Sexual Health Strategy expected in 2011. The UK Department of Health has set the strategic direction for delivering healthcare with a greater focus on prevention as part of its World Class Commissioning Programme[7]. This includes recognition of the long term cost effectiveness of prevention strategies and the need to prioritise prevention to ensure viability of the NHS in the long term. There is potential for the methods outlined in this study to contribute to this aim through focused research directions.

Evidence mapping can make a positive contribution to policy agenda, with several research and policy gaps being fed into existing prioritisation channels: this was a concluding point from the Specialty Mapping Pilot by J. Shepherd in 2007[8]. It is one of the few published examples of innovative methodology to identify and prioritise topics for HTA and it concludes by recommending specialty mapping in other topic areas with on-going evaluation: these points were the catalyst for our study.

This study began on the premise that HIV prevention is an area in need of novel evidence-based interventions and that important yet under-researched areas within HIV prevention exist: these could be identified through thematic categorisation of the many possible combinations of risk groups and prevention related activities. We therefore devised a categorisation system for HIV prevention and piloted a matrix tool to identify gaps amongst the many contemporary studies on HIV prevention from countries with possible relation to HIV in the UK.

## Methods

A literature scoping exercise was undertaken to identify existing methodologies for conducting this work. The closest appropriate were the Global Evidence Mapping Initiative[9] and the Speciality Mapping Pilot [8]. These were discarded, however, because both were more suited to treatment care pathways than prevention. A new matrix system for mapping evidence was developed to collate publications into groups and combinations of prevention related activities, risk groups and study types. This was not an evidence synthesis and restricted resources did not allow for quality appraisal of the studies beyond mapping according to hierarchy of evidence.

## Development of the matrix

Four external experts were consulted and participated in the proposed classifications. These classifications represent important structural, contextual, behavioural, biomedical, service approaches, risk groups and combinations of these (see Additional file 1). There are important areas of research such as epidemiology that require investigation *before *interventions can be designed. A pilot was conducted on 100 publications to assess the robustness of the matrix and adjustments made accordingly.

### Publication searches

A search was made of the NIHR HTA programme Access databases (PROMIS) using the key term 'HIV' from inception in 1995 to March 2009. All topics identified and screened for being HIV prevention specific were eligible for inclusion. This longer time period was searched to provide additional internal audit, but research prior to 2006 was later excluded to match the time period of this study. A systematic search of major electronic databases including MEDLINE, MEDLINE In-Process, EMBASE, and Health Management Information Consortium (all Ovid) and the Cochrane Library were then searched using a carefully designed and tested search strategy (see Additional file 2). Primary research results were geographically filtered to studies undertaken in the UK, USA, Canada, Australia and New Zealand. Filtering by study location is liable to indexer error and bias. We were primarily interested in HIV prevention in the UK but USA publications were included as their vast research programme is a source of evidence for many other western countries. Canada, Australia, and New Zealand were included as other English speaking, developed countries with similar HIV epidemic and health service issues. To reduce publication bias we also searched a grey literature database for NHS reports.

Published studies were searched from 2006 to July 2009 as HIV is a rapidly changing area of health and contemporary evidence was deemed more useful in this exploration of the matrix. Due to the time and resource constraints the searches were limited to English language only, which is considered an acceptable limitation under such circumstances[10,11].

### Inclusion/Exclusion criteria

#### Inclusions

Any study where the stated aim is to investigate HIV prevention through epidemiology, policy, methods or various interventions; studies which aim to reduce infectivity of HIV positive people by reducing viral load (VL) through treatment adherence and reducing treatment resistance; studies which investigate behavioural and social factors stated to relate to risk of HIV infection; English speaking countries only; studies from UK, USA, Canada, Australia and New Zealand; publication dates 2006-2009 inclusive; systematic reviews, randomised controlled trials and other primary research of high quality (hierarchy of evidence) - case-controls, surveys, cohorts.

#### Exclusions

Studies which explore merits of HIV treatments but are not stated to be concerned with reducing viral load (VL)/infectivity to others e.g. side effects; depression/stress studies that do not mention links to risk of transmission or reduction of VL; studies that relate to HIV but are not stated to be concerned with risk of transmission e.g. relationships with children; primary research of low quality (hierarchy of evidence) - case studies, expert opinion, anecdotal evidence, narrative reviews.

#### Search results, coding and quality assurance

Search results were downloaded to three electronic Reference Manager (version 11) databases according to study types: systematic reviews; randomised controlled trials; other primary research (prospective/retrospective cohort studies, surveys, case-controls, but not case series). Each publication was screened for study type, date of publication, geographical area and if actually HIV prevention (see Inc/Exc criteria above). Included studies were screened against definitions (Additional file 1) and coded/counted accordingly. Many publications focused on more than one risk group, therefore achieved more counts e.g. A behavioural intervention for young, socially excluded drug-users counted 1 in each of the risk group cells under behaviour heading on the matrix = 3 counts. For verification purposes, search findings were checked against publications identified by Mimas Zetoc Alert Service for HIV prevention articles. The project lead was responsible for all coding of records, thereby not requiring inter-observer variation checks, however, quality assurance was conducted by a second team member taking random samples from each database to validate against our criteria.

#### Population of the matrix

Coded records were exported from Reference Manager to the matrix in an Excel spreadsheet which contained formulas for placing and counting the codes in corresponding cells within the matrix. Each cell was subdivided by study type, each column and row was totalled and each area with no research was left blank for easy visibility.

## Results

### Publication search results, coding and quality assurance

From 1995 to March 2009 NIHR, HTA received 80 suggestions and proposals relating to HIV prevention. Out of the 80 suggestions five were prioritised and research subsequently funded. Publications were evident for two of the five studies although one of these was excluded due to being outside the inclusion date range 2006-9 (the other studies are still ongoing). The one remaining included study was a systematic review on post-exposure prophylaxis (PEP)[12].

From electronic database searches we identified 1648 publications across the three research design groups. 40 were excluded in the initial screening for duplications. Through Mimas Zetoc Alert Service for HIV prevention articles two additional articles were found and added to our databases. A total of 1610 international publications were suitable for further analysis (Table 1). These studies were rigorously screened, coded and counted against inclusion criteria. Many publications focused on more than one risk group and were counted again. A second team member conducted quality assurance on coding: random samples from each database (n = 206) were checked: n = 8 errors were found (3.9%): two coded incorrectly and six required an additional code to one already given. 716 studies have 1 risk group; 127 studies - 2 risk groups; 25 studies - 3 risk groups = Total counts 868 (see full bibliography of included studies in Additional File 3).

**Table 1:** A summary of numbers of excluded and included publications.

Designs	Total identified	Excluded	Included (of which UK)		Total matrix counts
**Systematic Reviews**	*538*	*402*	*136*	***(20 incl. 1 HTA)***	*150*

**RCTs**	*461*	*200*	*261*	***(8)***	*322*

**Other Primary research**	*611*	*292*	*319*	***(28)***	*396*

**Totals**	*1610*	*894*	*716*	***(56)***	*868*

Papers focusing on more than one risk group achieved multiple counts. Inclusions and coding were based on stringent criteria.

### Population of the matrix

**Figure 1:**
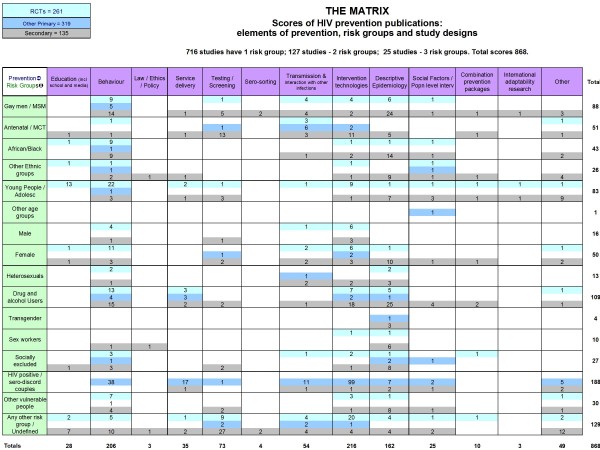
**The Matrix**. This is the tool devised to map HIV prevention evidence published 2006-9

After verification, the scores of the included studies were exported to the appropriate cells in the matrix. First impressions from the high numbers of studies identified suggest that HIV prevention is generally a heavily researched area, however, the results shown in the matrix draw attention to some unexpected exclusions within this field. The dense, sparse and empty areas of research are visually evident within HIV prevention activities, risk groups and study types - *See the matrix in Figure *1*and areas for prioritisation below.*

This method and tool could easily and rapidly be replicated in other prevention areas such as obesity by: a) defining headings for the matrix, b) search literature, c) code publications and export to matrix.

### Areas for prioritisation from the matrix findings

The matrix has highlighted several important areas within HIV prevention research that represent potential for novel and innovative research of interest to practitioners, commissioners, policy makers and researchers alike. We detail below those evidence gaps we believe would be most useful for future research in HIV prevention:

#### Sero-sorting: identifying the undiagnosed population

The Health Protection Agency (HPA) 2010 report shows over a quarter of people who are HIV+ve are not aware of their status[4]. Our analysis showed only four papers[13-16] investigating this area, all of which examine sero-status awareness and undiagnosed infection amongst gay men or undefined populations. A difficulty with sero-sorting is possible overlap with testing issues, which, for the purposes of this study, have a different focus - Additional file 1.

#### International adaptation

the majority of HIV prevention research originates from USA yet the matrix identified just three publications[17-19] on research to test possible cultural translation and adaptation for use in other countries. Two of these studies examined the difficulties of adaptation, but the third adapted a successful intervention from USA in London and has since been adopted UK wide. Gay men and young people were the only risk groups addressed here.

#### Law/ethics/policy

We searched for studies investigating the effects of interventions such as prosecutions and policies regarding confidentiality, mortgages, travel restrictions and ethics on HIV prevention. Three publications[20-22] were identified addressing sex workers, ethnic minorities and undefined risk groups. Admittedly, this is an area where study design may present some difficulties. All identified studies examined the impact of public policy initiatives.

#### Combination packages of interventions

Limited HIV prevention resources could be optimised through synergy. We identified ten publications addressing this area. The majority of these were systematic reviews that analysed more than one intervention. Only two[23,24] of these studies aimed to examine the value of combining levels of approaches.

#### Social/population level factors

Relative to other areas of prevention social factors are revealed to be researched in moderation; 25 studies were identified. These studies examine factors such as gender, race, housing, poverty; only two[25,26] of these investigate stigma and discrimination from the health provider perspective or the epidemic as a whole.

#### Education

*T*he matrix also revealed fewer than anticipated studies in this area of prevention. The matrix found 28 counts in this area, the majority of which focused on impact of traditional school education, leaflets and videos: small group or individual exposure. Surprisingly, there were only two publications[27,28] on the effectiveness of HIV mass-media campaigns.

#### Risk groups

Interestingly, the matrix has illustrated some least targeted but potentially emerging risk groups. These include older age groups, transgender, sex workers, male, heterosexuals, other ethnic (besides black/African), socially excluded and other vulnerable risk groups. Our findings indicate that risk groups such as HIV positive, drug and alcohol users, Men who have sex with men and young people are the most targeted groups for study sample populations. However, examining the quality of these studies went beyond the scope of the current research.

## Discussion

### Principle findings

To our knowledge this is the first study to identify research gaps in HIV prevention using this novel method. In order to develop the matrix, we created a classification of prevention related activities and risk groups; a novel building block of the study as called for by Akers in 2003[29]. The development of the matrix has provided a tool which we believe has potential transferability for scoping other disease prevention areas such as obesity or type II diabetes; which we hope will be investigated further. We have identified six key under-researched prevention areas (Table 2).

**Table 2:** Areas of HIV prevention research for prioritisation as identified by the matrix

Prevention Area	Research published 2006-9	Why Important?
*Sero-sorting: identifying and understanding the undiagnosed population*	4 secondary research	This population could have major implications as a source of new infections. We suggest research in the area of sero-sorting could help learn more about people within the undiagnosed population beyond the limits of current HPA unlinked anonymous surveillance, which may underestimate undiagnosed figures e.g. a recent community testing study in Scotland found 41.7% undiagnosed HIV[16]. Increasing testing facilities does not necessarily help someone recognise their need to test or overcome fears of testing.

*International adaptation research*	1 RCT 2 secondary research	More research here would be in the interests of shared learning and reduced duplication across many countries, not just USA and UK. Only One of three studies identified in this area points out that an HIV prevention intervention found to be effective in US cities might not be generalisable to different times and settings[17]. This area of research has great potential for the UK and the international community.

*Law/ethics/policy*	3 secondary research	The small number of studies found in this area was unexpected since a government white paper in 1998 attempted to update the Offences Against the Person Act 1861 and several prosecutions were successful in early 2000's. All the factors under this heading have potential to affect stigma and discrimination, barriers to testing and reluctance to disclose HIV status and are therefore potentially valuable areas for investigation.

*Combination packages of interventions*	3 RCTs 7 secondary research	If a person's reasons for being at risk are multi-faceted, interventions could be also. A recent article in the Lancet concludes: We now require an urgent and revitalised global movement for HIV prevention that supports a combination of behavioural, structural, and biomedical approaches and is based on scientifically derived evidence[3]. Potential to maximise impact through combining interventions was a reiterated message at the 2009 International Society for STD Research Conference; a sparsely researched area so far.

*Social/population level factors*	5 RCTs 5 other primary 11 secondary research	Recognition that social, economic, political, and environmental factors directly affect HIV risk and vulnerability has stimulated interest in structural approaches to HIV prevention[3,30]. Further investigation of factors such as stigma, discrimination and poverty could add valuable new knowledge and aid design of effective prevention strategies.

*Education*	18 RCTs 10 secondary research	Education can be employed in many different ways and settings, as a starting point for raising awareness, understanding transmission, how to protect against infection and reducing fear. It was sometimes difficult to separate education from behavioural interventions as one seeks to affect the other; we were guided by how studies described their interventions and what outcomes were used. More research in educational intervention could be valuable.

Akers[29] suggests that a nationally standardized HIV prevention intervention classification taxonomy would aid consistency and scientific validation across HIV intervention studies. A small number of external experts and our study team have developed a novel taxonomy; further development may improve this, but it is our opinion that we have contributed a promising start to providing a classification system.

### Limitations

There was no facility within the search strategy or Reference Manager to identify multiple publications from one study. A manual search of a quarter of the database found 11 publications from 5 studies. As there is no systematic way to identify multiplications or adjust the matrix cells with certainty we could extrapolate from this quarter sample and estimate that 44 papers (out of 717 included) will come from 20 studies. However, in our conclusions we concentrate on the least populated areas of the matrix (Table 2) and found no multiple publications amongst them.

Publications that focused on more than one risk group were problematic. By consensus the project team agreed to repeat the count according to the risk groups addressed. This appeared the only way to fairly represent groups studied; however, this can cause confusion when the counts of risk groups and prevention areas do not match the numbers of publications identified. This is an area for potential improvement.

The limited remit of this study did not enable us to critically appraise beyond the hierarchy of evidence. In heavily researched areas like behaviour change, the density of evidence does not necessarily equate to high quality, robust research or provide evidence that translates to effective prevention. However, in the USA the Centre for Disease Control have already set up a project to synthesise evidence in relation to behavioural interventions: The Prevention Synthesis Project[31].

## Conclusions

HIV prevention in the UK remains problematic; effectiveness of current prevention efforts appears limited; novel and innovative, evidence-based approaches are needed in HIV prevention. HIV prevention remains one of the great challenges for sexual health in the UK[32]. This study offers a method for identifying possible future directions in research. This, in turn, may lead to a more complete understanding of effective prevention approaches.

A matrix tool and classification system has been developed to identify important research gaps in HIV prevention; both may be useful in other health areas.

Several important gaps in English language HIV prevention research since 2006 were identified by the matrix: identifying and understanding the undiagnosed population; International adaptation of research; education; intervention combinations. The key under-researched risk groups were transgender, sex-workers, heterosexuals and older age groups.

Three additional future research recommendations have been identified during this study: investigate further use of the classification system we have developed and transferability of the matrix to other health prevention areas; evidence syntheses may be appropriate in areas dense with research, if not already being carried out by CDC Prevention Research Synthesis Project (Behaviour); have studies with positive findings have been translated to practice and how many recommending future research have been follow-up with funded studies?

The authors of this study invite research suggestions relating to the evidence gaps identified within remits of Public Health or any appropriate NETSCC programme.

Follow the 'Suggest' research links from:

http://www.netscc.ac.uk/. Enter - **HIVProject **- in optional ID for HTA or in first information box for other programmes.

## Abbreviations

NIHR: National Institute for Health Research; NETSCC: NIHR: Evaluation, Trials and Studies Coordinating Centre (NETSCC); HTA: Health Technology Assessment; HIV: Human Immunodeficiency virus; RCT: randomised controlled trial; HPA: Health Protection Agency.

## Competing interests

The authors declare that they have no competing interests.

## Authors' contributions

SA wrote the first draft of the paper and led the writing process. AP designed the search strategy and performed the publication searches. SA, BM and AJ analysed the data. BM contributed to quality assurance of data coding. All authors contributed to study design and provided input into the revisions of the paper. All authors were involved in the research design or analysis and approval of final draft submitted.

## Pre-publication history

The pre-publication history for this paper can be accessed here:

http://www.biomedcentral.com/1471-2458/11/381/prepub

## Supplementary Material

Additional file 1 **Definitions of risk groups and elements of prevention**. The file explains the working definitions for this study Click here for file

Additional file 2 **Search strategy**. The full search strategy conducted by Alison Price, information specialist Click here for file

Additional file 3 **Full bibliography of included studies**. The list and codes of all 716 studies found in searches. Click here for file
