# Supplementary material for: Developing a matrix to identify and prioritise research recommendations in HIV Prevention
Source: BMC Public Health. 2011 May 24;11:381. doi: 10.1186/1471-2458-11-381 (PMC3112419; doi:10.1186/1471-2458-11-381)
Supplement: Additional file 1 — Definitions of risk groups and elements of prevention. The file explains the working definitions for this study [file 1471-2458-11-381-S1.DOC]

**Appendix One**

**Developing a matrix to identify and prioritise research recommendations in HIV Prevention**

**TAXONOMY of PREVENTION and RISK GROUPS**

**PREVENTION**

**1. Education**

Specifically includes media campaigns and health promotion literature / social marketing (interventions to increase a

persons knowledge of HIV – increased awareness = increases informed choices)

Education is often ‘peer-led’ / school based or community – either through media, internet or in person. Education can

seek to affect attitudes, awareness and behaviour.

If research describes itself as ‘educational intervention’ – it is coded as this.

**2. Behaviour**

Behaviour is about things people do repeatedly. Behaviour change attempts to break these patterns - most often ‘risk

taking’ behaviour: peer influences/culture and other sexual behaviours like having multiple partners, using alcohol and

drugs, poor condom use, etc.

Research addressing behaviour may seek to understand it OR use various interventions to affect it.

Self-efficacy, disclosure of status, values, attitudes and self-esteem are examples of personal issues that affect behaviour.

**3. Law / Ethics / Policies - that affect HIV prevention**

Prosecutions, confidentiality, mortgages, travel

Research ethics approvals and general national/regional policies regarding HIV that may affect prevention – may inhibit

disclosure of HIV status or willingness to test for HIV

**4. Service delivery**

Access to services e.g. rural / community, clinic opening hours, etc. Quality of service given in the clinic – dedicated

clinics e.g. for YP, transgender, etc

GP v’s GUM clinic – quality of services for HIV, Quality of guides for GP’s

Combining GUM clinic with contraceptive services e.g. ease of cross-referral

Health service, social service and voluntary sector – organisation and delivery of health care

**5. Testing / Screening**

Testing for HIV; research may address ways to increase access to testing; increase community and opportunistic testing.

Different testing methods are being researched e.g. rapid finger prick testing, saliva testing

Accuracy of tests and ability to estimate time of infection

Screening programmes

Contact tracing / Partner notification

Counselling / mentoring / support before and after testing – called ‘pre and post test discussion’ – Esp. quality of care >

+ve diagnosis

**6. Sero-sorting the undiagnosed (establishing the HIV status of people who have not previously tested)**

Research into interventions to address the undiagnosed population and understand the barriers to testing – these are different to testing issues alone (as above)

Specific interventions such as the Unlinked Anonymous Testing by Health Protection Agency (HPA) seeks to estimate

what proportion of people living with HIV do not know they are positive, but surveillance is limited and may underestimated the numbers of the undiagnosed.

What other research is there in this area? Do most new infections come from the undiagnosed population or from people

who know they are HIV+ve?

**7. Transmission & interaction with other infections**

Understanding the natural science of HIV transmission – how it transmits under certain circumstances, what are the risks

in certain groups, how this knowledge can help inform the public to increase prevention e.g. oral sex, viral load (VL) in

different body fluids, etc

How other infections such as TB, herpes, gonorrhoea interact with HIV and how they affect transmission and increase

risks

How to reduce ‘infectivity and susceptibility’ – how infectious someone with HIV is to others and biological factors which

affect risk of acquisition of HIV

**8. Intervention technologies – tools and treatments**

Condom developments – materials, application styles, thickness, lubricants, etc

Needle-exchange, methadone and other treatments to reduce risk from injecting drug use

Vaccines ○ Microbicide gels ○ Male Circumcision ○ Post and Pre-exposure prophylaxis – PEP and Pr-EP

Any treatment studies that specifically say the interest is in reducing viral load – to reduce *infectivity*

Adherence to medication – even if the focus is not stated to be about reduction of VL, we assume this is an important

factor in adherence. It is the only aspect of medication we include as relevant to prevention

Complimentary and alternative medicines – only included if stated to reduce VL

**9. Descriptive epidemiology – ‘Know your epidemic!’**

Regional, sub-population data, trends over time, incidence/prevalence – studies seeking to understand epidemiology

before designing interventions

Personal level data – e.g. age, sexual activities, socio-economic groups, occupations, child-hood or depression predisposing

risk factors

Way and speed of clinic reports to Health Protection Agency (HPA)

How figures are reported to the public – and used in education / media, regional figures, etc

**10. Social Factors / Population level intervention – Pre-disposing risk factors**

Social elements such as environmental and economic factors that could affect prevention, but do not fit into any of the

other available categories e.g. wealth, community/social group, housing, education, social class, employment, etc

Stigma and discrimination is included here – often a population group / level problem

**11. Combination Prevention Packages**

Use and evaluation of several prevention intervention types in combination e.g. circumcision with a behavioural or

educational intervention with screening for other infections

**12. International Adaptability Research**

Research that tests the ability of successful interventions to be repeated / translated / adapted in another country e.g. a

prevention interventions has been shown to produce significant positive results in USA

– does it also produce significant positive results in the UK, if not how can it be adapted cultural to do as well as in USA?

**13. Other**

Anything else – methodology, cost analysis. (new groups are added here as they are found)

**RISK GROUPS**

**1. Gay men / MSM**

Studies that use either term gay or men-who-have-sex-with-men – any ethnicity – this can include men who do not

describe themselves as homosexual, because of culture, are bisexual, or because they only have sex with men

occasionally e.g. in prison, during a war, before marriage, etc

**2. Ante-natal / Mother-to-child transmission**

Risk of transmission from mother-to-child (also called vertical transmission) usually does not occur in utero, but during

birth, through breast-feeding and possibly risks into child-hood

Some studies look at the wellbeing of children of HIV+ve parents, but often are not about transmission – these are not

included as prevention studies

**3. Africans / Blacks**

Studies with focus on just this group but may use various terms African / black / black African – often African American –

include here. Also include African-Caribbean and all other African combinations

**4. Other ethnic groups - other than Black / African**

Any other ethnic groups e.g. Asian, Middle Eastern, Latino, etc

**5. Young people / Adolescent**

YP / teenagers and adolescent can be up to 18/19 or up to 24, but some studies describe their risk group as young adults

– e.g. young people may be up to 30. These ages groups included here

**6. Other age groups**

Older age groups (any above YP/adolescent above); may have specific issues e.g. divorcees unaccustomed to condoms,

swingers (often in 40’s), sex tourists

**7. Male – any nationality**

Studies specific to men only – gender issues, not sexuality

**8. Female – any nationality**

Studies specific to female - gender issues, not sexuality

**9. Heterosexuals**

Studies specific to heterosexuals – possibly emerging risk group outside of Africans e.g. sex tourists**,** divorcees

**10. Drug and alcohol users**

This could be addicts or recreational users (e.g. club nights). Includes injecting drug user – risk through needles - or any

other drug/alcohol use that affects risk behaviour e.g. increased risk of unprotected sex

**11. Transgender / transexual**

People who have either full or partial reversal of gender, or cross-dressing – transvestite – in preparation to change

gender

**12. Sex workers**

People of any sexual orientation or gender who sell sex – may often be studied in combination with drug use and other

vulnerabilities – but may also look at how well protected some groups of sex worker are too

**13. Socially excluded**

This group includes: people in prison, homeless, pregnant young women out of school, in-care, armed forces, etc

**14. HIV +ve / Sero-discordant couples**

Studies that look at risks of transmission of HIV from people who are already known to be HIV+ve

This group also includes people in couples where one person is HIV+ve and their partner is negative (sero-discordant)

**15. Other vulnerable people**

This includes other people with vulnerabilities such as: recently raped, psychological problems / mental health problems,

disabled, domestic abuse, gender-based violence, adults who experienced childhood sexual abuse

**16. Any other risk groups / Undefined**

Any other people who do not fit into previous named risk groups above – e.g. health care workers

This group is also for studies where particular risk groups are not specified e.g. anyone who comes into clinic, all people

completing on-line survey, etc
