# Supplementary material for: Developing a matrix to identify and prioritise research recommendations in HIV Prevention
Source: BMC Public Health. 2011 May 24;11:381. doi: 10.1186/1471-2458-11-381 (PMC3112419; doi:10.1186/1471-2458-11-381)
Supplement: Additional file 2 — Search strategy. The full search strategy conducted by Alison Price, information specialist [file 1471-2458-11-381-S2.DOC]

# Search Strategy : Interventions for HIV Prevention

**P = Population or Patient group:** HIV: at risk of, or positive already; search results to be sorted into **Risk Groups** on Grid.

**I = Intervention : Elements of prevention** as per grid (awareness, behaviour, testing, clinic/ GUM services, partner notification, treatment, law, statistics, barrier methods, prevention methods, safer sex, ethics and policies, stigma and discrimination etc)

**C = Comparator :** No intervention or existing best conventional intervention.

**O = Outcomes:** No search terms needed for outcomes

**T = Time period** Research since 2006

**Other Limits**

**Types of Research (following evidence hierarchy)**

- **Secondary research - Systematic reviews and meta analysis**
- **Primary research – RCTs**
- **Primary research - Cohort studies, case control studies, observational or prospective studies, case series.**

**Context**

- **UK research (initial search)**
- **USA, Canada, Australia, New Zealand (English as first language)**
- **Application of UK research to worldwide prevention (limit by UK primary author or institution) (secondary search)**

**Search dates**

- **From 2006 - July/August 2009**

**Language**

- **English**

**Sources**

**Published literature:**

Cochrane Database of Systematic Reviews

Cochrane Central Register of Controlled Trials

Medline (Ovid) database

Medline In process (Ovid)

Embase (Ovid)

HMIC (Ovid)

NIHR CRD databases: HTA, DARE, NHS EED

**Record sheet of HIV prevention searches**

INTERVENTION SEARCHES

| **Databases and years searched** | **Date searched and**  **search files** | **Number retrieved** | **Number of hits**  **(download file)** |
| --- | --- | --- | --- |
| Cochrane Library – CDSR 2009 Issue 3  Searched 21/08/09 | #1 (hiv) 7563  #2 human immunodeficiency virus 2744  #3 Acquired Immunodeficiency Syndrome 1622  #4 (#1 OR #2 OR #3) 8253  #5 (prevent* or reduc* or educat*) 202200  #6 (awareness or health promotion or safe sex or condom* or self efficacy or behav* or drug* or screen* or test* or contact* or notif* or counsel* or self-esteem or legal or confidential or anonymous or transmi* or viral load or adherence or vaccine* or gel* or circumcis* or phrophylaxis) 371971  #7 Health policy 5674  #8 life style or environment 6856  #9 (#5 OR #6 OR #7 OR #8) 428031  #10 (#4 AND #9) 7636  #11 (#10), from 2006 to 2009 | 276  (174 reviews;  102 protocols) | 69 reviews |
| Cochrane Library – CENTRAL 2009 Issue 3  Searched 21/08/09 | As above | 1341 | 352 |
| Ovid MEDLINE(R) <1996 to July Week 3 2009>  Searched 24/07/09 | 1 exp hiv infections/ (109538)  2 HIV.ti. (70462)  3 human immunodeficiency virus.ti,ab. (34413)  4 HIV Infections/pc (15094)  5 HIV/ or HIV-1/ (42561)  6 Acquired Immunodeficiency Syndrome/pc [Prevention & Control] (3911)  7 or/1-6 (135368)  8 limit 7 to (english language and yr="2006 -Current") (32801)  9 "Health Knowledge, Attitudes, Practice"/ (37355)  10 "Risk-Taking"/ (10170)  11 (awareness or education).ti. (29715)  12 Awareness/ (7013)  13 exp Health Promotion/ (25429)  14 exp Health Education/ (55500)  15 Contact Tracing/ (1724)  16 exp Preventive Health Services/ (176497)  17 exp Preventive Medicine/ (4747)  18 exp Primary Prevention/ (39507)  19 exp Behavior Therapy/ (18554)  20 exp Health Behavior/ (44901)  21 exp Sexual Behavior/ (29990)  22 exp risk reduction behavior/ or exp risk-taking/ or exp condoms/ (17344)  23 exp unsafe sex/ (1099)  24 exp safe sex/ (1400)  25 exp sexual abstinence/ (622)  26 exp Sex Education/ (2385)  27 Substance Abuse, Intravenous.mp. [mp=title, original title, abstract, name of substance word, subject heading word] (6500)  28 ((alcohol or drug*) adj3 risk).ti,ab. (5428)  29 ((prevent$ or reduc$ or educat$ or promot$ or increas$ or decreas$ or facilitat$ or barrier$ or encourag$) adj2 HIV).ti. (3069)  30 (screen* or test*).ti. (132754)  31 "Sensitivity and Specificity"/ (178889)  32 (finger adj5 test*).ti,ab. (562)  33 (saliva adj5 test*).ti,ab. (581)  34 contact trac*.ti,ab. (537)  35 (partner adj2 notif*).ti,ab. (431)  36 Point-of-Care Systems/ (3802)  37 self-efficacy.ti,ab. (5518)  38 ((counsel* or mentor*) adj5 test*).ti,ab. (2533)  39 (law or legal or prosecut*).ti. (11339)  40 self-esteem.ti,ab. (6129)  41 (stigma or discriminat*).ti,ab. (64008)  42 confidential*.ti. (1304)  43 anonymous test*.ti,ab. (141)  44 undiagnosed.ti,ab. (4881)  45 transmission.ti. (16777)  46 (viral load adj4 reduc*).ti. (55)  47 (adherence and viral load).ti,ab. (505)  48 (vaccine* or microbicide gel* or condom* or circumcision or prophylaxis).ti. (39145)  49 Ethics/ (3102)  50 exp ethics committees/ (3969)  51 Health Policy/ (22780)  52 (clinics or clinic or GUM).ti. (12744)  53 (access adj3 service*).ti. (259)  54 (combin* adj3 prevent*).ti,ab. (896)  55 exp sociology/ or social environment/ (424725)  56 exp life style/ (29110)  57 Population Surveillance/ (24440)  58 Disease Transmission, Infectious/ (4131)  59 or/9-57 (1069880)  60 8 and 59 (12402)  61 meta analysis.pt. (18971)  62 ((systematic$ or literature) adj2 (review$ or overview$)).ti,ab. (52547)  63 61 or 62 (66708)  64 60 and 63 (201)  65 randomized controlled trial.pt. (174971)  66 controlled clinical trial.pt. (33522)  67 random$.ti. (49676)  68 or/65-67 (219490)  69 60 and 68 (564)  70 69 not 64 (557)  71 exp cohort studies/ (454765)  72 exp case-control studies/ (324887)  73 exp longitudinal studies/ (398854)  74 ((observational or prospective) adj2 (study or studies)).ti,ab. (103346)  75 or/71-74 (734613)  76 60 and 75 (1622)  77 76 not 70 (1509)  78 exp great britain/ or exp england/ or London/ or scotland/ or wales/ (116320)  79 exp United States/ (436334)  80 exp Canada/ (47996)  81 exp South Australia/ or exp Australia/ or exp Western Australia/ or New Zealand (42830)  82 exp europe/ or andorra/ or austria/ or belgium/ or finland/ or exp france/ or exp germany/ or greece/ or iceland/ or ireland/ or italy/ or liechtenstein/ or luxembourg/ or exp mediterranean region/ or monaco/ or netherlands/ or portugal/ or san marino/ or exp scandinavia/ or spain/ or switzerland/ (421420)  83 or/78-82 (919259)  84 62 or 61 (66708)  85 60 and 84 (201)  86 83 and 70 (109)  87 83 and 77 (481)  88 70 not 86 (448)  89 from 64 keep 1-201 (201)  90 from 86 keep 1-109 (109)  91 from 87 keep 1-481 (481) | 201 SRs  557 RCTs  1509 Cohort studies | 200 SRs (English Language limit)  367 RCTs  (North America, Australasia, Western Europe, non-specific location)  481 cohort studies  (limited to (North America, Australasia, Western Europe indexed papers only) |
| EMBASE <1996 to 2009 Week 30>  Searched 30/07/09 | 1 exp human immunodeficiency virus 1/ or exp human immunodeficiency virus/ or exp human immunodeficiency virus 1 infection/ or exp human immunodeficiency virus 2/ or exp human immunodeficiency virus 2 infection/ or exp human immunodeficiency virus antibody/ or exp human immunodeficiency virus antigen/ (52946)  2 hiv.ti. (60431)  3 human immunodeficiency virus.ti,ab. (32395)  4 exp hiv infections/ (105194)  5 HIV/ or HIV-1/ (50435)  6 Acquired Immunodeficiency Syndrome/ (35719)  7 or/1-6 (132897)  8 limit 7 to (english language and yr="2006 -Current") (36008)  9 exp prevention/ or exp "prevention and control"/ or exp primary prevention/ (485629)  10 exp infection prevention/ or exp prophylaxis/ (268447)  11 "Health Knowledge, Attitudes, Practice"/ (2147)  12 "Risk-Taking"/ (2263)  13 (awareness or education).ti. (19770)  14 Awareness/ (14163)  15 exp Health Promotion/ (22156)  16 exp Health Education/ (64845)  17 Contact Tracing/ (427)  18 exp Preventive Health Services/ (4588)  19 exp Preventive Medicine/ (7594)  20 exp Primary Prevention/ (10059)  21 exp Behavior Therapy/ (16935)  22 exp Health Behavior/ (68797)  23 exp Sexual Behavior/ (38207)  24 exp risk reduction behavior/ or exp risk-taking/ or exp condoms/ (33083)  25 exp unsafe sex/ (318)  26 exp safe sex/ (1251)  27 exp sexual abstinence/ (38207)  28 exp Sex Education/ (1573)  29 Substance Abuse, Intravenous.mp. [mp=title, abstract, subject headings, heading word, drug trade name, original title, device manufacturer, drug manufacturer name] (0)  30 ((alcohol or drug*) adj3 risk).ti,ab. (5496)  31 ((prevent$ or reduc$ or educat$ or promot$ or increas$ or decreas$ or facilitat$ or barrier$ or encourag$) adj2 HIV).ti. (2338)  32 (screen* or test*).ti. (123810)  33 "Sensitivity and Specificity"/ (53892)  34 (finger adj5 test*).ti,ab. (521)  35 (saliva adj5 test*).ti,ab. (453)  36 contact trac*.ti,ab. (463)  37 (partner adj2 notif*).ti,ab. (358)  38 Point-of-Care Systems/ (1319)  39 self-efficacy.ti,ab. (4334)  40 ((counsel* or mentor*) adj5 test*).ti,ab. (2262)  41 (law or legal or prosecut*).ti. (8085)  42 self-esteem.ti,ab. (4903)  43 (stigma or discriminat*).ti,ab. (57468)  44 confidential*.ti. (782)  45 anonymous test*.ti,ab. (103)  46 undiagnosed.ti,ab. (4797)  47 transmission.ti. (14542)  48 (viral load adj4 reduc*).ti. (41)  49 (adherence and viral load).ti,ab. (481)  50 (vaccine* or microbicide gel* or condom* or circumcision or prophylaxis).ti. (36081)  51 Ethics/ (10773)  52 exp ethics committees/ (65015)  53 Health Policy/ (48839)  54 (clinics or clinic or GUM).ti. (11384)  55 (access adj3 service*).ti. (183)  56 (combin* adj3 prevent*).ti,ab. (881)  57 exp sociology/ or social environment/ (19124)  58 exp life style/ (27469)  59 Population Surveillance/ (63964)  60 Disease Transmission, Infectious/ (0)  61 or/9-60 (1028933)  62 8 and 61 (13775)  63 meta analysis/ (32162)  64 exp "systematic review"/ (27445)  65 ((systematic$ or literature) adj2 (review$ or overview$)).ti,ab. (49492)  66 63 or 64 or 65 (83260)  67 62 and 66 (325)  68 randomized controlled trial/ (141550)  69 controlled clinical trial/ (63736)  70 random$.ti. (49781)  71 or/68-70 (166678)  72 62 and 71 (549)  73 72 not 67 (517)  74 exp cohort studies/ (52896)  75 exp case-control studies/ (20355)  76 exp longitudinal studies/ (18014)  77 ((observational or prospective) adj2 (study or studies)).ti,ab. (101028)  78 or/74-77 (176720)  79 62 and 78 (813)  80 79 not 73 (758)  81 exp United Kingdom/ or exp england/ or London/ or scotland/ or wales/ (66396)  82 exp United States/ (123119)  83 exp Canada/ (28100)  84 exp australia/ or exp "australia and new zealand"/ (33935)  85 exp western europe/ or exp europe/ (268412)  86 or/81-85 (429760)  87 65 or 63 (76693)  88 62 and 87 (279)  89 86 and 73 (46)  90 86 and 80 (164)  91 73 not 89 (471) | 325 SRs  RCTs 471  Cohort studies  758 | 141 SRs  RCTs 130  Cohort studies  164  (limited to UK, North America, Australasia and Western Europe) |
| DARE (in Cochrane Library 2009/3)  Searched 21/08/09 | #1 (hiv) 7563  #2 human immunodeficiency virus 2744  #3 Acquired Immunodeficiency Syndrome 1622  #4 (#1 OR #2 OR #3) 8253  #5 (prevent* or reduc* or educat*) 202200  #6 (awareness or health promotion or safe sex or condom* or self efficacy or behav* or drug* or screen* or test* or contact* or notif* or counsel* or self-esteem or legal or confidential or anonymous or transmi* or viral load or adherence or vaccine* or gel* or circumcis* or phrophylaxis) 371971  #7 Health policy 5674  #8 life style or environment 6856  #9 (#5 OR #6 OR #7 OR #8) 428031  #10 (#4 AND #9) 7636  #11 (#10), from 2006 to 2009 | 99 | 54 |
| Cochrane Methodology Register | As above (Imported into Secondary Research database) | 29 | 16 |
| NHS EED (in Cochrane Library) | As above | 190 | 66 |
| HTA database (in Cochrane Library) | As above | 28 | 6 |
| HMIC Health Management Information Consortium < July 2009 >  Searched 24/08/09 | 1 HIV.ti. (2992)  2 human immunodeficiency virus.ti,ab. (751)  3 or/1-2 (4569)  4 (awareness or education).ti. (6728)  5 Awareness/ (278)  6 exp Health Promotion/ (6875)  7 exp Health Education/ (6001)  8 exp Preventive Medicine/ (16983)  9 exp risk reduction behavior/ or exp risk-taking/ or exp condoms/ (133)  10 exp Sex Education/ (565)  11 ((alcohol or drug*) adj3 risk).ti,ab. (553)  12 ((prevent$ or reduc$ or educat$ or promot$ or increas$ or decreas$ or facilitat$ or barrier$ or encourag$) adj2 HIV).ti. (338)  13 (screen* or test*).ti. (8176)  14 (finger adj5 test*).ti,ab. (20)  15 (saliva adj5 test*).ti,ab. (24)  16 contact trac*.ti,ab. (43)  17 (partner adj2 notif*).ti,ab. (41)  18 self-efficacy.ti,ab. (252)  19 ((counsel* or mentor*) adj5 test*).ti,ab. (225)  20 (law or legal or prosecut*).ti. (3397)  21 self-esteem.ti,ab. (589)  22 (stigma or discriminat*).ti,ab. (3063)  23 confidential*.ti. (667)  24 anonymous test*.ti,ab. (14)  25 undiagnosed.ti,ab. (154)  26 transmission.ti. (350)  27 (viral load adj4 reduc*).ti. (0)  28 (adherence and viral load).ti,ab. (8)  29 (vaccine* or microbicide gel* or condom* or circumcision or prophylaxis).ti. (1203)  30 Ethics/ (2403)  31 exp ethics committees/ (90)  32 Health Policy/ (3083)  33 (clinics or clinic or GUM).ti. (1497)  34 (access adj3 service*).ti. (301)  35 (combin* adj3 prevent*).ti,ab. (41)  36 exp sociology/ or social environment/ (1368)  37 exp life style/ (779)  38 or/4-37 (52149)  39 3 and 38 (294) | 294 | 96 |
| **ADAPTATION SEARCHES** |  |  |  |
| Ovid MEDLINE(R) <1996 to August Week 2 2009>  Searched 21/08/09 | 1 adaptation.ti,ab. (39759)  2 (culture and adapt*).mp. [mp=title, original title, abstract, name of substance word, subject heading word] (7513)  3 *"Adaptation, Psychological"/ (14372)  4 cultur*.ti,ab. (335618)  5 adapt*.ti,ab. (129937)  6 or/1-6 (468966)  7 (hiv adj5 (prevent* or intervention*)).ti,ab. (9792)  8 6 and 7 (992)  9 limit 13 to (english language and yr="2006 -Current") (388)  10 (meta analysis or review).pt. (940696)  11 9 and 10 (45)  12 ((cultural* or knowledge) adj5 (adaptation or transfer or disseminat*)).ti,ab. (1722)  13 12 and 7 (19)  14 "Cross-Cultural Comparison"/ (8986)  15 14 and 7 (23)  16 (international adj3 research).ti,ab. (2280)  17 16 and 7 (24)  18 9 or 11 or 13 or 15 or 17  19 limit 18 to yr="2006 -Current" | 64 | 64 |
| **EPIDEMIOLOGY SEARCHES** |  |  |  |
| Ovid MEDLINE(R) <1996 to August Week 2 2009>  Searched 24/08/09 | 1 HIV Infections/ep [Epidemiology] (14227)  2 descriptive epidemiology.ti,ab. (531)  3 1 and 2 (6)  4 *Incidence/ (136)  5 from 3 keep 1 (1)  6 *Prevalence/ (265)  7 incidence/ or prevalence/ (186157)  8 1 and 7 (4531)  9 review.pt. (928956)  10 meta analysis.pt. (19188)  11 9 or 10  12 11 and 8  13 randomized controlled trial.pt. (178003)  14 controlled clinical trial.pt. (33908)  15 random.ti. (4185)  16 13 or 15 or 14 (215194)  17 8 and 16 (50)  18 exp case-control studies/ or exp cohort studies/ or exp longitudinal studies/ or exp cross-sectional studies/ (781069)  19 18 and 8 (531) | 531 | 93 |
| EMBASE <1996 to 2009 Week 34>  Searched 24/08/09 | 1 HIV Infections/ep [Epidemiology] (14192)  2 descriptive epidemiology.ti,ab. (445)  3 1 and 2 (6)  4 *Incidence/ (347)  5 *Prevalence/ (861)  6 incidence/ or prevalence/ (224418)  7 1 and 6 (3514)  8 limit 7 to (english language and yr="2006 -Current") (1295)  9 review.pt. (735286)  10 9 and 8 (243)  11 meta analysis.pt. (0)  12 8 and 11 (0)  13 Human immunodeficiency virus infection/ep [Epidemiology] (14192)  14 (prevention and control).mp. [mp=title, abstract, subject headings, heading word, drug trade name, original title, device manufacturer, drug manufacturer name] (36694)  15 13 and 14 (836)  16 6 or 4 or 5 (224418)  17 16 and 15 (210)  18 10 or 17 (424) | 424 | 111 |
|  | TOTAL EPIDEMIOLOGY SEARCHES |  | Reviews 170  RCTs 5  Cohorts 62 |
| **RESEARCH IN PROGRESS** |  |  |  |
| CDSR Protocols |  | 102 | 32 |
| Current Controlled Trials | SEARCH TO BE DONE FOR OVERLAP WHEN GAPS IDENTIFIED |  |  |
| Clinical Trials.gov | SEARCH TO BE DONE FOR OVERLAP WHEN GAPS IDENTIFIED |  |  |
| **KEYWORDS IN REFMAN DATABASES** | SYSTEMATIC REVIEW SEARCH  Inc METHODS STUDIES  Inc ADAPTATION SEARCHES  Inc EPIDEMIOLOGY SEARCHES  Inc COST EFFECTIVENESS SEARCHES  RCTS SEARCH  Inc EPIDEMIOLOGY SEARCH  Inc ADAPTATION SEARCHES  COHORT STUDIES SEARCH  Inc HMIC  Inc EPIDEMIOLOGY SEARCHES  Inc ADAPTATION SEARCHES |  | **611**  8  64  170  61  **475**  5  4  **593**  91  86  6 |
| **RESULTS SRs & REVIEWS** | **TOTAL IN HIV SECONDARY RESEARCH DATABASE** |  | **611** |
| **RESULTS RCTs** | **TOTAL IN RCTS DATABASE** |  | **475** |
| **RESULTS COHORTS** | **TOTAL IN HIV COHORT STUDIES DATABASE** |  | **593** |
|  | **TOTAL IN HIV ONGOING TRIAL DATABASE** |  | **32** |
| **EXCLUDED AS NON-ENGLISH SPEAKING COUNTRIES** | **TOTAL IN NON-ENGLISH SPEAKING HIV PREVENTION**  **Inc SYSTEMATIC REVIEW SEARCH (16)**  **Inc RCTS SEARCH (48)**  **Inc COHORT STUDIES SEARCH (176)** |  | **248** |
| **TOTAL RESULTS** | **TOTAL No RECORDS in HIV EVIDENCE MAPPING** |  | **1679** |
