# Supplementary material for: Developing a matrix to identify and prioritise research recommendations in HIV Prevention
Source: BMC Public Health. 2011 May 24;11:381. doi: 10.1186/1471-2458-11-381 (PMC3112419; doi:10.1186/1471-2458-11-381)
Supplement: Additional file 3 — Full bibliography of included studies. The list and codes of all 716 studies found in searches. [file 1471-2458-11-381-S3.RTF]

Developing a matrix to identify and prioritise research recommendations in HIV Prevention – S.Anstee. NIHR Research on Research Programme. Aug 2010
Developing a matrix to identify and prioritise research recommendations in HIV Prevention 

Full Reference List

716 studies were screened against definitions and coded/counted accordingly.  Many publications focused on more than one risk group and were scored again e.g. A behavioural intervention for young, socially excluded, drug-users counted 1 in each of the risk groups under the behaviour heading in the matrix = 3 counts. 
716 studies had 1 risk group; 127 studies - 2 risk groups; 25 studies - 3 risk groups = Total codes / counts 868. 
Risk Groups: R01-Gay men / MSM; R02-Antenatal / MCT; R03-African/Black; R04-Other Ethnic groups; R05-Young People / Adolesc; R06-Other age groups; R07-Male; R08-Female; R09-Heterosexuals; R10-Drug and alcohol Users; R11-Transgender; R12-Sex workers; R13 Socially excluded; R14-HIV positive / sero-discord couples; R15-Other vulnerable people; R16- Any other risk group / Undefined.
Prevention Categories: P01-Education (incl school and media); P02-Behaviour; P03-Law / Ethics / Policy; P04-Service delivery; P05-Testing / Screening; P06-Sero-sorting; P07-	Transmission & interaction with other infections; P08-Intervention technologies; P09-	Descriptive Epidemiology; P10-Social Factors / Popn level interventions; P11-Combination prevention packages; P12-International adaptation research; P13-Other
For Codes see end of each reference e.g. R05xP01 – this converts to 1 score
Education (P01) 
1 code
Abbey A, Saenz C, Buck PO, Parkhill MR, Hayman LW. The effects of acute alcohol consumption, cognitive reserve, partner risk, and gender on sexual decision making. Journal of studies on alcohol 2006;67(1):113-21.R05XP01/1; 
Albarracin D, Durantini MR, Earl A, Gunnoe JB, Leeper J. Beyond the most willing audiences: a meta-intervention to increase exposure to HIV-prevention programs by vulnerable populations. Health Psychology 2008;27(5):638-44.R16XP01/3; 
Albarracin D, Leeper J, Earl A, Durantini MR. From brochures to videos to counseling: exposure to HIV-prevention programs. AIDS & Behavior 2008;12(3):354-62.R16XP01/3; 
Anderman EM, Lane DR, Zimmerman R, Cupp PK, Phebus V. Comparing the Efficacy of Permanent Classroom Teachers to Temporary Health Educators for Pregnancy and HIV Prevention Instruction. Health Promot Pract 2008.R05XP01/1; 
Bertrand JT, O'Reilly K, Denison J, Anhang R, Sweat M. Systematic review of the effectiveness of mass communication programs to change HIV/AIDS-related behaviours in developing countries. Health Education Research 2006;21(4):567-97.R16XP01/3; 
Borawski EA, Trapl ES, ms-Tufts K, Hayman LL, Goodwin MA, Lovegreen LD. Taking be proud! Be responsible! To the suburbs: a replication study. Perspectives on Sexual & Reproductive Health 2009;41(1):12-22.R05XP01/1; 
Coyle KK, Kirby DB, Robin LE, Banspach SW, Baumler E, Glassman JR. All4You! A randomized trial of an HIV, other STDs, and pregnancy prevention intervention for alternative school students. AIDS Education & Prevention 2006;18(3):187-203.R05XP01/1; 
Di NJ, Schinke SP. Gender-specific HIV prevention with urban early-adolescent girls: outcomes of the Keepin' It Safe Program. AIDS Education & Prevention 2007;19(6):479-88.R05XP01/1; R08XP01/1.
Evans WD, Blitstein J, Hersey JC, Renaud J, Yaroch AL. Systematic review of public health branding. Journal of Health Communication 13(8)()(pp 721-741), 2008 Date of Publication: December 2008 2008;(8):721-41.R16XP01/3; 
Gallegos EC, Villarruel AM, Gomez MV, Onofre DJ, Zhou Y. Research brief: sexual communication and knowledge among Mexican parents and their adolescent children. Journal of the Association of Nurses in AIDS Care 2007;18(2):28-34.R05XP01/1; 
Giles ML, Garland SM, Grover SR, Lewin SM, Hellard ME. Impact of an education campaign on management in pregnancy of women infected with a blood-borne virus. Medical Journal of Australia 2006;184(8):389-92.R02XP01/3; 
Griffin KW, Botvin GJ, Nichols TR. Effects of a school-based drug abuse prevention program for adolescents on HIV risk behavior in young adulthood. Prev Sci 2006;7(1):103-12.R05XP01/1; 
Keys TR, Morant KM, Stroman CA. Black youth's personal involvement in the HIV/AIDS issue: does the public service announcement still work? Journal of Health Communication 2009;14(2):189-202.R03XP01/1; R05XP01/1.
Malow RM, Stein JA, McMahon RC, Devieux JG, Rosenberg R, Jean-Gilles M. Effects of a culturally adapted HIV prevention intervention in Haitian youth. Journal of the Association of Nurses in AIDS Care 2009;20(2):110-21.R05XP01/1; 
Markham C, Tortolero S, Peskin M, Shegog R, Addy R, Baumler E et al. Short-term impact evaluation of 'It's Your Game, Keep It Real': A multimedia HIV/STI and pregnancy prevention intervention for middle school youth. American Public Health Association 134th Annual Meeting & Exposition; Nov 4 2006; Boston,MA 2006.R05XP01/1; 
Meekers D, Rahaim S. The importance of socio-economic context for social marketing models for improving reproductive health: evidence from 555 years of program experience. BMC Public Health 2005;5:10.R16XP01/3; 
Milhausen RR, DiClemente RJ, Lang DL, Spitalnick JS, Sales JM, Hardin JW. Frequency of sex after an intervention to decrease sexual risk-taking among African-American adolescent girls: Results of a randomized, controlled clinical trial. Sex Education 2008;8(1):47-57.R08XP01/3; 
Noar SM, Palmgreen P, Chabot M, Dobransky N, Zimmerman RS. A 10-year systematic review of HIV/AIDS mass communication campaigns: Have we made progress?. [Review] [97 refs]. Journal of Health Communication 2009;14(1):15-42.R16XP01/3; 
Richter DL, Dauner KN, Lindley LL, Reininger BM, Oglesby WH, Prince MS et al. Evaluation results of the CDC/ASPH Institute for HIV Prevention Leadership: a capacity-building educational program for HIV prevention program managers. Journal of Public Health Management & Practice 2007;Suppl:S64-S71.R16XP01/1 - HEALTH PROVIDERS; 
Tortolero SR, Markham CM, Addy RC, Baumler ER, Escobar-Chaves SL, Basen-Engquist KM et al. Safer choices 2: rationale, design issues, and baseline results in evaluating school-based health promotion for alternative school students. Contemporary Clinical Trials 2008;29(1):70-82.R05XP01/1; 
Vidanapathirana J, Abramson MJ, Forbes A, Fairley C. Mass media interventions for promoting HIV testing: Cochrane systematic review. [Review] [1 refs]. International Journal of Epidemiology 2006;35(2):233-4.R16XP01/3; R16XP05/3.
Villarruel AM, Jemmott III JB, Jemmott LS. A randomized controlled trial testing an HIV prevention intervention for Latino youth. Archives of Pediatrics and Adolescent Medicine 160(8)()(pp 772-777), 2006 Date of Publication: 2006 2006;(8):772-7.R05XP01/1; R04XP01/1.
Warner L, Klausner JD, Rietmeijer CA, Malotte CK, O'Donnell L, Margolis AD et al. Effect of a brief video intervention on incident infection among patients attending sexually transmitted disease clinics. PLoS medicine 2008;5(6):e135.R16XP01/1; 
Wolitski RJ. Relative efficacy of a multisession sexual risk-reduction intervention for young men released from prisons in 4 states. American Journal of Public Health 2006;96(10):1854-61.R13XP01/3; 
Zimmerman RS, Cupp PK, Donohew L, Sionean CK, Feist-Price S, Helme D. Effects of a school-based, theory-driven HIV and pregnancy prevention curriculum. Perspectives on Sexual & Reproductive Health 2008;40(1):42-51.R05XP01/1; 
		2 codes
Di NJ, Schinke SP. Gender-specific HIV prevention with urban early-adolescent girls: outcomes of the Keepin' It Safe Program. AIDS Education & Prevention 2007;19(6):479-88.R05XP01/1; R08XP01/1.
Keys TR, Morant KM, Stroman CA. Black youth's personal involvement in the HIV/AIDS issue: does the public service announcement still work? Journal of Health Communication 2009;14(2):189-202.R03XP01/1; R05XP01/1.
Villarruel AM, Jemmott III JB, Jemmott LS. A randomized controlled trial testing an HIV prevention intervention for Latino youth. Archives of Pediatrics and Adolescent Medicine 160(8)()(pp 772-777), 2006 Date of Publication: 2006 2006;(8):772-7.R05XP01/1; R04XP01/1.

Behaviour (P02)
1 code
Absalon J, Fuller CM, Ompad DC, Blaney S, Koblin B, Galea S et al. Gender differences in sexual behaviors, sexual partnerships, and HIV among drug users in New York City. AIDS & Behavior 2006;10(6):707-15.R10XP02/3; 
Aidala AA, Lee G, Garbers S, Chiasson MA. Sexual behaviors and sexual risk in a prospective cohort of HIV-positive men and women in New York City, 1994-2002: implications for prevention. AIDS Education & Prevention 2006;18(1):12-32.R14XP02/2; 
Arasteh K, Des J, Perlis TE. Alcohol and HIV sexual risk behaviors among injection drug users. Drug & Alcohol Dependence 2008;95(1-2):54-61.R10XP02/3; 
Bacon O, Lum P, Hahn J, Evans J, Davidson P, Moss A et al. Commercial sex work and risk of HIV infection among young drug-injecting men who have sex with men in San Francisco. Sexually Transmitted Diseases 2006;33(4):228-34.R01XP02/3; R12XP02/3.
Barry D, Weinstock J, Petry NM. Ethnic differences in HIV risk behaviors among methadone-maintained women receiving contingency management for cocaine use disorders. Drug & Alcohol Dependence 2008;98(1-2):144-53.R03XP02/1; R08XP02/1.
Beadnell B, Baker SA, Morrison DM, Huang B, Stielstra S, Stoner S. Change trajectories in women's STD/HIV risk behaviors following intervention. Prevention Science 2006;7(3):321-31.R08XP02/1; 
Begley K, Chan DJ, Jeganathan S, Batterham M, Smith DE. Correlates of unprotected anal intercourse in HIV positive men attending an HIV/AIDS clinic in Sydney. Current HIV Research 2008;6(6):579-84.R14XP02/2; 
Bell C, Richardson D, Wall M, Goldmeier D. HIV-associated female sexual dysfunction - Clinical experience and literature review. International Journal of STD and AIDS 2006;17(10):706-9.R14XP02/2; 
Berg C, Raminani S, Greer J, Harwood M, Safren S. Participants' perspectives on cognitive-behavioral therapy for adherence and depression in HIV. Psychotherapy Research 2008;18(3):271-80.R14XP02/2; 
Berkman A, Cerwonka E, Sohler N, Susser E. A randomized trial of a brief HIV risk reduction intervention for men with severe mental illness.[see comment]. Psychiatric Services 2006;57(3):407-9.R01XP02/3; R15XP02/3.
Berkman A, Pilowsky DJ, Zybert PA, Herman DB, Conover S, Lemelle S et al. HIV prevention with severely mentally ill men: a randomised controlled trial. AIDS Care 2007;19(5):579-88.R15XP02/3; 
Berkman A, Cerwonka E, Sohler N, Susser E. A randomised trial of a brief HIV risk reduction intervention for men with severe mental illness. Psychiatric Services 2006;57(3):407-9.R07XP02/3; R15XP02/3.
Bowen AM, Horvath K, Williams ML. A randomized control trial of Internet-delivered HIV prevention targeting rural MSM. Health Education Research 2007;22(1):120-7.R01XP02/3; 
Bowen AM, Williams ML, Daniel CM, Clayton S. Internet based HIV prevention research targeting rural MSM: feasibility, acceptability, and preliminary efficacy. Journal of Behavioral Medicine 2008;31(6):463-77.R01XP02/1; R16XP02/1.
Brown EJ, Van HM. Risk behavior, perceptions of HIV risk, and risk-reduction behavior among a small group of rural African American women who use drugs. Journal of the Association of Nurses in AIDS Care 2006;17(5):42-50.R03XP02/1; R08XP02/1.
Brown LK, Tolou-Shams M, Lescano C, Houck C, Zeidman J, Pugatch D et al. Depressive symptoms as a predictor of sexual risk among African American adolescents and young adults. Journal of Adolescent Health 2006;39(3):444-8.R14XP02/2; 
Bryan A, Ray LA, Cooper ML. Alcohol use and protective sexual behaviors among high-risk adolescents. Journal of Studies on Alcohol & Drugs 2007;68(3):327-35.R05XP02/3; R15XP02/3.
Bull S, Pratte K, Whitesell N, Rietmeijer C, McFarlane M. Effects of an Internet-based intervention for HIV prevention: the Youthnet trials. AIDS & Behavior 2009;13(3):474-87.R05XP02/1; 
Burdon WM, De L, Prendergast ML. The NOBLE study: assesing the effectiveness of residential vs intensive outpatient prison-based treatment and inmates views on HIV/AIDS-realted behaviours and services. Proceedings of the 70th Annual Scientific Meeting of the College on Problems of Drug Dependence; 2008 June 14 19; San Juan, Puerto Rico, USA 2008;26.R10XP02/3; R13XP02/3.
Carey MP, Vanable PA, Senn TE, Coury-Doniger P, Urban MA. Evaluating a two-step approach to sexual risk reduction in a publicly-funded STI clinic: rationale, design, and baseline data from the Health Improvement Project-Rochester (HIP-R). Contemporary Clinical Trials 2008;29(4):569-86.R16XP02/1; 
Carrico AW, Johnson MO, Moskowitz JT, Neilands TB, Morin SF, Charlebois ED et al. Affect regulation, stimulant use, and viral load among HIV-positive persons on anti-retroviral therapy. Psychosomatic Medicine 2007;69(8):785-92.R10XP02/2; R14XP07/2.
Carrico AW, Chesney MA, Johnson MO, Morin SF, Neilands TB, Remien RH et al. Randomized controlled trial of a cognitive-behavioral intervention for HIV-positive persons: an investigation of treatment effects on psychosocial adjustment. AIDS & Behavior 2009;13(3):555-63.R14XP02/2; 
Chawarski MC, Mazlan M, Schottenfeld RS. Behavioral drug and HIV risk reduction counseling (BDRC) with abstinence-contingent take-home buprenorphine: a pilot randomized clinical trial. Drug & Alcohol Dependence 2008;94(1-3):281-4.R10XP02/1; 
Comulada WS, Weiss RE, Cumberland W, Rotheram-Borus MJ. Reductions in drug use among young people living with HIV. American Journal of Drug & Alcohol Abuse 2007;33(3):493-501.R14XP02/2; 
Costenbader EC, Astone NM, Latkin CA. The dynamics of injection drug users' personal networks and HIV risk behaviors.[see comment]. Addiction 2006;101(7):1003-13.R10XP02/3; 
Courtenay-Quirk C, Pals SL, Kidder DP, Henny K, Emshoff JG. Factors associated with incarceration history among HIV-positive persons experiencing homelessness or imminent risk of homelessness. Journal of Community Health 2008;33(6):434-43.R14XP02/2; 
Crepaz N, Lyles CM, Wolitski RJ, Passin WF, Rama SM, Herbst JH et al. Do prevention interventions reduce HIV risk behaviours among people living with HIV? A meta-analytic review of controlled trials. [Review] [79 refs]. AIDS 2006;20(2):143-57.R14XP02/2; 
Crepaz N, Passin WF, Herbst JH, Rama SM, Malow RM, Purcell DW et al. Meta-analysis of cognitive-behavioral interventions on HIV-positive persons' mental health and immune functioning. Health Psychology 2008;27(1):4-14.R14XP02/2; 
Crepaz N, Horn AK, Rama SM, Griffin T, Deluca JB, Mullins MM et al. The efficacy of behavioral interventions in reducing HIV risk sex behaviors and incident sexually transmitted disease in black and Hispanic sexually transmitted disease clinic patients in the United States: a meta-analytic review.[see comment]. [Review] [58 refs]. Sexually Transmitted Diseases 2007;34(6):319-32.R03XP02/1; R04XP02/1.
Darbes L, Crepaz N, Lyles C, Kennedy G, Rutherford G. The efficacy of behavioral interventions in reducing HIV risk behaviors and incident sexually transmitted diseases in heterosexual African Americans.[erratum appears in AIDS. 2008 Jul 31;22(12):i]. [Review] [78 refs]. AIDS 2008;22(10):1177-94.R03XP02/3; 
Daykin N, Orme J, Evans D, Salmon D, McEachran M, Brain S. The impact of participation in performing arts on adolescent health and behaviour: a systematic review of the literature (DARE structured abstract). Journal of Health Psychology 2008;13:251-64.R05XP02/1; 
de GR, Leonard NR, Gwadz MV, Young R, Ritchie AS, Arredondo G et al. "I thought there was no hope for me": a behavioral intervention for urban mothers with problem drinking. Qualitative Health Research 2006;16(9):1252-66.R08XP02/3; R10XP02/3.
De SJ. Exploring the concepts of vulnerability and resilience in the context of HIV infection. [Review] [35 refs]. Research & Theory for Nursing Practice 2008;22(4):273-87.R01XP02/3; 
DiIorio C, McCarty F, Denzmore P. An exploration of social cognitive theory mediators of father-son communication about sex. Journal of Pediatric Psychology 2006;31(9):917-27.R05XP02/1; R07XP02/1.
DiIorio C, Resnicow K, McCarty F, De AK, Dudley WN, Wang DT et al. Keepin' it R.E.A.L.!: results of a mother-adolescent HIV prevention program. Nursing Research 2006;55(1):43-51.R05XP02/1; 
DiIorio C, McCarty F, Resnicow K, Lehr S, Denzmore P. REAL men: a group-randomized trial of an HIV prevention intervention for adolescent boys.[erratum appears in Am J Public Health. 2007 Aug;97(8):1350]. American Journal of Public Health 2007;97(6):1084-9.R05XP02/1; R07XP02/1.
Dilley JW, Loeb L, Marson K, Chen S, Schwarcz S, Paul J et al. Sexual compulsiveness and change in unprotected anal intercourse: unexpected results from a randomized controlled HIV counseling intervention study. Journal of Acquired Immune Deficiency Syndromes: JAIDS 2008;48(1):113-4.R16XP02/1; 
Durantini MR, Albarracin D, Mitchell AL, Earl AN, Gillette JC. Conceptualizing the Influence of Social Agents of Behavior Change: A Meta-Analysis of the Effectiveness of HIV-Prevention Interventionists for Different Groups. Psychological Bulletin 2006;132(2):212-48.R16XP02/3; 
Dworkin SL, Exner T, Melendez R, Hoffman S, Ehrhardt AA. Revisiting "Success": Posttrial analysis of a gender-specific HIV/STD prevention intervention. AIDS & Behavior 2006;10(1):41-51.R07XP02/1; 
Dworkin SL, Beckford ST, Ehrhardt AA. Sexual scripts of women: a longitudinal analysis of participants in a gender-specific HIV/STD prevention intervention. Archives of Sexual Behavior 2007;36(2):269-79.R08XP02/1; 
Fife BL, Scott LL, Fineberg NS, Zwickl BE. Promoting adaptive coping by persons with HIV disease: evaluation of a patient/partner intervention model. Journal of the Association of Nurses in AIDS Care 2008;19(1):75-84.R14XP02/2; 
Fischer B, Manzoni P, Rehm J. Comparing injecting and non-injecting illicit opioid users in a multisite Canadian sample (OPICAN Cohort). European Addiction Research 2006;12(4):230-9.R10XP02/3; 
Fisher JD, Fisher WA, Cornman DH, Amico RK, Bryan A, Friedland GH. Clinician-delivered intervention during routine clinical care reduces unprotected sexual behavior among HIV-infected patients. Journal of Acquired Immune Deficiency Syndromes: JAIDS 2006;41(1):44-52.R14XP02/2; 
Friedman SR, Mateu-Gelabert P, Sandoval M, Hagan H, Des J. Positive deviance control-case life history: a method to develop grounded hypotheses about successful long-term avoidance of infection. BMC Public Health 2008;8:94.R10XP02/1; 
Garfein RS, Golub ET, Greenberg AE, Hagan H, Hanson DL, Hudson SM et al. A peer-education intervention to reduce injection risk behaviors for HIV and hepatitis C virus infection in young injection drug users.[see comment]. AIDS 2007;21(14):1923-32.R10XP02/1; R05XP02/1.
Garfein RS, Golub ET, Greenberg AE, Hagan H, Hanson DL. A peer-education intervention to reduce injection risk behaviours for HIV and hepatitis C virus infection in young people injection drug users. AIDS 2007;21(14):1923-32.R10XP02/1; R05XP02/1.
Garnett GP, Garcia-Calleja JM, Rehle T, Gregson S. Behavioural data as an adjunct to HIV surveillance data. [Review] [46 refs]. Sexually Transmitted Infections 2006;82 Suppl 1:i57-i62.R16XP02/3; 
Gilbert L, El-Bassel N, Manuel J, Wu E, Go H, Golder S et al. An integrated relapse prevention and relationship safety intervention for women on methadone: Testing short-term effects on intimate partner violence and substance use. Violence and Victims 2006;21(5):657-72.R10XP02/3; 
Gilbert P, Ciccarone D, Gansky SA, Bangsberg DR, Clanon K, McPhee SJ et al. Interactive "Video Doctor" counseling reduces drug and sexual risk behaviors among HIV-positive patients in diverse outpatient settings. PLoS ONE [Electronic Resource] 2008;3(4):e1988.R14XP02/2; R14XP04/2.
Glenn BL, Demi A, Kimble LP. Father and adolescent son variable related to son's HIV prevention. Western Journal of Nursing Research 2008;30(1):73-89.R05XP02/1; R03XP02/1.
Goldberg E, Millson P, Rivers S, Manning SJ, Leslie K, Read S et al. A human immunodeficiency virus risk reduction intervention for incarcerated youth: a randomized controlled trial. Journal of Adolescent Health 2009;44(2):136-45.R05XP02/1; 
Gorbach PM, Drumright LN, Daar ES, Little SJ. Transmission behaviors of recently HIV-infected men who have sex with men. Journal of Acquired Immune Deficiency Syndromes: JAIDS 2006;42(1):80-5.R14XP02/2; 
Grimley DM, Bachmann LH, Jenckes MW, Erbelding EJ. Provider-delivered, theory-based, individualized prevention interventions for HIV positive adults receiving HIV comprehensive care. AIDS & Behavior 2007;11(5 Suppl):S39-S47.R14XP02/2; 
Hansen NB, Tarakeshwar N, Ghebremichael M, Zhang H, Kochman A, Sikkema KJ. Longitudinal effects of coping on outcome in a randomized controlled trial of a group intervention for HIV-positive adults with AIDS-related bereavement. Death Studies 2006;30(7):609-36.R14XP02/2; 
Hanson T, Alessi SM, Petry NM. Contingency management reduces drug-related human immunodeficiency virus risk behaviors in cocaine-abusing methadone patients. Addiction 103(7)()(pp 1187-1197), 2008 Date of Publication: Jul 2008 2008;(7):1187-97.R10XP02/3; 
Healthy Living Project Team. Effects of a behavioral intervention to reduce risk of transmission among people living with HIV: the healthy living project randomized controlled study. Journal of Acquired Immune Deficiency Syndromes: JAIDS 2007;44(2):213-21.R14XP02/2; 
Herbst JH, Kay LS, Passin WF, Lyles CM, Crepaz N, Marin BV et al. A systematic review and meta-analysis of behavioral interventions to reduce HIV risk behaviors of Hispanics in the United States and Puerto Rico. AIDS & Behavior 2007;11(1):25-47.R04XP02/3; 
Himelhoch S, Medoff DR, Oyeniyi G. Efficacy of group psychotherapy to reduce depressive symptoms among HIV-infected individuals: A systematic review and meta-analysis. AIDS Patient Care and STDs 21(10)()(pp 732-739), 2007 Date of Publication: 01 Oct 2007 2007;(10):732-9.R14XP02/2; 
Hoffman S, Beckford Jarrett ST, Kelvin EA, Wallace SA, Augenbraun M, Hogben M et al. HIV and sexually transmitted infection risk behaviors and beliefs among Black West Indian immigrants and US-born Blacks. American Journal of Public Health 2008;98(11):2042-50.R03XP02/3; 
Ito KE, Kalyanaraman S, Ford CA, Brown JD, Miller WC. "Let's Talk About Sex": pilot study of an interactive CD-ROM to prevent HIV/STIS in female adolescents. AIDS Education & Prevention 2008;20(1):78-89.R05XP02/1; R08XP02/1.
Jemmott LS, Jemmott JB, III, O'Leary A. Effects on sexual risk behavior and STD rate of brief HIV/STD prevention interventions for African American women in primary care settings. American Journal of Public Health 2007;97(6):1034-40.R03XP02/1; 
Jemmott LS, Jemmott JB, Hutchinson MK, Cederbaum JA, O'Leary A. Sexually transmitted infection/HIV risk reduction interventions in clinical practice settings. [Review] [43 refs]. JOGNN - Journal of Obstetric, Gynecologic, & Neonatal Nursing 2008;37(2):137-45.R03XP02/3; R08XP02/3.
Jin F, Crawford J, Prestage GP, Zablotska I, Imrie J, Kippax SC et al. Unprotected anal intercourse, risk reduction behaviours, and subsequent HIV infection in a cohort of homosexual men.[see comment]. AIDS 2009;23(2):243-52.R01XP02/3; 
Johnson BT, Carey MP, Chaudoir SR, Reid AE. Sexual risk reduction for persons living with HIV: Research synthesis of randomized controlled trials, 1993 to 2004. Journal of Acquired Immune Deficiency Syndromes 41(5)()(pp 642-650), 2006 Date of Publication: Apr 2006 2006;(5):642-50.R14XP02/2; 
Johnson BT, Carey MP, Chaudoir SR, Reid AE. Sexual risk reduction for persons living with HIV: research synthesis of randomized controlled trials, 1993 to 2004. [Review] [44 refs]. Journal of Acquired Immune Deficiency Syndromes: JAIDS 2006;41(5):642-50.R01XP02/3; 
Johnson WD, Diaz RM, Flanders WD, Goodman M, Hill AN, Holtgrave D et al. Behavioral interventions to reduce risk for sexual transmission of HIV among men who have sex with men.[update of Cochrane Database Syst Rev. 2003;(1):CD001230; PMID: 12535405]. [Review] [245 refs]. Cochrane Database of Systematic Reviews 2008;(3):CD001230.R01XP02/1; 
Kalichman SC, Cherry C, White D, Pope H, Cain D, Kalichman M. Altering key characteristics of a disseminated effective behavioral intervention for HIV positive adults: the "healthy relationships" experience. Journal of Primary Prevention 2007;28(2):145-53.R14XP02/2; 
Kelly C, Alderdice F, Lohan M. Psychological challenges of testing positive for HIV during pregnancy. British Journal of Midwifery 2009;17(2):76-81.R02XP02/3; 
Kershaw TS, Milan S, Westdahl C, Lewis J, Rising SS, Fletcher R et al. Avoidance, anxiety, and sex: the influence of romantic attachment on HIV-risk among pregnant women. AIDS & Behavior 2007;11(2):299-311.R13XP02/1; 
Khan MR, Miller WC, Schoenbach VJ, Weir SS, Kaufman JS, Wohl DA et al. Timing and duration of incarceration and high-risk sexual partnerships among African Americans in North Carolina. Annals of Epidemiology 2008;18(5):403-10.R03XP02/3; R13XP02/3.
Kiene SM, Barta WD. A brief individualized computer-delivered sexual risk reduction intervention increases HIV/AIDS preventive behavior. Journal of Adolescent Health 2006;39(3):404-10.R05XP02/3; 
King VL, Kidorf MS, Peirce J, Brooner RK. Result from a controlled trial of a motivational intervention for improving treatment enrollment among needle-exchange participants. 69th Annual Scientific Meeting of the College on Problems of Drug Dependence; 2007 Jun 16 21; Quebec, Canada 2007.R10XP02/3; 
Koblin BA, Husnik MJ, Colfax G, Huang Y, Madison M, Mayer K et al. Risk factors for HIV infection among men who have sex with men. AIDS 2006;20(5):731-9.R01XP02/1; 
Koenig LJ, Whitaker DJ, Royce RA, Wilson TE, Ethier K, Fernandez MI. Physical and sexual violence during pregnancy and after delivery: a prospective multistate study of women with or at risk for HIV infection. American Journal of Public Health 2006;96(6):1052-9.R02XP02/1; R15XP02/1.
Korner H, Hendry O, Kippax S. Safe sex after post-exposure prophylaxis for HIV: intentions, challenges and ambivalences in narratives of gay men. AIDS Care 2006;18(8):879-87.R01XP02/1; 
Kraft C, Robinson BB, Nordstrom DL, Bockting WO, Rosser BR. Obesity, body image, and unsafe sex in men who have sex with men. Archives of Sexual Behavior 2006;35(5):587-95.R01XP02/1; 
Kraft JM, Harvey SM, Thorburn S, Henderson JT, Posner SF, Galavotti C. Intervening with couples: assessing contraceptive outcomes in a randomized pregnancy and HIV/STD risk reduction intervention trial. Womens Health Issues 2007;17(1):52-60.R09XP02/3; 
Latkin C, Donnell D, Celentano DD, Aramrattna A, Liu TY, Vongchak T et al. Relationships between social norms, social network characteristics, and HIV risk behaviors in Thailand and the United States. Health Psychology 2009;28(3):323-9.R10XP02/1; 
Lesser J, Koniak-Griffin D, Gonzalez-Figueroa E, Huang R, Cumberland WG. Childhood abuse history and risk behaviors among teen parents in a culturally rooted, couple-focused HIV prevention program. Journal of the Association of Nurses in AIDS Care 2007;18(2):18-27.R05XP02/1; 
Lewis MA, Gladstone E, Schmal S, Darbes LA. Health-related social control and relationship interdependence among gay couples. Health Education Research 2006;21(4):488-500.R01XP02/3; 
Liau A, Millett G, Marks G. Meta-analytic examination of online sex-seeking and sexual risk behavior among men who have sex with men. Sexually Transmitted Diseases 2006;33(9):576-84.R01XP02/3; 
Lovejoy TI, Heckman TG, Sikkema KJ, Hansen NB, Kochman A, Suhr JA et al. Patterns and correlates of sexual activity and condom use behavior in persons 50-plus years of age living with HIV/AIDS. AIDS & Behavior 2008;12(6):943-56.R14XP02/2; 
Lyles CM, Kay LS, Crepaz N, Herbst JH, Passin WF, Kim AS et al. Best-evidence interventions: findings from a systematic review of HIV behavioral interventions for US populations at high risk, 2000-2004. [Review] [114 refs]. American Journal of Public Health 2007;97(1):133-43.R16XP02/3; 
Macdonald N, Elam G, Hickson F, Imrie J, McGarrigle CA, Fenton KA et al. Factors associated with HIV seroconversion in gay men in England at the start of the 21st century. Sexually Transmitted Infections 2008;84(1):8-13.R01XP02/3; 
Margolin A, Beitel M, Schuman-Olivier Z, Avants SK. A controlled study of a spirituality-focused intervention for increasing motivation for HIV prevention among drug users. AIDS Education & Prevention 2006;18(4):311-22.R10XP02/1; 
Marston C, King E. Factors that shape young people's sexual behaviour: a systematic review. Lancet 2006;368(9547):1581-6.R05XP02/3; 
Martin SS, O'Connell DJ, Inciardi JA, Surratt HL, Maiden KM. Integrating an HIV/HCV brief intervention in prisoner reentry: results of a multisite prospective study. Journal of Psychoactive Drugs 2008;40(4):427-36.R13XP02/3; 
Mausbach BT, Semple SJ, Strathdee SA, Zians J, Patterson TL. Efficacy of a behavioral intervention for increasing safer sex behaviors in HIV-negative, heterosexual methamphetamine users: results from the Fast-Lane Study. Annals of Behavioral Medicine 2007;34(3):263-74.R10XP02/1; R09XP02/1.
Mausbach BT, Semple SJ, Strathdee SA, Zians J, Patterson TL. Efficacy of a behavioral intervention for increasing safer sex behaviors in HIV-positive MSM methamphetamine users: results from the EDGE study. Drug & Alcohol Dependence 2007;87(2-3):249-57.R01XP02/2; R10XP02/2.
McCain NL, Gray DP, Elswick RK, Robins JW, Tuck I, Walter JM et al. A randomized clinical trial of alternative stress management interventions in persons with HIV infection. Journal of Consulting & Clinical Psychology 2008;76(3):431-41.R14XP02/2; 
Morgenstern J, Irwin TW, Wainberg ML, Parsons JT, Muench F, Bux J et al. A randomized controlled trial of goal choice interventions for alcohol use disorders among men who have sex with men. Journal of Consulting and Clinical Psychology 75(1)()(pp 72-84), 2007 Date of Publication: Feb 2007 2007;(1):72-84.R01XP02/3; R10XP02/3.
Morin SF, Shade SB, Steward WT, Carrico AW, Remien RH, Rotheram-Borus MJ et al. A behavioral intervention reduces HIV transmission risk by promoting sustained serosorting practices among HIV-infected men who have sex with men. Journal of Acquired Immune Deficiency Syndromes: JAIDS 2008;49(5):544-51.R14XP02/2; 
Morrison DM, Casey EA, Beadnell BA, Hoppe MJ, Gillmore MR, Wilsdon A et al. Effects of friendship closeness in an adolescent group HIV prevention intervention. Prevention Science 2007;8(4):274-84.R05XP02/1; 
Mosack KE, Weinhardt LS, Kelly JA, Gore-Felton C, McAuliffe TL, Johnson MO et al. Influence of coping, social support, and depression on subjective health status among HIV-positive adults with different sexual identities. Behavioral Medicine 2009;34(4):133-44.R01XP02/2; 
Naar-King S, Lam P, Wang B, Wright K, Parsons JT, Frey MA. Brief report: maintenance of effects of motivational enhancement therapy to improve risk behaviors and HIV-related Health in a randomized controlled trial of youth living with HIV. Journal of Pediatric Psychology 2008;33(4):441-5.R14XP02/2; R05XP02/2.
Naar-King S, Wright K, Parsons JT, Frey M, Templin T, Lam P et al. Healthy choices: motivational enhancement therapy for health risk behaviors in HIV-positive youth. AIDS Education & Prevention 2006;18(1):1-11.R14XP02/2; 
National Institute for Health and Clinical Excellence. One to one interventions to reduce the transmission of sexually transmitted infections (STIs) including HIV, and to reduce the rate of under 18 conceptions, especially among vulnerable and at risk groups (DARE structured abstract). London: National Institute for Health and Clinical Excellence (NICE) 2007;50.R05XP02/1; 
NIMH Multisite HIV/STD Prevention Trial for African American Couples Group. Eban health promotion intervention: conceptual basis and procedures. Journal of Acquired Immune Deficiency Syndromes: JAIDS 2008;49 Suppl 1:S28-S34.R03XP02/3; 
NIMH Multisite HIV/STD Prevention Trial for African American Couples Group. Eban HIV/STD risk reduction intervention: conceptual basis and procedures. Journal of Acquired Immune Deficiency Syndromes: JAIDS 2008;49 Suppl 1:S15-S27.R03XP02/3; 
NIMH Multisite HIV/STD Prevention Trial for African American Couples Group. Formative study to develop the Eban treatment and comparison interventions for couples. Journal of Acquired Immune Deficiency Syndromes: JAIDS 2008;49 Suppl 1:S42-S51.R03XP02/3; 
NIMH Multisite HIV/STD Prevention Trial for African American Couples Group. Measure of HIV/STD risk-reduction: strategies for enhancing the utility of behavioral and biological outcome measures for African American couples. Journal of Acquired Immune Deficiency Syndromes: JAIDS 2008;49 Suppl 1:S35-S41.R03XP02/3; 
Noar SM. Behavioral interventions to reduce HIV-related sexual risk behavior: review and synthesis of meta-analytic evidence. [Review] [85 refs]. AIDS & Behavior 2008;12(3):335-53.R16XP02/3; 
Noar SM, Black HG, Pierce LB. Efficacy of computer technology-based HIV prevention interventions: a meta-analysis. [Review] [49 refs]. AIDS 2009;23(1):107-15.R16XP02/3; 
O'Dell BL, Rosser BR, Miner MH, Jacoby SM. HIV prevention altruism and sexual risk behavior in HIV-positive men who have sex with men. AIDS & Behavior 2008;12(5):713-20.R14XP02/2; 
O'Leary A, Jemmott LS, Jemmott JB. Mediation analysis of an effective sexual risk-reduction intervention for women: the importance of self-efficacy. Health Psychology 2008;27(2 Suppl):S180-S184.R03XP02/1; R08XP02/1.
Parsons JT, Golub SA, Rosof E, Holder C. Motivational interviewing and cognitive-behavioral intervention to improve HIV medication adherence among hazardous drinkers: a randomized controlled trial. Journal of Acquired Immune Deficiency Syndromes: JAIDS 2007;46(4):443-50.R14XP02/2; R10XP02/2.
Picciano JF, Roffman RA, Kalichman SC, Walker DD. Lowering obstacles to HIV prevention services: effects of a brief, telephone-based intervention using motivational enhancement therapy. Annals of Behavioral Medicine 2007;34(2):177-87.R01XP02/1; 
Prestage G, Mao L, McGuigan D, Crawford J, Kippax S, Kaldor J et al. HIV risk and communication between regular partners in a cohort of HIV-negative gay men. AIDS Care 2006;18(2):166-72.R01XP02/1; 
Prost A, Elford J, Imrie J, Petticrew M, Hart GJ. Social, behavioural, and intervention research among people of Sub-Saharan African origin living with HIV in the UK and Europe: literature review and recommendations for intervention. [Review] [22 refs]. AIDS & Behavior 2008;12(2):170-94.R03XP02/3; 
Purcell DW, Garfein RS, Latka MH, Thiede H, Hudson S, Bonner S et al. Development, description, and acceptability of a small-group, behavioral intervention to prevent HIV and hepatitis C virus infections among young adult injection drug users. Drug & Alcohol Dependence 2007;91 Suppl 1:S73-S80.R05XP02/1; R10XP02/1.
Purcell DW, Latka MH, Metsch LR, Latkin CA, Gomez CA, Mizuno Y et al. Results from a randomized controlled trial of a peer-mentoring intervention to reduce HIV transmission and increase access to care and adherence to HIV medications among HIV-seropositive injection drug users. Journal of Acquired Immune Deficiency Syndromes: JAIDS 2007;46 Suppl 2:S35-S47.R14XP02/2; 
Raj A, Cheng DM, Levison R, Meli S, Samet JH. Sex trade, sexual risk, and nondisclosure of HIV serostatus: findings from HIV-infected persons with a history of alcohol problems. AIDS & Behavior 2006;10(2):149-57.R14XP02/2; 
Rawstorne P, Fogarty A, Crawford J, Prestage G, Grierson J, Grulich A et al. Differences between HIV-positive gay men who 'frequently', 'sometimes' or 'never' engage in unprotected anal intercourse with serononconcordant casual partners: Positive Health cohort, Australia. AIDS Care - Psychological and Socio-Medical Aspects of AIDS/HIV 2007;19(4):514-22.R14XP02/2; 
Read TR, Hocking J, Sinnott V, Hellard M. Risk factors for incident HIV infection in men having sex with men: a case-control study. Sexual Health 2007;4(1):35-9.R01XP02/1; 
Rebchook GM, Kegeles SM, Huebner D, TRIP Research Team. Translating research into practice: the dissemination and initial implementation of an evidence-based HIV prevention program. AIDS Education & Prevention 2006;18(4 Suppl A):119-36.R01XP02/3; 
Rhodes F, Stein JA, Fishbein M, Goldstein RB, Rotheram-Borus MJ. Using Theory to Understand How Interventions Work: Project RESPECT, Condom Use, and the Integrative Model. Aids Behav 2007;11(3):393-407.R16XP02/3; 
Rhodes SD, McCoy T, Omli MR, Cohen G, Champion H, DuRant RH. Who really uses condoms? Findings from a large internet-recruited random sample of unmarried heterosexual college students in the Southeastern United States. Journal of HIV/AIDS Prevention in Children and Youth 7(2)()(pp 9-27), 2007 Date of Publication: 12 May 2007 2007;(2):9-27.R09XP02/1; 
Rietmeijer CA, Lloyd LV, McLean C. Discussing HIV serostatus with prospective sex partners: a potential HIV prevention strategy among high-risk men who have sex with men.[see comment]. Sexually Transmitted Diseases 2007;34(4):215-9.R01XP02/3; 
Roberto AJ, Zimmerman RS, Carlyle KE, Abner EL. A computer-based approach to preventing pregnancy, STD, and HIV in rural adolescents. Journal of Health Communication 2007;12(1):53-76.R05XP02/1; R05XP04/1.
Rosen MI, Dieckhaus K, McMahon TJ, Valdes B, Petry NM, Cramer J et al. Improved adherence with contingency management. AIDS Patient Care & Stds 2007;21(1):30-40.R14XP02/2; 
Ross MW, Rosser BR, Neumaier ER, Positive Connections Team. The relationship of internalized homonegativity to unsafe sexual behavior in HIV-seropositive men who have sex with men. AIDS Education & Prevention 2008;20(6):547-57.R01XP02/2; R03XP02/2.
Rotheram-Borus MJ. HIV prevention with persons with mental health problems. Psychology, Health and Medicine 11(2)()(pp 142-154), 2006 Date of Publication: May 2006 2006;(2):142-54.R15XP02/1; 
Rotheram-Borus MJ, Lester P, Song J, Lin YY, Leonard NR, Beckwith L et al. Intergenerational benefits of family-based HIV interventions. Journal of Consulting & Clinical Psychology 2006;74(3):622-7.R05XP02/1; 
Rotheram-Borus MJ, Desmond K, Comulada WS, Arnold EM, Johnson M, Healthy Living Trial Group. Reducing risky sexual behavior and substance use among currently and formerly homeless adults living with HIV. American Journal of Public Health 2009;99(6):1100-7.R14XP02/2; R10XP02/2.
Rowe CL, Wang W, Greenbaum P, Liddle HA. Predicting HIV/STD risk level and substance use disorders among incarcerated adolescents. Journal of Psychoactive Drugs 2008;40(4):503-12.R05XP02/1; R13XP02/1.
Roye C, Perlmutter SP, Krauss B. A brief, low-cost, theory-based intervention to promote dual method use by black and Latina female adolescents: a randomized clinical trial. Health Education & Behavior 2007;34(4):608-21.R08XP02/3; R04XP02/3.
Saewyc E, Skay C, Richens K, Reis E, Poon C, Murphy A. Sexual orientation, sexual abuse, and HIV-risk behaviors among adolescents in the Pacific Northwest. American Journal of Public Health 2006;96(6):1104-10.R15XP02/1; 
Satcher AJ, Durant T, Hu X, Dean HD. AIDS cases among women who reported sex with a bisexual man, 2000-2004--United States. Women & Health 2007;46(2-3):23-40.R08XP02/1; 
Schilder AJ, Orchard TR, Buchner CS, Miller ML, Fernandes KA, Hogg RS et al. 'It's like the treasure': beliefs associated with semen among young HIV-positive and HIV-negative gay men. Culture, Health & Sexuality 2008;10(7):667-79.R01XP02/3; 
Schmiege SJ, Broaddus MR, Levin M, Bryan AD. Randomized trial of group interventions to reduce HIV/STD risk and change theoretical mediators among detained adolescents. Journal of Consulting & Clinical Psychology 2009;77(1):38-50.R13XP02/1; R05XP02/1.
Schroeder JR, Epstein DH, Umbricht A, Preston KL. Changes in HIV risk behaviors among patients receiving combined pharmacological and behavioral interventions for heroin and cocaine dependence. Addictive Behaviors 2006;31(5):868-79.R10XP02/3; 
Scott-Sheldon LA, Johnson BT. Eroticizing creates safer sex: a research synthesis. [Review] [67 refs]. Journal of Primary Prevention 2006;27(6):619-40.R16XP02/3; 
Serovich JM, Reed S, Grafsky EL, Andrist D. An intervention to assist men who have sex with men disclose their serostatus to casual sex partners: results from a pilot study. AIDS Education & Prevention 2009;21(3):207-19.R01XP02/2; 
Setswe G. Abstinence and faithfulness programmes for prevention of HIV/AIDS among young people: What are the current debates? South African Family Practice 49(8)()(pp 5-10), 2007 Date of Publication: Sep 2007 2007;(8):5-10.R16XP02/3; 
Sikkema KJ, Wilson PA, Hansen NB, Kochman A, Neufeld S, Ghebremichael MS et al. Effects of a coping intervention on transmission risk behavior among people living with HIV/AIDS and a history of childhood sexual abuse. Journal of Acquired Immune Deficiency Syndromes: JAIDS 2008;47(4):506-13.R14XP02/2; R15XP02/2.
Sikkema KJ, Hansen NB, Kochman A, Tarakeshwar N, Neufeld S, Meade CS et al. Outcomes from a group intervention for coping with HIV/AIDS and childhood sexual abuse: reductions in traumatic stress. AIDS & Behavior 2007;11(1):49-60.R14XP02/2; 
Smith R, Rossetto K, Peterson BL. A meta-analysis of disclosure of one's HIV-positive status, stigma and social support. [Review] [75 refs]. AIDS Care 2008;20(10):1266-75.R14XP02/2; 
Smoak ND, Scott-Sheldon LA, Johnson BT, Carey MP. Sexual risk reduction interventions do not inadvertently increase the overall frequency of sexual behavior: a meta-analysis of 174 studies with 116,735 participants. Journal of Acquired Immune Deficiency Syndromes: JAIDS 2006;41(3):374-84.R16XP02/3; 
Sterk CE, Theall KP, Elifson KW. The impact of emotional distress on HIV risk reduction among women. Substance Use & Misuse 2006;41(2):157-73.R03XP02/1; R10XP02/1.
Taylor MM, Aynalem G, Smith LV, Montoya J, Kerndt P. Methamphetamine use and sexual risk behaviours among men who have sex with men diagnosed with early syphilis in Los Angeles County. International Journal of STD and AIDS 2007;18(2):93-7.R01XP02/3; R10XP02/3.
The National Institute Of Mental Health Multisite HIV Prevention Trial Group. HIV prevention with persons with mental health problems. Psychology Health & Medicine 2006;11(2):142-54.R15XP02/1; 
Theall KP, Elifson KW, Sterk CE. Sex, touch, and HIV risk among ecstasy users. AIDS and Behavior 2006;10(2):169-78.R10XP02/1; 
Thurstone C, Riggs PD, Klein C, Mikulich-Gilbertson SK. A one-session human immunodeficiency virus risk-reduction intervention in adolescents with psychiatric and substance use disorders. Journal of the American Academy of Child & Adolescent Psychiatry 2007;46(9):1179-86.R05XP02/1; R10XP02/1.
Tross S, Campbell AN, Cohen LR, Calsyn D, Pavlicova M, Miele GM et al. Effectiveness of HIV/STD sexual risk reduction groups for women in substance abuse treatment programs: results of NIDA Clinical Trials Network Trial. Journal of Acquired Immune Deficiency Syndromes: JAIDS 2008;48(5):581-9.R08XP02/1; R10XP02/1.
Underhill K, Operario D, Montgomery P. Abstinence-only programs for HIV infection prevention in high-income countries. Cochrane Database of Systematic Reviews (4), 2007 Article Number: CD005421 Date of Publication: 2007 2007;(4).R16XP02/1; 
van Kesteren NM, Hospers HJ, Kok G. Sexual risk behavior among HIV-positive men who have sex with men: a literature review.[see comment]. [Review] [74 refs]. Patient Education & Counseling 2007;65(1):5-20.R01XP02/2; 
Vanable PA, Carey MP, Carey KB, Maisto SA. Differences in HIV-related knowledge, attitudes, and behavior among psychiatric outpatients with and without a history of a sexually transmitted infection. Journal of Prevention & Intervention in the Community 2007;33(1-2):79-94.R15XP02/1; 
Villarruel AM, Cherry CL, Cabriales EG, Ronis DL, Zhou Y. A parent-adolescent intervention to increase sexual risk communication: results of a randomized controlled trial. AIDS Education & Prevention 2008;20(5):371-83.R05XP02/1; 
Villarruel AM, Jemmott JB, III, Jemmott LS. A randomized controlled trial testing an HIV prevention intervention for Latino youth.[erratum appears in Arch Pediatr Adolesc Med. 2006 Nov;160(11):1187]. Archives of Pediatrics & Adolescent Medicine 2006;160(8):772-7.R05XP02/1; 
Volk JE, Prestage G, Jin F, Kaldor J, Ellard J, Kippax S et al. Risk factors for HIV seroconversion in homosexual men in Australia. Sexual Health 2006;3(1):45-51.R14XP02/2; 
Volkow ND, Wang GJ, Fowler JS, Telang F, Jayne M, Wong C. Stimulant-induced enhanced sexual desire as a potential contributing factor in HIV transmission. American Journal of Psychiatry 2007;164(1):157-60.R10XP02/3; 
Weir BW, O'Brien K, Bard RS, Casciato CJ, Maher JE, Dent CW et al. Reducing HIV and partner violence risk among women with criminal justice system involvement: a randomized controlled trial of two motivational interviewing-based interventions. AIDS & Behavior 2009;13(3):509-22.R08XP02/1; R15XP02/1.
Widdice LE, Cornell JL, Liang W, Halpern-Felsher BL. Having sex and condom use: potential risks and benefits reported by young, sexually inexperienced adolescents. Journal of Adolescent Health  2006;39(4):588-95.R05XP02/1; 
Wilson TE, Feldman J, Vega MY, Gandhi M, Richardson J, Cohen MH et al. Acquisition of new sexual partners among women with HIV infection: patterns of disclosure and sexual behavior within new partnerships. AIDS Education & Prevention 2007;19(2):151-9.R14XP02/2; 
Wilton L, Herbst JH, Coury-Doniger P, Painter TM, English G, Alvarez ME et al. Efficacy of an HIV/STI prevention intervention for black men who have sex with men: findings from the Many Men, Many Voices (3MV) project. AIDS & Behavior 2009;13(3):532-44.R01XP02/1; R03XP02/1.
Wingood GM, DiClemente RJ, Harrington KF, Lang DL, Davies SL, Hook EW, III et al. Efficacy of an HIV prevention program among female adolescents experiencing gender-based violence. American Journal of Public Health 2006;96(6):1085-90.R03XP02/1; R08XP02/1.
Wong FL, Rotheram-Borus MJ, Lightfoot M, Pequegnat W, Comulada WS, Cumberland W et al. Effects of behavioral intervention on substance use among people living with HIV: the Healthy Living Project randomized controlled study. Addiction 2008;103(7):1206-14.R14XP02/2; 
Wright PB, Stewart KE, Fischer EP, Carlson RG, Falck R, Wang J et al. HIV risk behaviors among rural stimulant users: variation by gender and race/ethnicity. AIDS Education & Prevention 2007;19(2):137-50.R10XP02/3; 

2 codes
Bacon O, Lum P, Hahn J, Evans J, Davidson P, Moss A et al. Commercial sex work and risk of HIV infection among young drug-injecting men who have sex with men in San Francisco. Sexually Transmitted Diseases 2006;33(4):228-34.R01XP02/3; R12XP02/3.
Barry D, Weinstock J, Petry NM. Ethnic differences in HIV risk behaviors among methadone-maintained women receiving contingency management for cocaine use disorders. Drug & Alcohol Dependence 2008;98(1-2):144-53.R03XP02/1; R08XP02/1.
Berkman A, Cerwonka E, Sohler N, Susser E. A randomized trial of a brief HIV risk reduction intervention for men with severe mental illness.[see comment]. Psychiatric Services 2006;57(3):407-9.R01XP02/3; R15XP02/3.
Berkman A, Cerwonka E, Sohler N, Susser E. A randomised trial of a brief HIV risk reduction intervention for men with severe mental illness. Psychiatric Services 2006;57(3):407-9.R07XP02/3; R15XP02/3.
Bowen AM, Williams ML, Daniel CM, Clayton S. Internet based HIV prevention research targeting rural MSM: feasibility, acceptability, and preliminary efficacy. Journal of Behavioral Medicine 2008;31(6):463-77.R01XP02/1; R16XP02/1.
Brown EJ, Van HM. Risk behavior, perceptions of HIV risk, and risk-reduction behavior among a small group of rural African American women who use drugs. Journal of the Association of Nurses in AIDS Care 2006;17(5):42-50.R03XP02/1; R08XP02/1.
Bryan A, Ray LA, Cooper ML. Alcohol use and protective sexual behaviors among high-risk adolescents. Journal of Studies on Alcohol & Drugs 2007;68(3):327-35.R05XP02/3; R15XP02/3.
Burdon WM, De L, Prendergast ML. The NOBLE study: assesing the effectiveness of residential vs intensive outpatient prison-based treatment and inmates views on HIV/AIDS-realted behaviours and services. Proceedings of the 70th Annual Scientific Meeting of the College on Problems of Drug Dependence; 2008 June 14 19; San Juan, Puerto Rico, USA 2008;26.R10XP02/3; R13XP02/3.
Crepaz N, Horn AK, Rama SM, Griffin T, Deluca JB, Mullins MM et al. The efficacy of behavioral interventions in reducing HIV risk sex behaviors and incident sexually transmitted disease in black and Hispanic sexually transmitted disease clinic patients in the United States: a meta-analytic review.[see comment]. [Review] [58 refs]. Sexually Transmitted Diseases 2007;34(6):319-32.R03XP02/1; R04XP02/1.
de GR, Leonard NR, Gwadz MV, Young R, Ritchie AS, Arredondo G et al. "I thought there was no hope for me": a behavioral intervention for urban mothers with problem drinking. Qualitative Health Research 2006;16(9):1252-66.R08XP02/3; R10XP02/3.
DiIorio C, McCarty F, Denzmore P. An exploration of social cognitive theory mediators of father-son communication about sex. Journal of Pediatric Psychology 2006;31(9):917-27.R05XP02/1; R07XP02/1.
DiIorio C, McCarty F, Resnicow K, Lehr S, Denzmore P. REAL men: a group-randomized trial of an HIV prevention intervention for adolescent boys.[erratum appears in Am J Public Health. 2007 Aug;97(8):1350]. American Journal of Public Health 2007;97(6):1084-9.R05XP02/1; R07XP02/1.
Earl A, Albarracin D. Nature, decay, and spiraling of the effects of fear-inducing arguments and HIV counseling and testing: a meta-analysis of the short- and long-term outcomes of HIV-prevention interventions.[erratum appears in Health Psychol. 2007 Nov;26(6):815-6]. Health Psychology 2007;26(4):496-506.R16XP05/3; R16XP02/3.
Garfein RS, Golub ET, Greenberg AE, Hagan H, Hanson DL, Hudson SM et al. A peer-education intervention to reduce injection risk behaviors for HIV and hepatitis C virus infection in young injection drug users.[see comment]. AIDS 2007;21(14):1923-32.R10XP02/1; R05XP02/1.
Garfein RS, Golub ET, Greenberg AE, Hagan H, Hanson DL. A peer-education intervention to reduce injection risk behaviours for HIV and hepatitis C virus infection in young people injection drug users. AIDS 2007;21(14):1923-32.R10XP02/1; R05XP02/1.
Glenn BL, Demi A, Kimble LP. Father and adolescent son variable related to son's HIV prevention. Western Journal of Nursing Research 2008;30(1):73-89.R05XP02/1; R03XP02/1.
Ito KE, Kalyanaraman S, Ford CA, Brown JD, Miller WC. "Let's Talk About Sex": pilot study of an interactive CD-ROM to prevent HIV/STIS in female adolescents. AIDS Education & Prevention 2008;20(1):78-89.R05XP02/1; R08XP02/1.
Jemmott LS, Jemmott JB, Hutchinson MK, Cederbaum JA, O'Leary A. Sexually transmitted infection/HIV risk reduction interventions in clinical practice settings. [Review] [43 refs]. JOGNN - Journal of Obstetric, Gynecologic, & Neonatal Nursing 2008;37(2):137-45.R03XP02/3; R08XP02/3.
Khan MR, Miller WC, Schoenbach VJ, Weir SS, Kaufman JS, Wohl DA et al. Timing and duration of incarceration and high-risk sexual partnerships among African Americans in North Carolina. Annals of Epidemiology 2008;18(5):403-10.R03XP02/3; R13XP02/3.
Koenig LJ, Whitaker DJ, Royce RA, Wilson TE, Ethier K, Fernandez MI. Physical and sexual violence during pregnancy and after delivery: a prospective multistate study of women with or at risk for HIV infection. American Journal of Public Health 2006;96(6):1052-9.R02XP02/1; R15XP02/1.
Mausbach BT, Semple SJ, Strathdee SA, Zians J, Patterson TL. Efficacy of a behavioral intervention for increasing safer sex behaviors in HIV-negative, heterosexual methamphetamine users: results from the Fast-Lane Study. Annals of Behavioral Medicine 2007;34(3):263-74.R10XP02/1; R09XP02/1.
Mausbach BT, Semple SJ, Strathdee SA, Zians J, Patterson TL. Efficacy of a behavioral intervention for increasing safer sex behaviors in HIV-positive MSM methamphetamine users: results from the EDGE study. Drug & Alcohol Dependence 2007;87(2-3):249-57.R01XP02/2; R10XP02/2.
Morgenstern J, Irwin TW, Wainberg ML, Parsons JT, Muench F, Bux J et al. A randomized controlled trial of goal choice interventions for alcohol use disorders among men who have sex with men. Journal of Consulting and Clinical Psychology 75(1)()(pp 72-84), 2007 Date of Publication: Feb 2007 2007;(1):72-84.R01XP02/3; R10XP02/3.
Naar-King S, Lam P, Wang B, Wright K, Parsons JT, Frey MA. Brief report: maintenance of effects of motivational enhancement therapy to improve risk behaviors and HIV-related Health in a randomized controlled trial of youth living with HIV. Journal of Pediatric Psychology 2008;33(4):441-5.R14XP02/2; R05XP02/2.
O'Leary A, Jemmott LS, Jemmott JB. Mediation analysis of an effective sexual risk-reduction intervention for women: the importance of self-efficacy. Health Psychology 2008;27(2 Suppl):S180-S184.R03XP02/1; R08XP02/1.
Parsons JT, Golub SA, Rosof E, Holder C. Motivational interviewing and cognitive-behavioral intervention to improve HIV medication adherence among hazardous drinkers: a randomized controlled trial. Journal of Acquired Immune Deficiency Syndromes: JAIDS 2007;46(4):443-50.R14XP02/2; R10XP02/2.
Purcell DW, Garfein RS, Latka MH, Thiede H, Hudson S, Bonner S et al. Development, description, and acceptability of a small-group, behavioral intervention to prevent HIV and hepatitis C virus infections among young adult injection drug users. Drug & Alcohol Dependence 2007;91 Suppl 1:S73-S80.R05XP02/1; R10XP02/1.
Ross MW, Rosser BR, Neumaier ER, Positive Connections Team. The relationship of internalized homonegativity to unsafe sexual behavior in HIV-seropositive men who have sex with men. AIDS Education & Prevention 2008;20(6):547-57.R01XP02/2; R03XP02/2.
Rotheram-Borus MJ, Desmond K, Comulada WS, Arnold EM, Johnson M, Healthy Living Trial Group. Reducing risky sexual behavior and substance use among currently and formerly homeless adults living with HIV.  American Journal of Public Health 2009;99(6):1100-7.R14XP02/2; R10XP02/2.
Rowe CL, Wang W, Greenbaum P, Liddle HA. Predicting HIV/STD risk level and substance use disorders among incarcerated adolescents. Journal of Psychoactive Drugs 2008;40(4):503-12.R05XP02/1; R13XP02/1.
Roye C, Perlmutter SP, Krauss B. A brief, low-cost, theory-based intervention to promote dual method use by black and Latina female adolescents: a randomized clinical trial. Health Education & Behavior 2007;34(4):608-21.R08XP02/3; R04XP02/3.
Schmiege SJ, Broaddus MR, Levin M, Bryan AD. Randomized trial of group interventions to reduce HIV/STD risk and change theoretical mediators among detained adolescents. Journal of Consulting & Clinical Psychology 2009;77(1):38-50.R13XP02/1; R05XP02/1.
Sikkema KJ, Wilson PA, Hansen NB, Kochman A, Neufeld S, Ghebremichael MS et al. Effects of a coping intervention on transmission risk behavior among people living with HIV/AIDS and a history of childhood sexual abuse. Journal of Acquired Immune Deficiency Syndromes: JAIDS 2008;47(4):506-13.R14XP02/2; R15XP02/2.
Sterk CE, Theall KP, Elifson KW. The impact of emotional distress on HIV risk reduction among women. Substance Use & Misuse 2006;41(2):157-73.R03XP02/1; R10XP02/1.
Taylor MM, Aynalem G, Smith LV, Montoya J, Kerndt P. Methamphetamine use and sexual risk behaviours among men who have sex with men diagnosed with early syphilis in Los Angeles County. International Journal of STD and AIDS 2007;18(2):93-7.R01XP02/3; R10XP02/3.
Thurstone C, Riggs PD, Klein C, Mikulich-Gilbertson SK. A one-session human immunodeficiency virus risk-reduction intervention in adolescents with psychiatric and substance use disorders. Journal of the American Academy of Child & Adolescent Psychiatry 2007;46(9):1179-86.R05XP02/1; R10XP02/1.
Tross S, Campbell AN, Cohen LR, Calsyn D, Pavlicova M, Miele GM et al. Effectiveness of HIV/STD sexual risk reduction groups for women in substance abuse treatment programs: results of NIDA Clinical Trials Network Trial. Journal of Acquired Immune Deficiency Syndromes: JAIDS 2008;48(5):581-9.R08XP02/1; R10XP02/1.
Weir BW, O'Brien K, Bard RS, Casciato CJ, Maher JE, Dent CW et al. Reducing HIV and partner violence risk among women with criminal justice system involvement: a randomized controlled trial of two motivational interviewing-based interventions. AIDS & Behavior 2009;13(3):509-22.R08XP02/1; R15XP02/1.
Wilton L, Herbst JH, Coury-Doniger P, Painter TM, English G, Alvarez ME et al. Efficacy of an HIV/STI prevention intervention for black men who have sex with men: findings from the Many Men, Many Voices (3MV) project. AIDS & Behavior 2009;13(3):532-44.R01XP02/1; R03XP02/1.
Wingood GM, DiClemente RJ, Harrington KF, Lang DL, Davies SL, Hook EW, III et al. Efficacy of an HIV prevention program among female adolescents experiencing gender-based violence. American Journal of Public Health 2006;96(6):1085-90.R03XP02/1; R08XP02/1.

3 codes
Bacon O, Lum P, Hahn J, Evans J, Davidson P, Moss A et al. Commercial sex work and risk of HIV infection among young drug-injecting men who have sex with men in San Francisco. Sexually Transmitted Diseases 2006;33(4):228-34.R01XP02/3; R12XP02/3; R10XP02/3
Barry D, Weinstock J, Petry NM. Ethnic differences in HIV risk behaviors among methadone-maintained women receiving contingency management for cocaine use disorders. Drug & Alcohol Dependence 2008;98(1-2):144-53.R03XP02/1; R08XP02/1; R10XP02/1
Glenn BL, Demi A, Kimble LP. Father and adolescent son variable related to son's HIV prevention. Western Journal of Nursing Research 2008;30(1):73-89.R05XP02/1; R03XP02/1; R07XP02/1
Hallett TB, Garnett GP, Mupamberiyi Z, Gregson S. Measuring effectiveness in community randomized trials of HIV prevention. International Journal of Epidemiology 2008;37(1):77-87.R16XP10/1; R16XP13/1 - METHODOLOGY; R16XP02/1
Ross MW, Rosser BR, Neumaier ER, Positive Connections Team. The relationship of internalized homonegativity to unsafe sexual behavior in HIV-seropositive men who have sex with men. AIDS Education & Prevention 2008;20(6):547-57.R01XP02/2; R03XP02/2; R04XP02/2
Rotheram-Borus MJ, Desmond K, Comulada WS, Arnold EM, Johnson M, Healthy Living Trial Group. Reducing risky sexual behavior and substance use among currently and formerly homeless adults living with HIV. American Journal of Public Health 2009;99(6):1100-7.R14XP02/2; R10XP02/2; R13XP02/2
Sterk CE, Theall KP, Elifson KW. The impact of emotional distress on HIV risk reduction among women. Substance Use & Misuse 2006;41(2):157-73.R03XP02/1; R10XP02/1; R08XP02/1
Wingood GM, DiClemente RJ, Harrington KF, Lang DL, Davies SL, Hook EW, III et al. Efficacy of an HIV prevention program among female adolescents experiencing gender-based violence.  American Journal of Public Health 2006;96(6):1085-90.R03XP02/1; R08XP02/1; R15XP02/1

Law / Ethics / Policy (P03)
1 code
Eshel A, Moore A, Mishra M, Wooster J, Toledo C, Uhl G et al. Community stakeholders' perspectives on the impact of the minority AIDS initiative in strengthening HIV prevention capacity in four communities. Ethnicity & Health 2008;13(1):39-54.R04XP03/3; 
Shannon K, Strathdee SA, Shoveller J, Rusch M, Kerr T, Tyndall MW. Structural and environmental barriers to condom use negotiation with clients among female sex workers: implications for HIV-prevention strategies and policy. American Journal of Public Health 2009;99(4):659-65.R12XP03/3; 
2 codes
Hornberger J, Holodniy M, Robertus K, Winnike M, Gibson E, Verhulst E. A systematic review of cost-utility analyses in HIV/AIDS: implications for public policy. [Review] [128 refs]. Medical Decision Making 2007;27(6):789-821.R16XP13/3 - COST EFFECTIVENESS; R16XP03/3 - POLICY;

Service Delivery (P04)
1 code
Altice FL, Maru DS, Bruce RD, Springer SA, Friedland GH. Superiority of directly administered antiretroviral therapy over self-administered therapy among HIV-infected drug users: a prospective, randomized, controlled trial. Clinical Infectious Diseases 2007;45(6):770-8.R14XP04/2; R10XP04/2; QA - AGREE
Barham L, Lewis D, Latimer N. One to one interventions to reduce sexually transmitted infections and under the age of 18 conceptions: A systematic review of the economic evaluations. Sexually Transmitted Infections 83(6)()(pp 441-447), 2007 Date of Publication: Oct 2007 2007;(6):441-7.R05XP04/3; R05XP13/3 - ECONOMIC EVALUATION;
Ferrand RA, de SS, Cartledge JD. Tackling STI epidemics through the HIV clinic: is sex high enough on the agenda? International Journal of STD & AIDS 2008;19(10):711-2.R01XP04/3; 
Gahir S, Anger GJ, Ibrahim M, Read S, Piquette-Miller M. Management of hiv positive pregnancies in ontario: Current status. Canadian Journal of Clinical Pharmacology 2009;16(1):e68-e77.R02XP04/3; 
Ghitza UE, Epstein DH, Preston KL. Contingency management reduces injection-related HIV risk behaviors in heroin and cocaine using outpatients. Addictive Behaviors 2008;33(4):593-604.R10XP04/1; 
Griffiths C, Miles K, Aldam D, Cornforth D, Minton J. A nurse- and pharmacist-led treatment advice clinic for patients attending an HIV outpatient clinic. Journal of Advanced Nursing 2007;58(4):330-25.R14XP04/2; 
Handy P, Pattman RS, Richards J. 'I'm OK?' Evaluation of a new walk-in quick-check clinic.[see comment]. International Journal of STD & AIDS 2006;17(10):677-80.R16XP04/3; 
Harris ZK. Efficient allocation of resources to prevent HIV infection among injection drug users: the Prevention Point Philadelphia (PPP) needle exchange programme. Health Economics 2006;15(2):147-58.R10XP04/1; 
Hogben M, McNally T, McPheeters M, Hutchinson AB. The effectiveness of HIV partner counseling and referral services in increasing identification of HIV-positive individuals a systematic review.[see comment]. [Review] [28 refs]. American Journal of Preventive Medicine 2007;33(2 Suppl):S89-100.R14XP04/3; 
Javanbakht M, Prosser P, Grimes T, Weinstein M, Farthing C. Efficacy of an individualized adherence support program with contingent reinforcement among nonadherent HIV-positive patients: results from a randomized trial. Journal of the International Association of Physicians in AIDS Care: JIAPAC 2006;5(4):143-50.R14XP04/2; 
Khoo SH, Lloyd J, Dalton M, Bonington A, Hart E, Gibbons S et al. Pharmacologic optimization of protease inhibitors and nonnucleoside reverse transcriptase inhibitors (POPIN)--a randomized controlled trial of therapeutic drug monitoring and adherence support.  Journal of Acquired Immune Deficiency Syndromes: JAIDS 2006;41(4):461-7.R14XP04/2; 
Lin JS, Whitlock E, O'Connor E, Bauer V. Behavioral counseling to prevent sexually transmitted infections: A systematic review for the U.S. Preventive Services Task Force. Annals of Internal Medicine 149(7)()(pp 497-508), 2008 Date of Publication: 07 Oct 2008 2008;(7):497-508.R16XP04/3; 
Lucas GM, Mullen BA, Weidle PJ, Hader S, McCaul ME, Moore RD. Directly administered antiretroviral therapy in methadone clinics is associated with improved HIV treatment outcomes, compared with outcomes among concurrent comparison groups.[see comment]. Clinical Infectious Diseases 2006;42(11):1628-35.R14XP04/2; R10XP04/2; QA - AGREE
Lyons MS, Raab DL, Lindsell CJ, Trott AT, Fichtenbaum CJ. A novel emergency department based prevention intervention program for people living with HIV: evaluation of early experiences. BMC Health Services Research 2007;7:164.R14XP04/2; 
Mannheimer SB, Morse E, Matts JP, Andrews L, Child C, Schmetter B et al. Sustained benefit from a long-term antiretroviral adherence intervention. Results of a large randomized clinical trial. Journal of Acquired Immune Deficiency Syndromes: JAIDS 2006;43 Suppl 1:S41-S47.R14XP04/2; 
Masson CL, Sorensen JL, Perlman DC, Shopshire MS, Delucchi KL, Chen T et al. Hospital- versus community-based syringe exchange: A randomized controlled trial. AIDS Education and Prevention 19(2)()(pp 97-110), 2007 Date of Publication: Apr 2007 2007;(2):97-110.R10XP04/3; 
Metsch LR, Pereyra M, Messinger S, del RC, Strathdee SA, nderson-Mahoney P et al. HIV transmission risk behaviors among HIV-infected persons who are successfully linked to care. Clinical Infectious Diseases 2008;47(4):577-84.R14XP04/2; 
Naar-King S, Green M, Wright K, Outlaw A, Wang B, Liu H. Ancillary services and retention of youth in HIV care. AIDS Care 2007;19(2):248-51.R14XP04/2; 
Patel S, Weiss E, Chhabra R, Ryniker L, Adsuar R, Carness J et al. The Events in Care Screening Questionnaire (ECSQ): a new tool to identify needs and concerns of people with HIV/AIDS. AIDS Patient Care & Stds 2008;22(5):381-93.R14XP04/2; 
Reynolds NR, Testa MA, Su M, Chesney MA, Neidig JL, Frank I et al. Telephone support to improve antiretroviral medication adherence: a multisite, randomized controlled trial. Journal of Acquired Immune Deficiency Syndromes: JAIDS 2008;47(1):62-8.R14XP04/2; 
Rueda S, Park-Wyllie LY, Bayoumi AM, Tynan AM, Antoniou TA, Rourke SB et al. Patient support and education for promoting adherence to highly active antiretroviral therapy for HIV/AIDS.[update of Cochrane Database Syst Rev. 2000;(3):CD001442; PMID: 10908497]. [Review] [141 refs]. Cochrane Database of Systematic Reviews 2006;3:CD001442.R14XP04/2; 
Sinclair AH, Tolsma D, Weathersby A, Park MM. Feasibility of conducting a large, randomized controlled trial for STD counseling in a managed care setting. Sexually Transmitted Diseases 2008;35(11):920-3.R05XP04/1; 
Sundaram V, Lazzeroni LC, Douglass LR, Sanders GD, Tempio P, Owens DK. A randomized trial of computer-based reminders and audit and feedback to improve HIV screening in a primary care setting. International Journal of STD & AIDS 2009;20(8):527-33.R16XP04/1; R16XP05/1;
Takizawa C, Cheng D, Samet J, Winter M, Larson MJ, Saitz R. Primary medical care and reductions in HIV risk behaviors in adults with addictions. Journal of Addictive Diseases 2007;26(3):17-25.R10XP04/1; 
Takizawa C, Cheng D, Samet J, Winter M, Karsib MJ. Primary medical care and reductions in HIV risk behaviours in adults with addictions. Journal of Addictive Diseases 2007;26(3):17-25.R10XP04/3; 
Tariq S, Edwards SG, Nalabanda A, Ward H, Allen E, Fenton K et al. Sexual health services for South Asians in London, UK: a case-control study. International Journal of STD & AIDS 2007;18(8):563-4.R04XP04/3; 
Ulett KB, Willig JH, Lin HY, Routman JS, Abroms S, Allison J et al. The therapeutic implications of timely linkage and early retention in HIV care. AIDS Patient Care & Stds 2009;23(1):41-9.R14XP04/2; 
Wohl AR, Garland WH, Valencia R, Squires K, Witt MD, Kovacs A et al. A randomized trial of directly administered antiretroviral therapy and adherence case management intervention.[see comment]. Clinical Infectious Diseases 2006;42(11):1619-27.R14XP04/2; 
Wood E, Kerr T, Hogg RS, Palepu A, Zhang R, Strathdee SA et al. Impact of HIV testing on uptake of HIV therapy among antiretroviral naive HIV-infected injection drug users. Drug & Alcohol Review 2006;25(5):451-4.R10XP04/2; 
Zetola NM, Bernstein K, Ahrens K, Marcus JL, Philip S, Nieri G et al. Using surveillance data to monitor entry into care of newly diagnosed HIV-infected persons: San Francisco, 2006-2007. BMC Public Health 2009;9:17.R14XP04/2; 
2 codes
Altice FL, Maru DS, Bruce RD, Springer SA, Friedland GH. Superiority of directly administered antiretroviral therapy over self-administered therapy among HIV-infected drug users: a prospective, randomized, controlled trial. Clinical Infectious Diseases 2007;45(6):770-8.R14XP04/2; R10XP04/2; QA - AGREE
Gilbert P, Ciccarone D, Gansky SA, Bangsberg DR, Clanon K, McPhee SJ et al. Interactive "Video Doctor" counseling reduces drug and sexual risk behaviors among HIV-positive patients in diverse outpatient settings. PLoS ONE [Electronic Resource] 2008;3(4):e1988.R14XP02/2; R14XP04/2;
Holzemer WL, Bakken S, Portillo CJ, Grimes R, Welch J, Wantland D et al. Testing a nurse-tailored HIV medication adherence intervention. Nursing Research 2006;55(3):189-97.R14XP08/2; R14XP04/2;
Lucas GM, Mullen BA, Weidle PJ, Hader S, McCaul ME, Moore RD. Directly administered antiretroviral therapy in methadone clinics is associated with improved HIV treatment outcomes, compared with outcomes among concurrent comparison groups.[see comment]. Clinical Infectious Diseases 2006;42(11):1628-35.R14XP04/2; R10XP04/2; QA - AGREE
Roberto AJ, Zimmerman RS, Carlyle KE, Abner EL. A computer-based approach to preventing pregnancy, STD, and HIV in rural adolescents. Journal of Health Communication 2007;12(1):53-76.R05XP02/1; R05XP04/1;
Testing / Screening (P05)
1 code
Akers AY, Bernstein L, Doyle J, Corbie-Smith G. Older women and HIV testing: examining the relationship between HIV testing history, age, and lifetime HIV risk behaviors. Sexually Transmitted Diseases 2008;35(4):420-3.R08XP05/3; 
Anaya HD, Hoang T, Golden JF, Goetz MB, Gifford A, Bowman C et al. Improving HIV screening and receipt of results by nurse-initiated streamlined counseling and rapid testing. Journal of General Internal Medicine 2008;23(6):800-7.R16XP05/1; 
Bernstein KT, Liu KL, Begier EM, Koblin B, Karpati A, Murrill C. Same-sex attraction disclosure to health care providers among New York City men who have sex with men: implications for HIV testing approaches. Archives of Internal Medicine 2008;168(13):1458-64.R01XP05/3; 
Brown E, Chi BH, Read JS, Taha TE, Sharma U, Hoffman IF et al. Determining an optimal testing strategy for infants at risk for mother-to-child transmission of HIV-1 during the late postnatal period. AIDS 2008;22(17):2341-6.R02XP05/2; 
Burke RC, GSepkowitz KA, Bernstein T, Karpati AM, Myers JE. Why don't physicians test for HIV?: a review of the US literature. AIDS 2007;21(12):1617-24.R16XP05/3; 
Calderon Y, Leider J, Hailpern S, Haughey M, Ghosh R, Lombardi P et al. A randomized control trial evaluating the educational effectiveness of a rapid HIV posttest counseling video. Sexually Transmitted Diseases 2009;36(4):207-10.R16XP05/1; 
Calderon Y, Haughey M, Bijur PE, Leider J, Moreno-Walton L, Torres S et al. An educational HIV pretest counseling video program for off-hours testing in the emergency department.[see comment]. Annals of Emergency Medicine 2006;48(1):21-7.R16XP05/1; 
Calderon Y, Haughey M, Leider J, Bijur PE, Gennis P, Bauman LJ. Increasing willingness to be tested for human immunodeficiency virus in the emergency department during off-hour tours: a randomized trial. Sexually Transmitted Diseases 2007;34(12):1025-9.R16XP05/1; 
Carey MP, Coury-Doniger P, Senn TE, Vanable PA, Urban MA. Improving HIV rapid testing rates among STD clinic patients: a randomized controlled trial. Health Psychology 2008;27(6):833-8.R16XP05/3; 
Centers for Disease Control and Prevention (CDC). Late HIV testing - 34 states, 1996-2005. MMWR - Morbidity & Mortality Weekly Report 2009;58(24):661-5.R16XP05/3; 
Cohan D, Gomez E, Greenberg M, Washington S, Charlebois ED. Patient perspectives with abbreviated versus standard pre-test HIV counseling in the prenatal setting: a randomized-controlled, non-inferiority trial. PLoS ONE [Electronic Resource] 2009;4(4):e5166.R02XP05/3; 
Debattista J, Bryson G, Roudenko N, Dwyer J, Kelly M, Hogan P et al. Pilot of non-invasive (oral fluid) testing for HIV within a clinical setting. Sexual Health 2007;4(2):105-9.R16XP05/3; 
Desai MM, Rosenheck RA, Desai RA. Prevalence and correlates of human immunodeficiency virus testing and posttest counseling among outpatients with serious mental illness. Journal of Nervous & Mental Disease 2007;195(9):776-80.R15XP05/3; 
Dilley JW, Woods WJ, Loeb L, Nelson K, Sheon N, Mullan J et al. Brief cognitive counseling with HIV testing to reduce sexual risk among men who have sex with men: results from a randomized controlled trial using paraprofessional counselors. Journal of Acquired Immune Deficiency Syndromes: JAIDS 2007;44(5):569-77.R01XP05/1; 
Dorval V, Ritchie K, Gruslin A. Screening HIV in pregnancy: a survey of prenatal care patients. Canadian Journal of Public Health 2007;Revue Canadienne de Sante Publique. 98(5):379-82.R02XP05/3; 
Earl A, Albarracin D. Nature, decay, and spiraling of the effects of fear-inducing arguments and HIV counseling and testing: a meta-analysis of the short- and long-term outcomes of HIV-prevention interventions.[erratum appears in Health Psychol. 2007 Nov;26(6):815-6]. Health Psychology 2007;26(4):496-506.R16XP05/3; R16XP02/3;
Fisher M, Pao D, Murphy G, Dean G, McElborough D, Homer G et al. Serological testing algorithm shows rising HIV incidence in a UK cohort of men who have sex with men: 10 Years application. AIDS 2007;21(17):2309-14.R01XP05/3; 
Forbes KM, Lomax N, Cunningham L, Hardie J, Noble H, Sarner L et al. Partner notification in pregnant women with HIV: findings from three inner-city clinics. HIV Medicine 2008;9(6):433-5.R02XP05/3; 
Gandhi NR, Skanderson M, Gordon KS, Concato J, Justice AC. Delayed presentation for human immunodeficiency virus (HIV) care among veterans: a problem of access or screening? Medical Care 2007;45(11):1105-9.R14XP05/2;  QA - AGREE
Giles ML, Garland SM, Lewin SR, Hellard ME. What are the barriers to offering HIV testing in an antenatal setting?: a national study of obstetricians. AIDS 2007;21(12):1601-6.R02XP05/3; 
Greenwald JL, Rich CA, Bessega S, Posner MA, Maeda JL, Skolnik PR. Evaluation of the Centers for Disease Control and Prevention's recommendations regarding routine testing for human immunodeficiency virus by an inpatient service: who are we missing?[see comment]. Mayo Clinic Proceedings 2006;81(4):452-8.R16XP05/3; 
Guenter D, Barbara AM, Shaul RZ, Yudin MH, Remis RS, King SM. Prenatal HIV testing: women's experiences of informed consent in Toronto, Ontario. Journal of Obstetrics & Gynaecology Canada: JOGC  2008;30(1):17-22.R02XP05/3; 
Guenter D, Greer J, Barbara A, Robinson G, Roberts J, Browne G. Rapid point-of-care HIV testing in community-based anonymous testing program: a valuable alternative to conventional testing. AIDS Patient Care & Stds 2008;22(3):195-204.R16XP05/3; 
Guy RJ, Prybylski D, Fairley CK, Hellard ME, Kaldor JM. Can data from HIV voluntary counselling and testing be used to assess the impact of public health interventions? A literature review. International Journal of STD & AIDS 2009;20(6):378-83.R16XP05/3; 
Haukoos JS, Hopkins E, Eliopoulos VT, Byyny RL, Laperriere KA, Mendoza MX et al. Development and implementation of a model to improve identification of patients infected with HIV using diagnostic rapid testing in the emergency department. Academic Emergency Medicine 2007;14(12):1149-57.R16XP05/3; 
Holtgrave DR. The president's fiscal year 2007 initiative for human immunodeficiency virus counseling and testing expansion in the United States: a scenario analysis of its coverage, impact, and cost-effectiveness (DARE structured abstract). Journal of Public Health Management and Practice 2007;13:239-43.R13XP05/3; R16XP05/3; QA - INITIALY CODED AS PROTOCOL ONLY IN SRs
Hullett CR. Using functional theory to promote HIV testing: the impact of value-expressive messages, uncertainty, and fear.  Health Communication 2006;20(1):57-67.R05XP05/3;  QUERY COPIED FROM SR TO COHORTS - QA of cohorts database - AGREE
Hutchinson AB, Branson BM, Kim A, Farnham PG. A meta-analysis of the effectiveness of alternative HIV counseling and testing methods to increase knowledge of HIV status. [Review] [30 refs]. AIDS 2006;20(12):1597-604.R16XP05/3; 
Jamieson DJ, Cohen MH, Maupin R, Nesheim S, Danner SP, Lampe MA et al. Rapid human immunodeficiency virus-1 testing on labor and delivery in 17 US hospitals: the MIRIAD experience. American Journal of Obstetrics & Gynecology 2007;197(3 Suppl):S72-S82.R02XP05/3; 
Jenkins TC, Gardner EM, Thrun MW, Cohn DL, Burman WJ. Risk-based human immunodeficiency virus (HIV) testing fails to detect the majority of HIV-infected persons in medical care Settings. Sexually Transmitted Diseases 2006;33(5):329-33.R16XP05/3; 
Kacanek D, Eldridge GD, Nealey Moore J, MacGowan RJ, Binson D. Young incarcerated men's perceptions of and experiences with HIV testing. American Journal of Public Health 2007;97(7):1209-15.R07XP05/3; 
Karon JM, Song R, Brookmeyer R, Kaplan EH, Hall HI. Estimating HIV incidence in the United States from HIV/AIDS surveillance data and biomarker HIV test results. Statistics in Medicine 2008;27(23):4617-33.R16XP05/3; 
Kinsler JJ, Cunningham WE, Davis C, Wong MD. Time trends in failure to return for HIV test results. Sexually Transmitted Diseases 2007;34(6):397-400.R15XP05/3; 
Lauby JL, Milnamow M. Where MSM have their first HIV test: differences by race, income, and sexual identity. American Journal of Mens Health 2009;3(1):50-9.R01XP05/3; 
Lyons MS, Lindsell CJ, DA RN, Rn DL, Trott AT, Fichtenbaum CJ. Contributions to early HIV diagnosis among patients linked to care vary by testing venue. BMC Public Health 2008;8:220.R16XP05/2;  QA - AGREE
Manavi K, Clutterbuck D, Mackay R, McMillan A, Scott G. A rapid method for identifying high-risk patients consenting for HIV testing: introducing The Edinburgh Risk Assessment Table for HIV testing. International Journal of STD & AIDS 2006;17(4):234-6.R16XP05/3; 
Matthews PC, Van den AK, Dawson S, McIntyre M. HIV testing for adult patients with Streptococcus pneumoniae bacteraemia. Clinical Medicine 2006;6(5):512-Oct.R16XP05/3 - PEOPLE WITH OTHER INFECTIONS; 
Mayben JK, Kramer JR, Kallen MA, Franzini L, Lairson DR, Giordano TP. Predictors of delayed HIV diagnosis in a recently diagnosed cohort. AIDS Patient Care & Stds 2007;21(3):195-204.R16XP05/2; 
Mehta SD, Hall J, Lyss SB, Skolnik PR, Pealer LN, Kharasch S. Adult and pediatric emergency department sexually transmitted disease and HIV screening: programmatic overview and outcomes. Academic Emergency Medicine 2007;14(3):250-8.R16XP05/3; 
Mehta SD, Ghanem KG, Rompalo AM, Erbelding EJ. HIV seroconversion among public sexually transmitted disease clinic patients: analysis of risks to facilitate early identification. Journal of Acquired Immune Deficiency Syndromes: JAIDS 2006;42(1):116-22.R16XP05/3; 
Merchant RC, Gee EM, Clark MA, Mayer KH, Seage GR, III, Degruttola VG. Comparison of patient comprehension of rapid HIV pre-test fundamentals by information delivery format in an emergency department setting. BMC Public Health 2007;7:238.R16XP05/1;  QA - AGREE (BM)
Merchant RC, Clark MA, Mayer KH, Seage Iii GR, Degruttola VG, Becker BM. Video as an effective method to deliver pretest information for rapid human immunodeficiency testing. Academic Emergency Medicine 2009;16(2):124-35.R16XP05/3; 
Mimiaga MJ, Goldhammer H, Belanoff C, Tetu AM, Mayer KH. Men who have sex with men: perceptions about sexual risk, HIV and sexually transmitted disease testing, and provider communication.[see comment]. Sexually Transmitted Diseases 2007;34(2):113-9.R01XP05/3; 
Morgan J, Mills G. Implementing the universal routine-offer antenatal HIV screening programme in New Zealand: results from the first year. New Zealand Medical Journal 2007;120(1265):U2798.R02XP05/3; 
Moyer LB, Brouwer KC, Brodine SK, Ramos R, Lozada R, Cruz MF et al. Barriers and missed opportunities to HIV testing among injection drug users in two Mexico--US border cities. Drug & Alcohol Review 2008;27(1):39-45.R10XP05/3; 
Nguyen TQ, Ford CA, Kaufman JS, Leone PA, Suchindran C, Miller WC. HIV testing among young adults in the United States: Associations with financial resources and geography. American Journal of Public Health 2006;96(6):1031-4.R05XP05/3; 
Owens DK, Sundaram V, Lazzeroni LC, Douglass LR, Tempio P, Holodniy M et al. HIV testing of at risk patients in a large integrated health care system. Journal of General Internal Medicine 2007;22(3):315-20.R16XP05/3; 
Pai NP, Tulsky JP, Cohan D, Colford J, Reingold AL. Erratum: Rapid point-of-care HIV testing in pregnant women: A systematic review and meta-analysis (Tropical Medicine and International Health (2007) 12, (162-173)). Tropical Medicine and International Health 12(7)()(pp 902), 2007 Date of Publication: Jul 2007 2007;(7):902.R02XP05/3; 
Passin WF, Kim AS, Hutchinson AB, Crepaz N, Herbst JH, Lyles CM et al. A systematic review of HIV partner counseling and referral services: client and provider attitudes, preferences, practices, and experiences. Sexually Transmitted Diseases 2006;33(5):320-8.R16XP05/3; 
Prestage G, Jin F, Zablotska IB, Imrie J, Grulich AE, Pitts M. Trends in HIV testing among homosexual and bisexual men in eastern Australian states. Sexual Health 2008;5(2):119-23.R01XP05/3;  QA AGREE
Priddy FH, Pilcher CD, Moore RH, Tambe P, Park MN, Fiscus SA et al. Detection of acute HIV infections in an urban HIV counseling and testing population in the United States. Journal of Acquired Immune Deficiency Syndromes 44(2)()(pp 196-202), 2007 Date of Publication: Feb 2007 2007;(2):196-202.R16XP05/1; 
Pugatch D, Anderson BJ, O'Connell JV, Elson LC, Stein MD. HIV and HCV testing for young drug users in Rhode Island. Journal of Adolescent Health 2006;38(3):302-4.R10XP05/3; 
Rasch V, Yambesi F, Massawe S. Post-abortion care and voluntary HIV counselling and testing--an example of integrating HIV prevention into reproductive health services. Tropical Medicine & International Health 2006;11(5):697-704.R08XP05/3; 
Roberts KJ, Grusky O, Swanson AN. Outcomes of blood and oral fluid rapid HIV testing: a literature review, 2000-2006. [Review] [45 refs]. AIDS Patient Care & Stds 2007;21(9):621-37.R16XP05/3;  QA - AGREE
Schreiber C, Sammel M, Hillier SL, Barnhart KT. A little bit pregnant: Modeling how the accurate detection of pregnancy can improve HIV prevention trials. American Journal of Epidemiology 2009;169(4):515-21.R02XP05/3; 
Shrestha RK, Clark HA, Sansom SL, Song B, Buckendahl H, Calhoun CB et al. Cost-effectiveness of finding new HIV diagnoses using rapid HIV testing in community-based organizations.  Public Health Reports 2008;123 Suppl 3:94-100.R16XP05/3; 
Stevens PE, Hildebrandt E. Life changing words: women's responses to being diagnosed with HIV infection.[see comment]. Advances in Nursing Science 2006;29(3):207-21.R08XP05/2; 
Stokes SHM, McMaster P, Ismail KMK. Acceptability of perinatal rapid point-of-care HIV testing in an area of low HIV prevalence in the UK. Archives of Disease in Childhood 2007;192(6):505-8.R02XP05/3;  QA- AGREE
Struik SS, Tudor-Williams G, Taylor GP, Portsmouth SD, Foster CJ, Walsh C et al. Infant HIV infection despite "universal" antenatal testing. Archives of Disease in Childhood 2008;93(1):59-61.R02XP05/3; 
Surah S, O'Shea S, Dunn H, Mitra R, Fitzgerald C, Ibrahim F et al. Utilization of HIV point-of-care testing clinics in general practice and genitourinary medicine services in south-east London. International Journal of STD & AIDS 2009;20(3):168-9.R16XP05/3; 
Templeton DJ. Sexually transmitted infection and blood-borne virus screening in juvenile correctional facilities: a review of the literature and recommendations for Australian centres. [Review] [46 refs]. Journal of Clinical Forensic Medicine 2006;13(1):30-6.R05XP05/3; R13XP05/3;
Tepper NK, Farr SL, Danner SP, Maupin R, Nesheim SR, Cohen MH et al. Rapid human immunodeficiency virus testing in obstetric outpatient settings: the MIRIAD study. American Journal of Obstetrics & Gynecology 2009;201(1):31-6.R02XP05/3;  QA - AGREE
Tolou-Shams M, Payne N, Houck C, Pugatch D, Beausoleil N, Brown LK et al. HIV testing among at-risk adolescents and young adults: a prospective analysis of a community sample. Journal of Adolescent Health 2007;41(6):586-93.R05XP05/1; 
Trelle S, Shang A, Nartey L, Cassell JA, Low N. Improved effectiveness of partner notification for patients with sexually transmitted infections: systematic review (DARE structured abstract). BMJ 2007;334:354.R16XP05/3; 
Vidanapathirana J, Randeniya M. Publication of responses to a Cochrane systematic review on mass media interventions for HIV testing [abstract]. XV Cochrane Colloquium; 2007 Oct 23 27; Sao Paulo, Brazil 2007;149-50.R16XP05/1; R16XP13/1 - DISSEMINATION;
Walensky RP, Arbelaez C, Reichmann WM, Walls RM, Katz JN, Block BL et al. Revising expectations from rapid HIV tests in the emergency department.[see comment][summary for patients in Ann Intern Med. 2008 Aug 5;149(3):I30; PMID: 18678839]. Annals of Internal Medicine 2008;149(3):153-60.R16XP05/1; 
Weiss ES, Cornwell EE, III, Wang T, Syin D, Millman EA, Pronovost PJ et al. Human immunodeficiency virus and hepatitis testing and prevalence among surgical patients in an urban university hospital. American Journal of Surgery 2007;193(1):55-60.R16XP05/3;  QA - AGREE
Yudin MH, Moravac C, Shah RR. Influence of an "opt-out" test strategy and patient factors on human immunodeficiency virus screening in pregnancy. Obstetrics and Gynecology 110(1)()(pp 81-86), 2007 Date of Publication: Jul 2007 2007;(1):81-6.R02XP05/3;  COPIED FROM RCT
Zetola NM, Grijalva CG, Gertler S, Hare CB, Kaplan B, Dowling T et al. Simplifying consent for HIV testing is associated with an increase in HIV testing and case detection in highest risk groups, San Francisco January 2003-June 2007. PLoS ONE [Electronic Resource] 2008;3(7):e2591.R16XP05/3; 
2 codes
Holtgrave DR. The president's fiscal year 2007 initiative for human immunodeficiency virus counseling and testing expansion in the United States: a scenario analysis of its coverage, impact, and cost-effectiveness (DARE structured abstract). Journal of Public Health Management and Practice 2007;13:239-43.R13XP05/3; R16XP05/3; QA - INITIALY CODED AS PROTOCOL ONLY IN SRs
Sundaram V, Lazzeroni LC, Douglass LR, Sanders GD, Tempio P, Owens DK. A randomized trial of computer-based reminders and audit and feedback to improve HIV screening in a primary care setting. International Journal of STD & AIDS 2009;20(8):527-33.R16XP04/1; R16XP05/1;
Templeton DJ. Sexually transmitted infection and blood-borne virus screening in juvenile correctional facilities: a review of the literature and recommendations for Australian centres. [Review] [46 refs].  Journal of Clinical Forensic Medicine 2006;13(1):30-6.R05XP05/3; R13XP05/3;
Vidanapathirana J, Abramson MJ, Forbes A, Fairley C. Mass media interventions for promoting HIV testing: Cochrane systematic review. [Review] [1 refs]. International Journal of Epidemiology 2006;35(2):233-4.R16XP01/3; R16XP05/3;

Sero-sorting the undiagnosed (P06)
1 code
Holtgrave DR, Pinkerton SD. Can increasing awareness of HIV seropositivity reduce infections by 50% in the United States? Journal of Acquired Immune Deficiency Syndromes 2007;44(3):360-3.R16XP06/3; 
Pinkerton SD, Holtgrave DR, Galletly CL. Infections prevented by increasing HIV serostatus awareness in the United States, 2001 to 2004. Journal of Acquired Immune Deficiency Syndromes 2008;47(3):354-7.R16XP06/3; 
Williamson LM, Hart GJ. HIV prevalence and undiagnosed infection among a community sample of gay men in Scotland. Journal of Acquired Immune Deficiency Syndromes: JAIDS 2007;45(2):224-30.R01XP06/3;  QA - AGREE
2 codes
Dodds JP, Johnson AM, Parry JV, Mercey DE. A tale of three cities: persisting high HIV prevalence, risk behaviour and undiagnosed infection in community samples of men who have sex with men.[erratum appears in Sex Transm Infect. 2007 Oct;83(6):500]. Sexually Transmitted Infections 2007;83(5):392-6.R01XP09/3; R01XP06/3;

Transmission and interaction with other infections (P07)
1 code
Arvold ND, Ngo-Giang-Huong N, McIntosh K, Suraseranivong V, Warachit B, Piyaworawong S et al. Maternal HIV-1 DNA load and mother-to-child transmission. AIDS Patient Care & Stds 2007;21(9):638-43.R02XP07/1; 
Atashili J, Poole C, Ndumbe PM, Adimora AA, Smith JS. Bacterial vaginosis and HIV acquisition: a meta-analysis of published studies. [Review] [52 refs]. AIDS 2008;22(12):1493-501.R08XP07/1; 
Attia S, Egger M, Muller M, Zwahlen M, Low N. Sexual transmission of HIV according to viral load and antiretroviral therapy: systematic review and meta-analysis.[comment]. AIDS 2009;23(11):1397-404.R14XP07/3; 
Aweeka FT, Rosenkranz SL, Segal Y, Coombs RW, Bardeguez A, Thevanayagam L et al. The impact of sex and contraceptive therapy on the plasma and intracellular pharmacokinetics of zidovudine. AIDS (London, England) 2006;20(14):1833-41.R09XP07/2; 
Baggaley RF, Boily MC, White RG, Alary M. Risk of HIV-1 transmission for parenteral exposure and blood transfusion: a systematic review and meta-analysis. [Review] [83 refs]. AIDS 2006;20(6):805-12.R16XP07/2; 
Baggaley RF, White RG, Boily MC. Systematic review of orogenital HIV-1 transmission probabilities. [Review] [29 refs]. International Journal of Epidemiology 2008;37(6):1255-65.R16XP07/3; 
Baggley RF, Boily MC, White RG, Alary M. Risk of HIV-1 transmission for parenteral exposure and blood transfusion: a systematic review and meta-analysis. AIDS 2006;20(6):805-12.R16XP07/1; 
Boily MC, Baggaley RF, Wang L, Masse B, White RG, Hayes RJ et al. Heterosexual risk of HIV-1 infection per sexual act: systematic review and meta-analysis of observational studies. [Review] [125 refs]. The Lancet Infectious Diseases 2009;9(2):118-29.R09XP07/3; 
Bollen LJ, Whitehead SJ, Mock PA, Leelawiwat W, Asavapiriyanont S, Chalermchockchareonkit A et al. Maternal herpes simplex virus type 2 coinfection increases the risk of perinatal HIV transmission: possibility to further decrease transmission? AIDS 2008;22(10):1169-76.R02XP07/2; 
Bonell C, Hickson F, Beaumont M, Weatherburn P. Sexually transmitted infections as risk factors for HIV infection among MSMs: systematic review. Sexually Transmitted Diseases 2008;35(2):209.R01XP07/3; 
Brenner BG, Roger M, Routy J-P, Moisi D, Ntemgwa M, Matte C et al. High rates of forward transmission events after acute/early HIV-1 infection. Journal of Infectious Diseases 2007;195(7):951-9.R14XP07/2; 
Brown EL, Wald A, Hughes JP, Morrow RA, Krantz E, Mayer K et al. High risk of human immunodeficiency virus in men who have sex with men with herpes simplex virus type 2 in the EXPLORE study. American Journal of Epidemiology 2006;164(8):733-41.R01XP07/1; 
Byakika-Tusiime J. Circumcision and HIV infection: assessment of causality. [Review] [50 refs]. AIDS & Behavior 2008;12(6):835-41.R07XP07/1; 
Casper C, Carrell D, Miller KG, Judson FD, Meier AS, Pauk JS et al. HIV serodiscordant sex partners and the prevalence of human herpesvirus 8 infection among HIV negative men who have sex with men: baseline data from the EXPLORE Study. Sexually Transmitted Infections 2006;82(3):229-35.R14XP07/2; 
Celum C, Wald A, Hughes J, Sanchez J, Reid S, any-Moretlwe S et al. Effect of aciclovir on HIV-1 acquisition in herpes simplex virus 2 seropositive women and men who have sex with men: a randomised, double-blind, placebo-controlled trial.[see comment]. Lancet 2008;371(9630):2109-19.R01XP07/1; R08XP07/1;
Chung MH, Kiarie JN, Richardson BA, Lehman DA, Overbaugh J, Njiri F et al. Independent effects of nevirapine prophylaxis and HIV-1 RNA suppression in breast milk on early perinatal HIV-1 transmission. Journal of Acquired Immune Deficiency Syndromes: JAIDS 2007;46(4):472-8.R02XP07/1; 
Crawford JM, Kippax SC, Mao L, Van d, V, Prestage GP, Grulich AE et al. Number of risk acts by relationship status and partner serostatus: Findings from the HIM cohort of homosexually active men in Sydney, Australia. AIDS & Behavior 2006;10(3):325-31.R01XP07/3; 
Fox MP, Brooks DR, Kuhn L, Aldrovandi G, Sinkala M, Kankasa C et al. Role of breastfeeding cessation in mediating the relationship between maternal HIV disease stage and increased child mortality among HIV-exposed uninfected children. International Journal of Epidemiology 2009;38(2):569-76.R02XP07/1; 
Freeman EE, Weiss HA, Glynn JR, Cross PL, Whitworth JA, Hayes RJ. Herpes simplex virus 2 infection increases HIV acquisition in men and women: systematic review and meta-analysis of longitudinal studies. [Review] [56 refs]. AIDS 2006;20(1):73-83.R16XP07/1; 
Gilles MT, Dickinson JE, Cain A, Turner KA, McGuckin R, Loh R et al. Perinatal HIV transmission and pregnancy outcomes in indigenous women in Western Australia. Australian & New Zealand Journal of Obstetrics & Gynaecology 2007;47(5):362-7.R02XP07/3; 
Harris T, Panaro L, Phypers M, Choudhri Y, Archibald CP. HIV testing among Canadian tuberculosis cases from 1997 to 1998. Canadian Journal of Infectious Diseases and Medical Microbiology 2006;17(3):165-8.R16XP07/3; 
Helms DJ, Mosure DJ, Metcalf CA, Douglas JM, Jr., Malotte CK, Paul SM et al. Risk factors for prevalent and incident Trichomonas vaginalis among women attending three sexually transmitted disease clinics. Sexually Transmitted Diseases 2008;35(5):484-8.R08XP07/3; 
Horvath T, Madi BC, Iuppa IM, Kennedy GE, Rutherford G, Read JS. Interventions for preventing late postnatal mother-to-child transmission of HIV. [Review] [84 refs]. Cochrane Database of Systematic Reviews 2009;(1):CD006734.R02XP07/3; 
Humphrey JH, Iliff PJ, Marinda ET, Mutasa K, Moulton LH, Chidawanyika H et al. Effects of a single large dose of vitamin A, given during the postpartum period to HIV-positive women and their infants, on child HIV infection, HIV-free survival, and mortality. The Journal of infectious diseases 2006;193(6):860-71.R02XP07/2; 
Jin F, Prestage GP, Zablotska I, Rawstorne P, Kippax SC, Donovan B et al. High rates of sexually transmitted infections in HIV positive homosexual men: data from two community based cohorts. Sexually Transmitted Infections 2007;83(5):397-9.R14XP07/2; 
Jin F, Prestage GP, Ellard J, Kippax SC, Kaldor JM, Grulich AE. How homosexual men believe they became infected with HIV: the role of risk-reduction behaviors. Journal of Acquired Immune Deficiency Syndromes: JAIDS 2007;46(2):245-7.R01XP07/1; 
Jin F, Prestage GP, Mao L, Kippax SC, Pell CM, Donovan B et al. Incidence and risk factors for urethral and anal gonorrhoea and chlamydia in a cohort of HIV-negative homosexual men: the Health in Men Study. Sexually Transmitted Infections 2007;83(2):113-9.R01XP07/1; 
Kim JH, Psevdos G, Suh J, Sharp VL. Co-infection of hepatitis B and hepatitis C virus in human immunodeficiency virus-infected patients in New York City, United States. World Journal of Gastroenterology 2008;14(43):6689-93.R14XP07/2;  QA - AGREE
Koulinska IN, Villamor E, Chaplin B, Msamanga G, Fawzi W, Renjifo B et al. Transmission of cell-free and cell-associated HIV-1 through breast-feeding. Journal of acquired immune deficiency syndromes (1999) 2006;41(1):93-9.R02XP07/2; 
Larke NL, Weiss HA, Mayaud P, Van de PP, Clayton T, Ouedraogo A et al. Design of epidemiological studies measuring genital and plasma HIV-1 outcomes: lessons from a randomised controlled trial. Tropical medicine & international health : TM & IH 2009;14(3):267-75.R14XP07/2;  COPIED FROM RCT TO COHORTS
Lowe SH, Wensing AM, Droste JA, ten Kate RW, Jurriaans S, Burger DM et al. No virological failure in semen during properly suppressive antiretroviral therapy despite subtherapeutic local drug concentrations. HIV Clinical Trials 2006;7(6):285-90.R14XP07/2; 
Mason BW, Cartwright J, Sandham S, Whiteside C, Salmon RL. A patient notification exercise following infection control failures in a dental surgery.[see comment]. British Dental Journal 2008;205(4):E8-5.R16XP07/3;  QA - AGREE
Middleton MG, Grulich AE, McDonald AM, Donovan B, Hocking JS, Kaldor JM. Could sexually transmissible infections be contributing to the increase in HIV infections among men who have sex with men in Australia? Sexual Health 2008;5(2):131-40.R01XP07/3; 
Miller M, Liao Y, Gomez AM, Gaydos CA, D'Mellow D. Factors associated with the prevalence and incidence of Trichomonas vaginalis infection among African American women in New York city who use drugs.[see comment][comment]. Journal of Infectious Diseases 2008;197(4):503-9.R08XP07/3; R03XP07/3; QA - AGREE
Modjarrad K, Chamot E, Vermund SH. Impact of small reductions in plasma HIV RNA levels on the risk of heterosexual transmission and disease progression. [Review] [63 refs]. AIDS 2008;22(16):2179-85.R14XP07/2; 
O'Brien SF, Fan W, Ram SS, Goldman M, Nair RC, Chiavetta JA et al. Face-to-face interviewing in predonation screening: lack of effect on detected human immunodeficiency virus and hepatitis C virus infections.[erratum appears in Transfusion. 2006 Nov;46(11):2033]. Transfusion 2006;46(8):1380-7.R16XP07/2; 
Ouedraogo A, Nagot N, Vergne L, Konate I, Weiss HA, Defer MC et al. Impact of suppressive herpes therapy on genital HIV-1 RNA among women taking antiretroviral therapy: a randomized controlled trial. AIDS (London, England) 2006;20(18):2305-13.R14XP07/2;  QA - AGREE (BM)
Papenburg J, Blais D, Moore D, Al-Hosni M, Laferriere C, Tapiero B et al. Pediatric injuries from needles discarded in the community: epidemiology and risk of seroconversion. Pediatrics 2008;122(2):e487-e492.R05XP07/1; 
Peters BS, Jaoko W, Vardas E, Panayotakopoulos G, Fast P, Schmidt C et al. Studies of a prophylactic HIV-1 vaccine candidate based on modified vaccinia virus Ankara (MVA) with and without DNA priming: effects of dosage and route on safety and immunogenicity. Vaccine 2007;25(11):2120-7.R16XP07/1; 
Powers Ms PC, Pettifor AE, Cohen MS. Rethinking the heterosexual infectivity of HIV-1: a systematic review and meta-analysis. The Lancet Infectious Diseases 2008;8(9):553-63.R09XP07/3; 
Rieg G, Lewis RJ, Miller LG, Witt MD, Guerrero M, Daar ES. Asymptomatic sexually transmitted infections in HIV-infected men who have sex with men: Prevalence, incidence, predictors, and screening strategies. AIDS Patient Care and STDs 2008;22(12):947-54.R14XP07/2;  QUERY - why not MSM? COPIED FROM SR - r14 trumps
Rollins NC, Becquet R, Bland RM, Coutsoudis A, Coovadia HM. Infant feeding, HIV transmission and mortality at 18 months: the need for appropriate choices by mothers and prioritization within programmes. AIDS: An International Monthly Journal 2008;22(17):2349-57.R01XP07/3; 
Schramm DB, Meddows-Taylor S, Gray GE, Kuhn L, Tiemessen CT. Low maternal viral loads and reduced granulocyte-macrophage colony-stimulating factor levels characterize exposed, uninfected infants who develop protective human immunodeficiency virus type 1-specific responses. Clinical and vaccine immunology : CVI 2007;14(4):348-54.R02XP07/3; 
Schumann A, Nyamathi A, Stein JA. HIV risk reduction in a nurse case-managed TB and HIV intervention among homeless adults.  Journal of Health Psychology 2007;12(5):833-43.R13XP07/1; 
Thea DM, Aldrovandi G, Kankasa C, Kasonde P, Decker WD, Semrau K et al. Post-weaning breast milk HIV-1 viral load, blood prolactin levels and breast milk volume. AIDS 2006;20(11):1539-47.R02XP07/2; 
Traisathit P, Le CS, Mary JY, Kanjanasing A, Lamlertkittikul S, Lallemant M. Gestational age determination and prevention of HIV perinatal transmission. International Journal of Gynaecology & Obstetrics 2006;92(2):176-80.R02XP07/2; 
Villamor E, Aboud S, Koulinska IN, Kupka R, Urassa W, Chaplin B et al. Zinc supplementation to HIV-1-infected pregnant women: effects on maternal anthropometry, viral load, and early mother-to-child transmission. European Journal of Clinical Nutrition 2006;60(7):862-9.R02XP07/2; 
Wylie JL, Shah L, Jolly AM. Demographic, risk behaviour and personal network variables associated with prevalent hepatitis C, hepatitis B, and HIV infection in injection drug users in Winnipeg, Canada. BMC Public Health 2006;6:229.R10XP07/3;  QA - AGREE
Yao F, Seed C, Farrugia A, Morgan D, Cordner S, Wood D et al. The risk of HIV, HBV, HCV and HTLV infection among musculoskeletal tissue donors in Australia.[see comment]. American Journal of Transplantation 2007;7(12):2723-6.R16XP07/1; 
Zetola NM, Bernstein KT, Wong E, Louie B, Klausner JD. Exploring the relationship between sexually transmitted diseases and HIV acquisition by using different study designs. Journal of Acquired Immune Deficiency Syndromes: JAIDS 2009;50(5):546-51.R16XP07/3; 
Zuckerman RA, Lucchetti A, Whittington WL, Sanchez J, Coombs RW, Zuniga R et al. Herpes simplex virus (HSV) suppression with valacyclovir reduces rectal and blood plasma HIV-1 levels in HIV-1/HSV-2-seropositive men: a randomized, double-blind, placebo-controlled crossover trial. Journal of Infectious Diseases 2007;196(10):1500-8.R14XP07/2; 
2 codes
Carrico AW, Johnson MO, Moskowitz JT, Neilands TB, Morin SF, Charlebois ED et al. Affect regulation, stimulant use, and viral load among HIV-positive persons on anti-retroviral therapy. Psychosomatic Medicine 2007;69(8):785-92.R10XP02/2; R14XP07/2;
Celum C, Wald A, Hughes J, Sanchez J, Reid S, any-Moretlwe S et al. Effect of aciclovir on HIV-1 acquisition in herpes simplex virus 2 seropositive women and men who have sex with men: a randomised, double-blind, placebo-controlled trial.[see comment]. Lancet 2008;371(9630):2109-19.R01XP07/1; R08XP07/1;
Miller M, Liao Y, Gomez AM, Gaydos CA, D'Mellow D. Factors associated with the prevalence and incidence of Trichomonas vaginalis infection among African American women in New York city who use drugs.[see comment][comment]. Journal of Infectious Diseases 2008;197(4):503-9.R08XP07/3; R03XP07/3; QA - AGREE

Interventional Technologies (P08)
Abatemarco DJ, Catov JM, Cross H, Delnevo C, Hausman A. Factors associated with zidovudine receipt and prenatal care among HIV-infected pregnant women in New Jersey. Journal of Health Care for the Poor & Underserved 2008;19(3):814-28.R02XP08/3; 
Altice FL, Sullivan LE, Smith-Rohrberg D, Basu S, Stancliff S, Eldred L. The potential role of buprenorphine in the treatment of opioid dependence in HIV-infected individuals and in HIV infection prevention. [Review] [80 refs]. Clinical Infectious Diseases 2006;43 Suppl 4:S178-S183.R14XP08/2; 
Amico KR, Harman JJ, Johnson BT. Efficacy of antiretroviral therapy adherence interventions: a research synthesis of trials, 1996 to 2004. Journal of Acquired Immune Deficiency Syndromes: JAIDS 2006;41(3):285-97.R14XP08/2; 
Antoni MH, Carrico AW, Duran RE, Spitzer S, Penedo F, Ironson G et al. Randomized clinical trial of cognitive behavioral stress management on human immunodeficiency virus viral load in gay men treated with highly active antiretroviral therapy. Psychosomatic Medicine 2006;68(1):143-51.R14XP08/2;  QA - AGREE (BM)
Arrive E, Newell ML, Ekouevi DK, Chaix ML, Thiebaut R, Masquelier B et al. Prevalence of resistance to nevirapine in mothers and children after single-dose exposure to prevent vertical transmission of HIV-1: a meta-analysis. [Review] [44 refs]. International Journal of Epidemiology 2007;36(5):1009-21.R02XP08/3; 
Asboe D, Williams IG, Goodall RL, Darbyshire JH, Hooker MH, Babiker AG et al. A virological benefit from an induction/maintenance strategy: the Forte trial. Antiviral Therapy 2007;12(1):47-54.R14XP08/2; 
Baillargeon J, Giordano TP, Rich JD, Wu ZH, Wells K, Pollock BH et al. Accessing antiretroviral therapy following release from prison. JAMA 2009;301(8):848-57.R14XP08/2; 
Balfour L, Kowal J, Silverman A, Tasca GA, Angel JB, Macpherson PA et al. A randomized controlled psycho-education intervention trial: Improving psychological readiness for successful HIV medication adherence and reducing depression before initiating HAART. AIDS Care 2006;18(7):830-8.R14XP08/2; 
Bardeguez AD, Lindsey JC, Shannon M, Tuomala RE, Cohn SE, Smith E et al. Adherence to antiretrovirals among US women during and after pregnancy. Journal of Acquired Immune Deficiency Syndromes: JAIDS 2008;48(4):408-17.R02XP08/2; 
Barnhart K, Kulp JL, Rosen M, Shera DM. A randomized trial to determine the distribution of four topical gel formulations in the human vagina. Contraception 79(4)()(pp 297-303), 2009 Date of Publication: April 2009 2009;(4):297-303.R08XP08/1; 
Barnhart KT, Rosenberg MJ, Mackay HT, Blithe DL, Higgins J, Walsh T et al. Contraceptive efficacy of a novel spermicidal microbicide used with a diaphragm: a randomized controlled trial. Obstetrics and gynecology 2007;110(3):577-86.R08XP08/2; 
Beksinska M, Smit J, Mabude Z, Vijayakumar G, Linda J. Male partner involvement and assistance in female condom use. The European journal of contraception & reproductive health care : the official journal of the European Society of Contraception 2008;13(4):400-3.R07XP08/3; 
Blanchard E, Klibanov OM, Axelrod P, Palermo B, Samuel R. Virologic success in an urban HIV clinic: outcome at 12 months in patients who were HAART naive. HIV Clinical Trials 2008;9(3):186-91.R14XP08/2; 
Bleakley A, Fishbein M, Holtgrave D. An assessment of the relationship between condom labels and HIV-related beliefs and intentions. AIDS & Behavior 2008;12(3):452-8.R16XP08/1; 
Bloch MT, Smith DE, Quan D, Kaldor JM, Zaunders JJ, Petoumenos K et al. The role of hydroxyurea in enhancing the virologic control achieved through structured treatment interruption in primary HIV infection: final results from a randomized clinical trial (Pulse). Journal of Acquired Immune Deficiency Syndromes: JAIDS 2006;42(2):192-202.R14XP08/2; 
Bosch-Capblanch X, Abba K, Prictor M, Garner P. Contracts between patients and healthcare practitioners for improving patients' adherence to treatment, prevention and health promotion activities. Cochrane Database of Systematic Reviews: Reviews. Cochrane Database of Systematic Reviews 2007 Issue 2 Chichester (UK): John Wiley & Sons, Ltd 2007.
Bosch RJ, Bennett K, Collier AC, Zackin R, Benson CA. Pretreatment factors associated with 3-year (144-week) virologic and immunologic responses to potent antiretroviral therapy. Journal of acquired immune deficiency syndromes (1999) 2007;44(3):268-77.R14XP08/2; 
Boyle BA, Jayaweera D, Witt MD, Grimm K, Maa JF, Seekins DW. Randomization to once-daily stavudine extended release/lamivudine/efavirenz versus a more frequent regimen improves adherence while maintaining viral suppression. HIV Clinical Trials 2008;9(3):164-76.R14XP08/2; 
Braithwaite RS, Shechter S, Roberts MS, Schaefer A, Bangsberg DR, Harrigan PR et al. Explaining variability in the relationship between antiretroviral adherence and HIV mutation accumulation.[erratum appears in J Antimicrob Chemother. 2007 Apr;59(4):821]. Journal of Antimicrobial Chemotherapy 2006;58(5):1036-43.R14XP08/2; 
Broaddus MR, Bryan A. Consistent condom use among juvenile detainees: the role of individual differences, social bonding, and health beliefs. AIDS & Behavior 2008;12(1):59-67.R05XP08/1; R13XP08/1; QA - AGREE (BM)
Bruneau J, Daniel M, Kestens Y, Zang G, Genereux M. Associations between HIV-related injection behaviour and distance to and patterns of utilisation of syringe-supply programmes. Journal of Epidemiology & Community Health 2008;62(9):804-10.R10XP08/3; 
Bryant J, Baxter L, Hird S. Non-occupational postexposure prophylaxis for HIV: a systematic review. [Review] [31 refs]. Health Technology Assessment (Winchester, England) 2009;13(14):iii-iix.R16XP08/3; 
Buchbinder SP, Mehrotra DV, Duerr A, Fitzgerald DW, Mogg R, Li D et al. Efficacy assessment of a cell-mediated immunity HIV-1 vaccine (the Step Study): a double-blind, randomised, placebo-controlled, test-of-concept trial.[see comment]. Lancet 2008;372(9653):1881-93.R04XP08/1; 
Burman W, Grund B, Neuhaus J, Douglas J, Jr., Friedland G, Telzak E et al. Episodic antiretroviral therapy increases HIV transmission risk compared with continuous therapy: results of a randomized controlled trial. Journal of Acquired Immune Deficiency Syndromes: JAIDS 2008;49(2):142-50.R14XP08/2; 
Cai T, Gilbert PB, Self SG. Joint inferences on vaccine efficacy against infection and disease with application to the first HIV vaccine efficacy trial. Journal of Biopharmaceutical Statistics 2006;16(4):517-38.R16XP08/1; 
Callon C, Wood E, Marsh D, Li K, Montaner J, Kerr T. Barriers and facilitators to methadone maintenance therapy use among illicit opiate injection drug users in Vancouver. Journal of Opioid Management 2006;2(1):35-41.R10XP08/3; 
Canadian Coordinating Office for Health Technology Assessment. Vaccines for HIV (DARE structured abstract). Ottawa: Canadian Coordinating Office for Health Technology Assessment (CCOHTA) 2006;4.R16XP08/1;  QA - WAS EXCLUDED AS PROTOCOL ONLY - UPDATED 20.01.10
Carballo-Diéguez A, Dolezal C, Bauermeister JA, O'Brien W, Ventuneac A, Mayer K. Preference for gel over suppository as delivery vehicle for a rectal microbicide: results of a randomised, crossover acceptability trial among men who have sex with men. Sexually Transmitted Infections 2008;84(6):483-7.R01XP08/1; 
Cartier JJ, Greenwell L, Prendergast ML. The persistence of HIV risk behaviors among methamphetamine-using offenders. Journal of Psychoactive Drugs 2008;40(4):437-46.R10XP08/3; 
Chen MY, Millwood IY, Wand H, Poynten M, Law M, Kaldor JM et al. A randomized controlled trial of the safety of candidate microbicide SPL7013 gel when applied to the penis. Journal of Acquired Immune Deficiency Syndromes: JAIDS 2009;50(4):375-80.R07XP08/1;  QA - QUERY P01? Education? - No, original code correct
Chi BH, Chintu N, Cantrell RA, Kankasa C, Kruse G, Mbewe F et al. Addition of single-dose tenofovir and emtricitabine to intrapartum nevirapine to reduce perinatal HIV transmission. Journal of Acquired Immune Deficiency Syndromes: JAIDS 2008;48(2):220-3.R02XP08/2; 
Chiou PY, Kuo BI, Lee MB, Chen YM, Chuang P, Lin LC. A programme of symptom management for improving quality of life and drug adherence in AIDS/HIV patients. Journal of Advanced Nursing 2006;55(2):169-79.R14XP08/2; 
Choi K-H, Hoff C, Gregorich SE, Grinstead O, Gomez C, Hussey W. The efficacy of female condom skills training in HIV risk reduction among women: A randomized controlled trial. American Journal of Public Health 98(10)()(pp 1841-1848), 2008 Date of Publication: 01 Oct 2008 2008;(10):1841-8.R08XP08/1; 
Coffin PO, Latka MH, Latkin C, Wu Y, Purcell DW, Metsch L et al. Safe syringe disposal is related to safe syringe access among HIV-positive injection drug users. AIDS & Behavior 2007;11(5):652-62.R14XP08/2; R10XP08/2;
Crosby R, DiClemente RJ, Yarber WL, Snow G, Troutman A. An event-specific analysis of condom breakage among African American men at risk of HIV acquisition. Sexually Transmitted Diseases 2008;35(2):174-7.R03XP08/1; R07XP08/1;
Cutler B, Justman J. Vaginal microbicides and the prevention of HIV transmission. The Lancet Infectious Diseases 2008;8(11):685-97.R07XP08/3; 
de MC, Garrido C, Corral A, Ramirez-Olivencia G, Jimenez-Nacher I, Zahonero N et al. Changing rates and patterns of drug resistance mutations in antiretroviral-experienced HIV-infected patients. AIDS Research & Human Retroviruses 2007;23(7):879-85.R14XP08/2; 
DeJesus E, Ruane P, McDonald C, Garcia F, Sharma S, Corales R et al. Impact of switching virologically suppressed, HIV-1-infected patients from twice-daily fixed-dose zidovudine/lamivudine to once-daily fixed-dose tenofovir disoproxil fumarate/emtricitabine. HIV Clinical Trials 2008;9(2):103-14.R14XP08/2; 
DeJesus E, Young B, Morales-Ramirez JO, Sloan L, Ward DJ, Flaherty JF et al. Simplification of antiretroviral therapy to a single-tablet regimen consisting of efavirenz, emtricitabine, and tenofovir disoproxil fumarate versus unmodified antiretroviral therapy in virologically suppressed HIV-1-infected patients. Journal of Acquired Immune Deficiency Syndromes: JAIDS 2009;51(2):163-74.R14XP08/2; 
Desai K, Sansom SL, Ackers ML, Stewart SR, Hall HI. Modeling the impact of HIV chemoprophylaxis strategies among men who have sex with men in the United States: HIV infections prevented and cost-effectiveness. AIDS - An International Monthly Journal 2008;22(14):1829-39.R01XP08/1; 
Di GS, Bracciale L, Colafigli M, Cattani P, Pinnetti C, Bacarelli A et al. Declining prevalence of HIV-1 drug resistance in treatment-failing patients: a clinical cohort study.[erratum appears in Antivir Ther. 2007;12(7):1145 Note: Colatigli, Manuela [corrected to Colafigli, Manuela; Cattani, Paola [added]; Pannetti, Carmen [corrected to Pinnetti, Carmen]]. Antiviral Therapy 2007;12(5):835-9.R14XP08/2; 
DiIorio C, McCarty F, Resnicow K, McDonnell HM, Soet J, Yeager K et al. Using motivational interviewing to promote adherence to antiretroviral medications: a randomized controlled study. AIDS Care 2008;20(3):273-83.R14XP08/2; 
Doxanakis A, Read T, Levy R, Mijch A, Fairley CK. Cohort analysis of two multidisciplined adherence intervention programmes for patients on antiretroviral therapy. International Journal of STD & AIDS 2006;17(4):257-9.R14XP08/2; 
Dunn D, Geretti AM, Green H, Fearnhill E, Pozniak A, Churchill D et al. Population trends in the prevalence and patterns of protease resistance related to exposure to unboosted and boosted protease inhibitors. Antiviral Therapy 2008;13(6):771-7.R14XP08/2; 
Edwards LV. Perceived social support and HIV/AIDS medication adherence among African American women. Qualitative Health Research 2006;16(5):679-91.R14XP08/2; 
El-Sadr WM, Mayer KH, Maslankowski L, Hoesley C, Justman J, Gai F et al. Safety and acceptability of cellulose sulfate as a vaginal microbicide in HIV-infected women. AIDS 2006;20(8):1109-16.R14XP08/2; 
Elder A, Paterson C. Sharps injuries in UK health care: a review of injury rates, viral transmission and potential efficacy of safety devices. [Review] [83 refs]. Occupational Medicine (Oxford)  2006;56(8):566-74.R16XP08/3 - HEALTH WORKERS; 
Ena J, Ruiz de Apodaca RF, Amador C, Benito C, Pasquau F. Net benefits of resistance testing directed therapy compared with standard of care in HIV-infected patients with virological failure: A meta-analysis.[see comment]. Enfermedades Infecciosas y Microbiologia Clinica 2006;24(4):232-7.R14XP08/2; 
Eshleman SH, Husnik M, Hudelson S, Donnell D, Huang Y, Huang W et al. Antiretroviral drug resistance, HIV-1 tropism, and HIV-1 subtype among men who have sex with men with recent HIV-1 infection. AIDS 2007;21(9):1165-74.R14XP08/2; 
Fajman N, Wright R. Use of antiretroviral HIV post-exposure prophylaxis in sexually abused children and adolescents treated in an inner-city pediatric emergency department. Child Abuse & Neglect 2006;30(8):919-27.R05XP08/1; 
Fidler S, Fraser C, Fox J, Tamm N, Griffin JT, Weber J. Comparative potency of three antiretroviral therapy regimes in primary HIV infection. AIDS 2006;20(2):247-52.R14XP08/2; 
Fielden SJ, Rusch ML, Yip B, Wood E, Shannon K, Levy AR et al. Nonadherence increases the risk of hospitalization among HIV-infected antiretroviral naive patients started on HAART. Journal of the International Association of Physicians in AIDS Care: JIAPAC 2008;7(5):238-44.R14XP08/2; 
Fischl MA, Collier AC, Mukherjee AL, Feinberg JE, Demeter LM, Tebas P et al. Randomized open-label trial of two simplified, class-sparing regimens following a first suppressive three or four-drug regimen. AIDS 2007;21(3):325-33.R14XP08/2; 
Forthal DN, Gilbert PB, Landucci G, Phan T. Recombinant gp120 vaccine-induced antibodies inhibit clinical strains of HIV-1 in the presence of Fc receptor-bearing effector cells and correlate inversely with HIV infection rate. Journal of Immunology 2007;178(10):6596-603.R16XP08/1; 
Frick P, Tapia K, Grant P, Novotny M, Kerzee J. The effect of a multidisciplinary program on HAART adherence. AIDS Patient Care & Stds 2006;20(7):511-24.R14XP08/2; 
Fuchs EJ, Lee LA, Torbenson MS, Parsons TL, Bakshi RP, Guidos AM et al. Hyperosmolar sexual lubricant causes epithelial damage in the distal colon: potential implication for HIV transmission. The Journal of infectious diseases 2007;195(5):703-10.R16XP08/1; 
Gardner EM, Sharma S, Peng G, Hullsiek KH, Burman WJ, Macarthur RD et al. Differential adherence to combination antiretroviral therapy is associated with virological failure with resistance. AIDS 2008;22(1):75-82.R14XP08/2; 
Garland WH, Wohl AR, Valencia R, Witt MD, Squires K, Kovacs A et al. The acceptability of a directly-administered antiretroviral therapy (DAART) intervention among patients in public HIV clinics in Los Angeles, California. AIDS Care 2007;19(2):159-67.R14XP08/2; 
Giles ML, McDonald AM, Elliott EJ, Ziegler JB, Hellard ME, Lewin SR et al. Variable uptake of recommended interventions to reduce mother-to-child transmission of HIV in Australia, 1982-2005. Medical Journal of Australia 2008;189(3):151-4.R02XP08/3; 
Girardet RG, Lemme S, Biason TA, Bolton K, Lahoti S. HIV post-exposure prophylaxis in children and adolescents presenting for reported sexual assault. Child Abuse & Neglect 2009;33(3):173-8.R15XP08/1; R05XP08/1; QA - AGREE
Golin CE, Earp J, Tien HC, Stewart P, Porter C, Howie L. A 2-arm, randomized, controlled trial of a motivational interviewing-based intervention to improve adherence to antiretroviral therapy (ART) among patients failing or initiating ART. Journal of Acquired Immune Deficiency Syndromes: JAIDS 2006;42(1):42-51.R14XP08/2; 
Golub ET, Strathdee SA, Bailey SL, Hagan H, Latka MH, Hudson SM et al. Distributive syringe sharing among young adult injection drug users in five U.S. cities. Drug & Alcohol Dependence 2007;91 Suppl 1:S30-S38.R10XP08/1; R05XP08/1;
Gonzalez JS, Penedo FJ, Llabre MM, Duran RE, Antoni MH, Schneiderman N et al. Physical symptoms, beliefs about medications, negative mood, and long-term HIV medication adherence. Annals of Behavioral Medicine 2007;34(1):46-55.R14XP08/2; 
Goonetilleke N, Moore S, Dally L, Winstone N, Cebere I, Mahmoud A et al. Induction of multifunctional human immunodeficiency virus type 1 (HIV-1)-specific T cells capable of proliferation in healthy subjects by using a prime-boost regimen of DNA- and modified vaccinia virus Ankara-vectored vaccines expressing HIV-1 Gag coupled to CD8+ T-cell epitopes. Journal of Virology 2006;80(10):4717-28.R16XP08/1; 
Gordon CM. Commentary on meta-analysis of randomized controlled trials for HIV treatment adherence interventions. Research directions and implications for practice. Journal of Acquired Immune Deficiency Syndromes: JAIDS 2006;43 Suppl 1:S36-S40.R14XP08/2; 
Gorse GJ, Baden LR, Wecker M, Newman MJ, Ferrari G, Weinhold KJ et al. Safety and immunogenicity of cytotoxic T-lymphocyte poly-epitope, DNA plasmid (EP HIV-1090) vaccine in healthy, human immunodeficiency virus type 1 (HIV-1)-uninfected adults. Vaccine 2008;26(2):215-23.R16XP08/1; 
Gossec L, Tubach F, Dougados M, Ravaud P. Reporting of adherence to medication in recent randomized controlled trials of 6 chronic diseases: a systematic literature review. [Review] [29 refs]. American Journal of the Medical Sciences 2007;334(4):248-54.R14XP08/2; 
Gowing L, Farrell M, Bornemann R, Sullivan L, Ali R. Substitution treatment of injecting opioid users for prevention of HIV infection. Cochrane Database of Systematic Reviews 2008;(2).R10XP08/3; 
Gowing LR, Farrell M, Bornemann R, Sullivan LE, Ali RL. Brief report: Methadone treatment of injecting opioid users for prevention of HIV infection. [Review] [29 refs]. Journal of General Internal Medicine 2006;21(2):193-5.R10XP08/3; 
Graham BS, Koup RA, Roederer M, Bailer RT, Enama ME, Moodie Z et al. Phase 1 safety and immunogenicity evaluation of a multiclade HIV-1 DNA candidate vaccine.[see comment]. Journal of Infectious Diseases 2006;194(12):1650-60.R16XP08/1; 
Gross R, Yip B, Lo RV, III, Wood E, Alexander CS, Harrigan PR et al. A simple, dynamic measure of antiretroviral therapy adherence predicts failure to maintain HIV-1 suppression. Journal of Infectious Diseases 2006;194(8):1108-14.R14XP08/2; 
Grossman C, Hadley W, Brown LK, Houck CD, Peters A, Tolou-Shams M et al. Adolescent sexual risk: factors predicting condom use across the stages of change. AIDS & Behavior 2008;12(6):913-22.R05XP08/1; 
Guest G, Shattuck D, Johnson L, Akumatey B, Clarke EE, Chen PL et al. Changes in sexual risk behavior among participants in a PrEP HIV prevention trial.[see comment]. Sexually Transmitted Diseases 2008;35(12):1002-8.R16XP08/1; 
Hagan H, Campbell JV, Thiede H, Strathdee SA, Ouellet L, Latka M et al. Injecting alone among young adult IDUs in five US cities: evidence of low rates of injection risk behavior.  Drug & Alcohol Dependence 2007;91 Suppl 1:S48-S55.R10XP08/1; R05XP08/1;
Herrmann S, McKinnon E, John M, Hyland N, Martinez OP, Cain A et al. Evidence-based, multifactorial approach to addressing non-adherence to antiretroviral therapy and improving standards of care. Internal Medicine Journal 2008;38(1):8-15.R14XP08/2; 
Hirsch JD, Rosenquist A, Best BM, Miller TA, Gilmer TP. Evaluation of the first year of a pilot program in community pharmacy: HIV/AIDS medication therapy management for Medi-Cal beneficiaries. Journal of Managed Care Pharmacy 2009;15(1):32-41.R14XP08/2; 
Holzemer WL, Bakken S, Portillo CJ, Grimes R, Welch J, Wantland D et al. Testing a nurse-tailored HIV medication adherence intervention. Nursing Research 2006;55(3):189-97.R14XP08/2; R14XP04/2;
Horne R, Cooper V, Gellaitry G, Date HL, Fisher M. Patients' perceptions of highly active antiretroviral therapy in relation to treatment uptake and adherence: the utility of the necessity-concerns framework. Journal of Acquired Immune Deficiency Syndromes: JAIDS 2007;45(3):334-41.R14XP08/2; 
Huo D, Bailey SL, Ouellet LJ. Cessation of injection drug use and change in injection frequency: The Chicago needle exchange evaluation study. Addiction 2006;101(11):1606-13.R10XP08/3; 
Huo D, Ouellet LJ. Needle exchange and injection-related risk behaviors in Chicago: A longitudinal study. Journal of Acquired Immune Deficiency Syndromes 2007;45(1):108-14.R10XP08/3; 
Huo D, Ouellet LJ. Needle exchange and sexual risk behaviors among a cohort of injection drug users in Chicago, Illinois. Sexually Transmitted Diseases 2009;36(1):35-40.R10XP08/1; 
Hurwitz BE, Klaus JR, Llabre MM, Gonzalez A, Lawrence PJ, Maher KJ et al. Suppression of human immunodeficiency virus type 1 viral load with selenium supplementation: a randomized controlled trial.[see comment]. Archives of Internal Medicine 2007;167(2):148-54.R14XP08/2; 
Ingersoll KS, Cohen J. The impact of medication regimen factors on adherence to chronic treatment: a review of literature. [Review] [72 refs]. Journal of Behavioral Medicine 2008;31(3):213-24.R14XP08/2;  QA - Disagree - but correct - QUERY - is the risk group specified? Again, I'm not sure how R14 works so might be my ignorance rather than query!
Jackson JB, Parsons T, Musoke P, Nakabiito C, Donnell D, Fleming T et al. Association of cord blood nevirapine concentration with reported timing of dose and HIV-1 transmission.[see comment]. AIDS 2006;20(2):217-22.R02XP08/3;  QA - AGREE (BM)
Jacobson JM, Pat BR, Spritzler J, Saag MS, Eron JJ, Jr., Coombs RW et al. Evidence that intermittent structured treatment interruption, but not immunization with ALVAC-HIV vCP1452, promotes host control of HIV replication: the results of AIDS Clinical Trials Group 5068. Journal of Infectious Diseases 2006;194(5):623-32.R14XP08/2; 
Jia H, Uphold CR, Zheng Y, Wu S, Chen GJ, Findley K et al. A further investigation of health-related quality of life over time among men with HIV infection in the HAART era. Quality of Life Research 2007;16(6):961-8.R14XP08/2; 
Johnson MO, Charlebois E, Morin SF, Remien RH, Chesney MA, National Institute of Mental Health Healthy Living Project Team. Effects of a behavioral intervention on antiretroviral medication adherence among people living with HIV: the healthy living project randomized controlled study. Journal of Acquired Immune Deficiency Syndromes: JAIDS 2007;46(5):574-80.R14XP08/2; 
Jones DL, Pherson-Baker S, Lydston D, Camille J, Brondolo E, Tobin JN et al. Efficacy of a group medication adherence intervention among HIV positive women: the SMART/EST Women's Project. AIDS & Behavior 2007;11(1):79-86.R14XP08/2; 
Kapadia F, Latka MH, Hudson SM, Golub ET, Campbell JV, Bailey S et al. Correlates of consistent condom use with main partners by partnership patterns among young adult male injection drug users from five US cities. Drug and Alcohol Dependence 2007;91 Suppl 1:S56-S63.R10XP08/3; R05XP08/3; R07XP08/3; COPIED FROM RCT TO COHORTS
Kapadia F, Vlahov D, Wu Y, Cohen MH, Greenblatt RM, Howard AA et al. Impact of drug abuse treatment modalities on adherence to ART/HAART among a cohort of HIV seropositive women. American Journal of Drug & Alcohol Abuse 2008;34(2):161-70.R14XP08/2; 
Kelleher AD, Puls RL, Bebbington M, Boyle D, Ffrench R, Kent SJ et al. A randomized, placebo-controlled phase I trial of DNA prime, recombinant fowlpox virus boost prophylactic vaccine for HIV-1. AIDS 2006;20(2):294-7.R16XP08/1; 
Keller MJ, Guzman E, Hazrati E, Kasowitz A, Cheshenko N, Wallenstein S et al. PRO 2000 elicits a decline in genital tract immune mediators without compromising intrinsic antimicrobial activity. AIDS (London, England) 2007;21(4):467-76.R08XP08/1; 
Keller MJ, Zerhouni-Layachi B, Cheshenko N, John M, Hogarty K, Kasowitz A et al. PRO 2000 gel inhibits HIV and herpes simplex virus infection following vaginal application: A double-blind placebo-controlled trial. Journal of Infectious Diseases 193(1)()(pp 27-35), 2006 Date of Publication: 01 Jan 2006 2006;(1):27-35.R14XP08/2; 
Khalsa J, Vocci F, Altice F, Fiellin D, Miller V. Buprenorphine and HIV primary care: new opportunities for integrated treatment. [Review] [15 refs]. Clinical Infectious Diseases 2006;43 Suppl 4:S169-S172.R14XP08/3; 
Khanna N, Dalby R, Connor A, Church A, Stern J, Frazer N. Phase I clinical trial of repeat dose terameprocol vaginal ointment in healthy female volunteers. Sexually Transmitted Diseases 2008;35(6):577-82.R08XP08/1; 
Koenig LJ, Pals SL, Bush T, Pratt PM, Stratford D, Ellerbrock TV. Randomized controlled trial of an intervention to prevent adherence failure among HIV-infected patients initiating antiretroviral therapy. Health Psychology 2008;27(2):159-69.R14XP08/2; 
Kompoti M, Koutsovassilis A, Skrapari IC. Antiretroviral resistance testing in treatment-naive HIV-infected adults. Cochrane Database of Systematic Reviews: Protocols. Cochrane Database of Systematic Reviews 2007 Issue 2 Chichester (UK): John Wiley & Sons, Ltd 2007.
Kozal MJ, Hullsiek KH, Macarthur RD, Berg-Wolf M, Peng G, Xiang Y et al. The Incidence of HIV drug resistance and its impact on progression of HIV disease among antiretroviral-naive participants started on three different antiretroviral therapy strategies. HIV Clinical Trials 2007;8(6):357-70.R14XP08/2; 
Larney S, Dolan K. A literature review of international implementation of opioid substitution treatment in prisons: equivalence of care?. [Review] [52 refs]. European Addiction Research 2009;15(2):107-12.R10XP08/3; R13XP08/3;
Leonard L, DeRubeis E, Pelude L, Medd E, Birkett N, Seto J. "I inject less as I have easier access to pipes": injecting, and sharing of crack-smoking materials, decline as safer crack-smoking resources are distributed. International Journal of Drug Policy 2008;19(3):255-64.R10XP08/3; 
Levin TR, Klibanov OM, Axelrod P, van dB-W, Finley GL, Gray A et al. A randomized trial of educational materials, pillboxes, and mailings to improve adherence with antiretroviral therapy in an Inner City HIV clinic. Journal of Clinical Outcomes Management 13(4)()(pp 217-221), 2006 Date of Publication: Apr 2006 2006;(4):217-21.R14XP08/2; 
Li X, Chu H, Gallant JE, Hoover DR, Mack WJ, Chmiel JS et al. Bimodal virological response to antiretroviral therapy for HIV infection: an application using a mixture model with left censoring. Journal of Epidemiology & Community Health 2006;60(9):811-8.R14XP08/2; 
Lima V, Fernandes K, Rachlis B, Druyts E, Montaner J, Hogg R. Migration adversely affects antiretroviral adherence in a population-based cohort of HIV/AIDS patients. Social Science & Medicine 2009;68(6):1044-9.R14XP08/2; 
Llabre MM, Weaver KE, Duran RE, Antoni MH, Pherson-Baker S, Schneiderman N. A measurement model of medication adherence to highly active antiretroviral therapy and its relation to viral load in HIV-positive adults. AIDS Patient Care & Stds 2006;20(10):701-11.R14XP08/2; 
Lott DC, Strain EC, Brooner RK, Bigelow GE, Johnson RE. HIV risk behaviors during pharmacologic treatment for opioid dependence: a comparison of levomethadyl acetate [corrected] buprenorphine, and methadone.[erratum appears in J Subst Abuse Treat. 2006 Oct;31(3):317]. Journal of Substance Abuse Treatment 2006;31(2):187-94.R10XP08/3; 
Loutfy MR, Macdonald S, Myhr T, Husson H, Du MJ, Balla S et al. Prospective cohort study of HIV post-exposure prophylaxis for sexual assault survivors. Antiviral Therapy 2008;13(1):87-95.R15XP08/1; 
Ma M, Brown BR, Coleman M, Kibler JL, Loewenthal H, Mitty JA. The feasibility of modified directly observed therapy for HIV-seropositive African American substance users. AIDS Patient Care & Stds 2008;22(2):139-46.R14XP08/2; 
Macalino GE, Hogan JW, Mitty JA, Bazerman LB, DeLong AK, Loewenthal H et al. A randomized clinical trial of community-based directly observed therapy as an adherence intervention for HAART among substance users. AIDS 2007;21(11):1473-7.R14XP08/2; R10XP08/2;
Madi BC, Smith N, Leroy V, Horvath TH, Kennedy GE, Dabis F et al. Interventions for preventing postnatal mother-to-child transmission of HIV. Cochrane Database of Systematic Reviews (3), 2007 Article Number: CD006734 Date of Publication: 2007 2007;(3).R02XP08/3; 
Maitland D, Jackson A, Osorio J, Mandalia S, Gazzard BG, Moyle GJ et al. Switching from twice-daily abacavir and lamivudine to the once-daily fixed-dose combination tablet of abacavir and lamivudine improves patient adherence and satisfaction with therapy. HIV Medicine 2008;9(8):667-72.R14XP08/2; 
Malta M, Strathdee SA, Magnanini MM, Bastos FI. Adherence to antiretroviral therapy for human immunodeficiency virus/acquired immune deficiency syndrome among drug users: a systematic review. [Review] [66 refs]. Addiction 2008;103(8):1242-57.R14XP08/2; 
Mannheimer S, Thackeray L, Huppler HK, Chesney M, Gardner EM, Wu AW et al. A randomized comparison of two instruments for measuring self-reported antiretroviral adherence. AIDS Care 2008;20(2):161-9.R14XP08/2; 
Mansoor LE, Dowse R. Medicines information and adherence in HIV/AIDS patients. Journal of Clinical Pharmacy & Therapeutics 2006;31(1):7-15.R14XP08/2; 
Mantell JE, Morar NS, Myer L, Ramjee G. "We have our protector": misperceptions of protection against HIV among participants in a microbicide efficacy trial. American Journal of Public Health 2006;96(6):1073-7.R12XP08/1; R08XP08/1;
Marc LG, Testa MA, Walker AM, Robbins GK, Shafer RW, Anderson NB et al. Educational attainment and response to HAART during initial therapy for HIV-1 infection. Journal of Psychosomatic Research 2007;63(2):207-16.R14XP08/2; 
Marshall BD, Wood E, Zhang R, Tyndall MW, Montaner JS, Kerr T. Condom use among injection drug users accessing a supervised injecting facility. Sexually Transmitted Infections 2009;85(2):121-6.R10XP08/3; 
Maru DS, Bruce RD, Walton M, Mezger JA, Springer SA, Shield D et al. Initiation, adherence, and retention in a randomized controlled trial of directly administered antiretroviral therapy. AIDS & Behavior 2008;12(2):284-93.R14XP08/2; 
Maru DS, Bruce RD, Walton M, Springer SA, Altice FL. Persistence of virological benefits following directly administered antiretroviral therapy among drug users: results from a randomized controlled trial. Journal of Acquired Immune Deficiency Syndromes: JAIDS 2009;50(2):176-81.R14XP08/2; R14XP08/2;
McFarland EJ, Johnson DC, Muresan P, Fenton T, Tomaras GD, McNamara J et al. HIV-1 vaccine induced immune responses in newborns of HIV-1 infected mothers. AIDS 2006;20(11):1481-9.R02XP08/3; 
Meader N, Li R, Des JD, Pilling S. Psychosocial interventions for the reduction of injection and sexual risk behaviour for preventing HIV in drug users - PROTOCOL. Cochrane Database of Systematic Reviews (2), 2008 Article Number: CD007192 Date of Publication: 2008 2008;(2).R10XP08/1;  QA - WAS PROTOCOL ONLY - UPDATED 21.01.10
Merenstein D, Schneider MF, Cox C, Schwartz R, Weber K, Robison E et al. Association of child care burden and household composition with adherence to highly active antiretroviral therapy in the Women's Interagency HIV Study. AIDS Patient Care & Stds 2009;23(4):289-96.R14XP08/2; 
Millett GA, Flores SA, Marks G, Reed JB, Herbst JH. Circumcision status and risk of HIV and sexually transmitted infections among men who have sex with men: a meta-analysis.[see comment]. [Review] [66 refs]. JAMA 2008;300(14):1674-84.R01XP08/1;  QA - AGREE
Millett GA, Flores SA, Marks G, Bailey Reed J, Herbst JH. Circumcision status and risk of HIV and sexually transmitted infections among men who have sex with men. Journal of the American Medical Association 2008;300(14):1674-84.R01XP08/3; 
Mills E, Cooper C, Anema A, Guyatt G. Male circumcision for the prevention of heterosexually acquired HIV infection: a meta-analysis of randomized trials involving 11,050 men.[see comment]. [Review] [10 refs]. HIV Medicine 2008;9(6):332-5.R07XP08/1; 
Mills EJ, Nachega JB, Buchan I, Orbinski J, Attaran A, Singh S et al. Adherence to antiretroviral therapy in sub-Saharan Africa and North America: a meta-analysis. [Review] [99 refs]. JAMA 2006;296(6):679-90.R14XP08/2; 
Mills EJ, Nachega JB, Bangsberg DR, Singh S, Rachlis B, Wu P et al. Adherence to HAART: A systematic review of developed and developing nation patient-reported barriers and facilitators. PLoS Medicine 3(11)()(pp 2039-2064), 2006 Date of Publication: Nov 2006 2006;(11):2039-64.R16XP08/2; 
Millson P, Challacombe L, Villeneuve PJ, Strike CJ, Fischer B, Myers T et al. Reduction in injection-related HIV risk after 6 months in a low-threshold methadone treatment program. AIDS Education & Prevention 2007;19(2):124-36.R10XP08/1; 
Moore DM, Hogg RS, Yip B, Wood E, Harris M, Montaner JS. Regimen-dependent variations in adherence to therapy and virological suppression in patients initiating protease inhibitor-based highly active antiretroviral therapy. HIV Medicine 2006;7(5):311-6.R14XP08/2; 
Morrison CS, Turner AN, Jones LB. Highly effective contraception and acquisition of HIV and other sexually transmitted infections. Best Practice and Research: Clinical Obstetrics and Gynaecology 23(2)()(pp 263-284), 2009 Date of Publication: April 2009 2009;(2):263-84.R08XP08/1; 
Morrow KM, Fava JL, Rosen RK, Christensen AL, Vargas S, Barroso C. Willingness to use microbicides varies by race/ethnicity, experience with prevention products, and partner type. Health psychology : official journal of the Division of Health Psychology, American Psychological Association 2007;26(6):777-86.R03XP08/3; R04XP08/3; R08XP08/3
Mugavero MJ, Lin HY, Allison JJ, Giordano TP, Willig JH, Raper JL et al. Racial disparities in HIV virologic failure: do missed visits matter? Journal of Acquired Immune Deficiency Syndromes: JAIDS 2009;50(1):100-8.R14XP08/2; 
Mulligan MJ, Russell ND, Celum C, Kahn J, Noonan E, Montefiori DC et al. Excellent safety and tolerability of the human immunodeficiency virus type 1 pGA2/JS2 plasmid DNA priming vector vaccine in HIV type 1 uninfected adults. AIDS Research & Human Retroviruses 2006;22(7):678-83.R16XP08/1; 
Munakata J, Benner JS, Becker S, Dezii CM, Hazard EH, Tierce JC. Clinical and economic outcomes of nonadherence to highly active antiretroviral therapy in patients with human immunodeficiency virus. Medical Care 2006;44(10):893-9.R14XP08/2; 
Murphy DA, Hoffman D, Seage GR, III, Belzer M, Xu J, Durako SJ et al. Improving comprehension for HIV vaccine trial information among adolescents at risk of HIV. AIDS Care 2007;19(1):42-51.R05XP08/1; 
Naeger LK, Struble KA. Effect of baseline protease genotype and phenotype on HIV response to atazanavir/ritonavir in treatment-experienced patients.[see comment]. AIDS 2006;20(6):847-53.R14XP08/2; 
National Coordinating Centre for Health Technology Assessment. Non-occupational post-exposure prophylaxis for HIV: a systematic review (DARE structured abstract). Health Technology Assessment Vol 13 No 14 2009.R16XP08;  QA - 20.01.10
Neu N, Heffernan-Vacca S, Millery M, Stimell M, Brown J. Postexposure prophylaxis for HIV in children and adolescents after sexual assault: a prospective observational study in an urban medical center. Sexually Transmitted Diseases 2007;34(2):65-8.R05XP08/1; R15XP08/1;
Oduyebo OO, Anorlu R, I, Ogunsola FT. The effects of antimicrobial therapy on bacterial vaginosis in non-pregnant women. Cochrane Database of Systematic Reviews: Reviews. Cochrane Database of Systematic Reviews 2009 Issue 3 Chichester (UK): John Wiley & Sons, Ltd 2009.
Okwundu CI, Okoromah CA. Antiretroviral pre-exposure prophylaxis (PrEP) for preventing HIV in high-risk individuals. [Review] [29 refs]. Cochrane Database of Systematic Reviews 2009;(1):CD007189.R16XP08/1; 
Paasche-Orlow MK, Cheng DM, Palepu A, Meli S, Faber V, Samet JH. Health literacy, antiretroviral adherence, and HIV-RNA suppression: a longitudinal perspective. Journal of General Internal Medicine 2006;21(8):835-40.R14XP08/2; 
Pai NP, Tulsky JP, Cohan D, Colford JM, Jr., Reingold AL. Rapid point-of-care HIV testing in pregnant women: a systematic review and meta-analysis. [Review] [35 refs]. Tropical Medicine & International Health 2007;12(2):162-73.R02XP08/3; 
Palepu A, Tyndall MW, Joy R, Kerr T, Wood E, Press N et al. Antiretroviral adherence and HIV treatment outcomes among HIV/HCV co-infected injection drug users: the role of methadone maintenance therapy. Drug & Alcohol Dependence 2006;84(2):188-94.R14XP08/2; 
Peterman TA, Tian LH, Warner L, Satterwhite CL, Metcalf CA, Malotte KC et al. Condom use in the year following a sexually transmitted disease clinic visit. International Journal of STD & AIDS 2009;20(1):9-13.R16XP08/3;  QA - QUERY RCT? cHECKED 21.01.10 - NOT RCT
Petersen ML, Wang Y, van der Laan MJ, Guzman D, Riley E, Bangsberg DR. Pillbox organizers are associated with improved adherence to HIV antiretroviral therapy and viral suppression: a marginal structural model analysis.[see comment]. Clinical Infectious Diseases 2007;45(7):908-15.R14XP08/2; 
Peterson JL, Rothenberg R, Kraft JM, Beeker C, Trotter R. Perceived condom norms and HIV risks among social and sexual networks of young African American men who have sex with men. Health Education Research 2009;24(1):119-27.R01XP08/3; R03XP08/3;
Poynten IM, Smith DE, Cooper DA, Kaldor JM, Grulich AE. The public health impact of widespread availability of nonoccupational postexposure prophylaxis against HIV. HIV Medicine 2007;8(6):374-81.R16XP08/1; 
Poynten M, Brown JM, Sovero M, Millwood IY, Kaldor JM. Microbicide safety and effectiveness: An overview of recent clinical trials. Current Opinion in HIV and AIDS 2008;3(5):574-80.R16XP08/3; 
Priddy FH, Brown D, Kublin J, Monahan K, Wright DP, Lalezari J et al. Safety and immunogenicity of a replication-incompetent adenovirus type 5 HIV-1 clade B gag/pol/nef vaccine in healthy adults. Clinical Infectious Diseases 2008;46(11):1769-81.R16XP08/1; 
Quinn TC. Circumcision and HIV transmission. [Review] [46 refs]. Current Opinion in Infectious Diseases 2007;20(1):33-8.R07XP08/1;  QA - AGREE
Raffa JD, Tossonian HK, Grebely J, Petkau AJ, DeVlaming S, Conway B. Intermediate highly active antiretroviral therapy adherence thresholds and empirical models for the development of drug resistance mutations. Journal of Acquired Immune Deficiency Syndromes: JAIDS 2008;47(3):397-9.R14XP08/2; 
Ramirez-Garcia P, Cote J. Development of a nursing intervention to facilitate optimal antiretroviral-treatment taking among people living with HIV. BMC Health Services Research 2009;9:113.R14XP08/2 - ADHERENCE; 
Rathbun RC, Farmer KC, Lockhart SM, Stephens JR. Validity of a stage of change instrument in assessing medication adherence in indigent patients with HIV infection. Annals of Pharmacotherapy 2007;41(2):208-14.R14XP08/2; 
Rhodes SD, Yee LJ. Using hepatitis A and B vaccination as a paradigm for effective HIV vaccine delivery. [Review] [52 refs]. Sexual Health 2007;4(2):121-7.R01XP08/1;  QA - AGREED
Robison LS, Westfall AO, Mugavero MJ, Kempf MC, Cole SR, Allison JJ et al. Short-term discontinuation of HAART regimens more common in vulnerable patient populations. AIDS Research & Human Retroviruses 2008;24(11):1347-55.R14XP08/2; 
Ross L, Lim ML, Liao Q, Wine B, Rodriguez AE, Weinberg W et al. Prevalence of antiretroviral drug resistance and resistance-associated mutations in antiretroviral therapy-naive HIV-infected individuals from 40 United States cities. HIV Clinical Trials 2007;8(1):1-8.R14XP08/2; 
Rudy BJ, Murphy DA, Harris DR, Muenz L, Ellen J. Patient-related risks for nonadherence to antiretroviral therapy among HIV-infected youth in the United States: A study of prevalence and interactions. AIDS Patient Care and STDs 2009;23(3):185-94.R14XP08/2; 
Safren SA, O'Cleirigh C, Tan JY, Raminani SR, Reilly LC, Otto MW et al. A randomized controlled trial of cognitive behavioral therapy for adherence and depression (CBT-AD) in HIV-infected individuals. Health Psychology 2009;28(1):1-10.R14XP08/2; 
Sayles JN, Wong MD, Cunningham WE. The inability to take medications openly at home: does it help explain gender disparities in HAART use? Journal of Women's Health 2006;15(2):173-81.R14XP08/2; 
Schackman BR, Ribaudo HJ, Krambrink A, Hughes V, Kuritzkes DR, Gulick RM. Racial differences in virologic failure associated with adherence and quality of life on efavirenz-containing regimens for initial HIV therapy: results of ACTG A5095. Journal of Acquired Immune Deficiency Syndromes: JAIDS 2007;46(5):547-54.R14XP08/2; 
Schwartz JL, Mauck C, Lai JJ, Creinin MD, Brache V, Ballagh SA et al. Fourteen-day safety and acceptability study of 6% cellulose sulfate gel: a randomized double-blind Phase I safety study. Contraception 2006;74(2):133-40.R09XP08/3; 
Schwartz JL, Ballagh SA, Kwok C, Mauck CK, Weiner DH, Rencher WF et al. Fourteen-day safety and acceptability study of the universal placebo gel. Contraception 2007;75(2):136-41.R16XP08/1; 
Sharma A, Feldman JG, Golub ET, Schmidt J, Silver S, Robison E et al. Live birth patterns among human immunodeficiency virus-infected women before and after the availability of highly active antiretroviral therapy. American Journal of Obstetrics & Gynecology 2007;196(6):541-6.R02XP08/3;  QA - AGREE
Shepherd BE, Gilbert PB, Jemiai Y, Rotnitzky A. Sensitivity analyses comparing outcomes only existing in a subset selected post-randomization, conditional on covariates, with application to HIV vaccine trials. Biometrics 2006;62(2):332-42.R16XP08/1; 
Shuter J, Bernstein SL. Cigarette smoking is an independent predictor of nonadherence in HIV-infected individuals receiving highly active antiretroviral therapy. Nicotine & Tobacco Research 2008;10(4):731-6.R14XP08/2; 
Simoni JM, Frick PA, Huang B. A longitudinal evaluation of a social support model of medication adherence among HIV-positive men and women on antiretroviral therapy. Health Psychology 2006;25(1):74-81.R14XP08/2;  QA - AGREE
Simoni JM, Pantalone DW, Plummer MD, Huang B. A randomized controlled trial of a peer support intervention targeting antiretroviral medication adherence and depressive symptomatology in HIV-positive men and women. Health Psychology 2007;26(4):488-95.R14XP08/2;  QA - AGREE (BM)
Simoni JM, Pearson CR, Pantalone DW, Marks G, Crepaz N. Efficacy of interventions in improving highly active antiretroviral therapy adherence and HIV-1 RNA viral load. A meta-analytic review of randomized controlled trials. Journal of Acquired Immune Deficiency Syndromes: JAIDS 2006;43 Suppl 1:S23-S35.R14XP08/2; 
Sobieszcyk ME, Xu G, Goodman K, Lucy D, Koblin BA. Engaging members of African American and Latino communities in preventive HIV vaccine trials. Journal of Acquired Immune Deficiency Syndromes: JAIDS 2009;51(2):194-201.R16XP08/1; 
Song J, Lee MB, Rotheram-Borus MJ, Swendeman D. Predictors of intervention adherence among young people living with HIV. American Journal of Health Behavior 2006;30(2):136-46.R14XP08/2;  COPIED FROM RCTs
Sorensen JL, Haug NA, Delucchi KL, Gruber V, Kletter E, Batki SL et al. Voucher reinforcement improves medication adherence in HIV-positive methadone patients: a randomized trial. Drug & Alcohol Dependence 2007;88(1):54-63.R14XP08/2; 
Stevens PE, Galvao L. "He won't use condoms": HIV-infected women's struggles in primary relationships with serodiscordant partners. American Journal of Public Health 2007;97(6):1015-22.R08XP08/2; 
Stoltz JA, Wood E, Small W, Li K, Tyndall M, Montaner J et al. Changes in injecting practices associated with the use of a medically supervised safer injection facility. Journal of Public Health 2007;29(1):35-9.R10XP08/1; 
Stoner SA, Norris J, George WH, Morrison DM, Zawacki T, Davis KC et al. Women's condom use assertiveness and sexual risk-taking: effects of alcohol intoxication and adult victimization. Addictive Behaviors 2008;33(9):1167-76.R08XP08/3; R10XP08/3; R15XP08/3
Suksomboon N, Poolsup N, Ket-Aim S. Systematic review of the efficacy of antiretroviral therapies for reducing the risk of mother-to-child transmission of HIV infection. [Review] [41 refs]. Journal of Clinical Pharmacy & Therapeutics 2007;32(3):293-311.R02XP08/3; 
Sullivan LE, Moore BA, Chawarski MC, Pantalon MV, Barry D, O'Connor PG et al. Buprenorphine/naloxone treatment in primary care is associated with decreased human immunodeficiency virus risk behaviors. Journal of Substance Abuse Treatment 2008;35(1):87-92.R10XP08/3;  QA - QUERY - NOT RCT (was included, but QA changed decision)
Tavel JA, Martin JE, Kelly GG, Enama ME, Shen JM, Gomez PL et al. Safety and immunogenicity of a Gag-Pol candidate HIV-1 DNA vaccine administered by a needle-free device in HIV-1-seronegative subjects. Journal of Acquired Immune Deficiency Syndromes: JAIDS 2007;44(5):601-5.R16XP08/1; 
Thiede H, Hagan H, Campbell JV, Strathdee SA, Bailey SL, Hudson SM et al. Prevalence and correlates of indirect sharing practices among young adult injection drug users in five U.S. cities. Drug and Alcohol Dependence 2007;91 Suppl 1:S39-S47.R10XP08/3; 
Thongcharoen P, Suriyanon V, Paris RM, Khamboonruang C, de Souza MS, Ratto-Kim S et al. A phase 1/2 comparative vaccine trial of the safety and immunogenicity of a CRF01_AE (subtype E) candidate vaccine: ALVAC-HIV (vCP1521) prime with oligomeric gp160 (92TH023/LAI-DID) or bivalent gp120 (CM235/SF2) boost. Journal of Acquired Immune Deficiency Syndromes: JAIDS 2007;46(1):48-55.R16XP08/1; 
Tyndall MW, McNally M, Lai C, Zhang R, Wood E, Kerr T et al. Directly observed therapy programmes for anti-retroviral treatment amongst injection drug users in Vancouver: access, adherence and outcomes. International Journal of Drug Policy 2007;18(4):281-7.R14XP08/2; 
Vardi Y, Sadeghi-Nejad H, Pollack S, isuodionoe-Shadrach OI, Sharlip ID. Male circumcision and HIV prevention. [Review] [19 refs]. Journal of Sexual Medicine 2007;4(4 Pt 1):838-43.R07XP08/1; 
Villafranca SW, McKellar JD, Trafton JA, Humphreys K. Predictors of retention in methadone programs: a signal detection analysis. Drug & Alcohol Dependence 2006;83(3):218-24.R10XP08/3;  QA - AGREE
Volmink J, Siegfried N, van der ML, Brocklehurst P. Antiretrovirals for reducing the risk of mother-to-child transmission of HIV infection. Cochrane Database of Systematic Reviews: Reviews. Cochrane Database of Systematic Reviews 2007 Issue 1 Chichester (UK): John Wiley & Sons, Ltd 2007.
Vranceanu AM, Safren SA, Lu M, Coady WM, Skolnik PR, Rogers WH et al. The relationship of post-traumatic stress disorder and depression to antiretroviral medication adherence in persons with HIV. AIDS Patient Care & Stds 2008;22(4):313-21.R14XP08/2; 
Wagner GJ, Kanouse DE, Golinelli D, Miller LG, Daar ES, Witt MD et al. Cognitive-behavioral intervention to enhance adherence to antiretroviral therapy: a randomized controlled trial (CCTG 578). AIDS 2006;20(9):1295-302.R14XP08/2;  QA - AGREE (BM)
Warren E, Viney R, Shearer J, Shanahan M, Wodak A, Dolan K. Value for money in drug treatment: economic evaluation of prison methadone. Drug & Alcohol Dependence 2006;84(2):160-6.R13XP08/1; 
Weiss HA. Male circumcision as a preventive measure against HIV and other sexually transmitted diseases. Current Opinion in Infectious Diseases 20(1)()(pp 66-72), 2007 Date of Publication: Feb 2007 2007;(1):66-72.R07XP08/1; 
Weiss HA, Wasserheit JN, Barnabas RV, Hayes RJ, bu-Raddad LJ. Persisting with prevention: The importance of adherence for HIV prevention. Emerging Themes in Epidemiology 5, 2008 Article Number: 8 Date of Publication: 2008 2008.R14XP08/2;  QUERY COPIED FROM RCT
Weiss HA, Wasserheit JN, Barnabas RV, Hayes RJ, bu-Raddad LJ. Persisting with prevention: The importance of adherence for HIV prevention. Emerging Themes in Epidemiology 2008;5, 2008. Article Number.R14XP08/2; 
Westling E, Garcia K, Mann T. Discovery of meaning and adherence to medications in HIV-infected women. Journal of Health Psychology 2007;12(4):627-35.R14XP08/2;  QA - QUERY include R08? - no HIV trumps other risk groups
Williams AB, Fennie KP, Bova CA, Burgess JD, Danvers KA, Dieckhaus KD. Home visits to improve adherence to highly active antiretroviral therapy: a randomized controlled trial. Journal of Acquired Immune Deficiency Syndromes: JAIDS 2006;42(3):314-21.R14XP08/2; 
Wise J, Operario D. Use of electronic reminder devices to improve adherence to antiretroviral therapy: a systematic review. [Review] [51 refs]. AIDS Patient Care & Stds 2008;22(6):495-504.R14XP08/2; 
Witte SS, El-Bassel N, Gilbert L, Wu E, Chang M, Hill J. Promoting female condom use to heterosexual couples: findings from a randomized clinical trial. Perspectives on sexual and reproductive health 2006;38(3):148-54.R09XP08/3; 
Wiysonge CS, Shey MS, Shang J, Kongnyuy EJ, Brocklehurst P. Vaginal microbicides for preventing mother-to-child transmission of HIV infection--no evidence of an effect or evidence of no effect?. [Review] [20 refs]. South African Medical Journal 2007;Suid-Afrikaanse Tydskrif Vir Geneeskunde. 97(7):530-3.R02XP08/3; 
Wood E, Stoltz J-A, Montaner JSG, Kerr T. Evaluating methamphetamine use and risks of injection initiation among street youth: The ARYS study. Harm Reduction Journal 2006;3, 2006. Article Number.R05XP08/1; R10XP08/1;
Wood E, Lloyd-Smith E, Li K, Strathdee SA, Small W, Tyndall MW et al. Frequent needle exchange use and HIV incidence in Vancouver, Canada. American Journal of Medicine 2007;120(2):172-9.R10XP08/3; 
Wright D, Rodriguez A, Godofsky E, Walmsley S, Labriola-Tompkins E, Donatacci L et al. Efficacy and safety of 48 weeks of enfuvirtide 180 mg once-daily dosing versus 90 mg twice-daily dosing in HIV-infected patients. HIV Clinical Trials 2008;9(2):73-82.R14XP08/2; 
Young TN, Arens FJ, Kennedy GE, Laurie JW, Rutherford G. Antiretroviral post-exposure prophylaxis (PEP) for occupational HIV exposure. [Review] [33 refs]. Cochrane Database of Systematic Reviews 2007;(1):CD002835.R16XP08/1; 
Zule WA, Bobashev G. High dead-space syringes and the risk of HIV and HCV infection among injecting drug users. Drug and Alcohol Dependence 2009;100(3):204-13.R10XP08/3;
2 codes
Broaddus MR, Bryan A. Consistent condom use among juvenile detainees: the role of individual differences, social bonding, and health beliefs. AIDS & Behavior 2008;12(1):59-67.R05XP08/1; R13XP08/1; QA - AGREE (BM)
Coffin PO, Latka MH, Latkin C, Wu Y, Purcell DW, Metsch L et al. Safe syringe disposal is related to safe syringe access among HIV-positive injection drug users. AIDS & Behavior 2007;11(5):652-62.R14XP08/2; R10XP08/2;
Crosby R, DiClemente RJ, Yarber WL, Snow G, Troutman A. An event-specific analysis of condom breakage among African American men at risk of HIV acquisition. Sexually Transmitted Diseases 2008;35(2):174-7.R03XP08/1; R07XP08/1;
Girardet RG, Lemme S, Biason TA, Bolton K, Lahoti S. HIV post-exposure prophylaxis in children and adolescents presenting for reported sexual assault. Child Abuse & Neglect 2009;33(3):173-8.R15XP08/1; R05XP08/1; QA - AGREE
Golub ET, Strathdee SA, Bailey SL, Hagan H, Latka MH, Hudson SM et al. Distributive syringe sharing among young adult injection drug users in five U.S. cities. Drug & Alcohol Dependence 2007;91 Suppl 1:S30-S38.R10XP08/1; R05XP08/1;
Hagan H, Campbell JV, Thiede H, Strathdee SA, Ouellet L, Latka M et al. Injecting alone among young adult IDUs in five US cities: evidence of low rates of injection risk behavior. Drug & Alcohol Dependence 2007;91 Suppl 1:S48-S55.R10XP08/1; R05XP08/1;
Kapadia F, Latka MH, Hudson SM, Golub ET, Campbell JV, Bailey S et al. Correlates of consistent condom use with main partners by partnership patterns among young adult male injection drug users from five US cities. Drug and Alcohol Dependence 2007;91 Suppl 1:S56-S63.R10XP08/3; R05XP08/3; R07XP08/3; COPIED FROM RCT TO COHORTS
Larney S, Dolan K. A literature review of international implementation of opioid substitution treatment in prisons: equivalence of care?. [Review] [52 refs]. European Addiction Research 2009;15(2):107-12.R10XP08/3; R13XP08/3;
Macalino GE, Hogan JW, Mitty JA, Bazerman LB, DeLong AK, Loewenthal H et al. A randomized clinical trial of community-based directly observed therapy as an adherence intervention for HAART among substance users. AIDS 2007;21(11):1473-7.R14XP08/2; R10XP08/2;
Mantell JE, Morar NS, Myer L, Ramjee G. "We have our protector": misperceptions of protection against HIV among participants in a microbicide efficacy trial. American Journal of Public Health 2006;96(6):1073-7.R12XP08/1; R08XP08/1;
Maru DS, Bruce RD, Walton M, Springer SA, Altice FL. Persistence of virological benefits following directly administered antiretroviral therapy among drug users: results from a randomized controlled trial. Journal of Acquired Immune Deficiency Syndromes: JAIDS 2009;50(2):176-81.R14XP08/2; R14XP08/2;
Morrow KM, Fava JL, Rosen RK, Christensen AL, Vargas S, Barroso C. Willingness to use microbicides varies by race/ethnicity, experience with prevention products, and partner type. Health psychology : official journal of the Division of Health Psychology, American Psychological Association 2007;26(6):777-86.R03XP08/3; R04XP08/3; R08XP08/3
Neu N, Heffernan-Vacca S, Millery M, Stimell M, Brown J. Postexposure prophylaxis for HIV in children and adolescents after sexual assault: a prospective observational study in an urban medical center. Sexually Transmitted Diseases 2007;34(2):65-8.R05XP08/1; R15XP08/1;
Peterson JL, Rothenberg R, Kraft JM, Beeker C, Trotter R. Perceived condom norms and HIV risks among social and sexual networks of young African American men who have sex with men. Health Education Research 2009;24(1):119-27.R01XP08/3; R03XP08/3;
Stoner SA, Norris J, George WH, Morrison DM, Zawacki T, Davis KC et al. Women's condom use assertiveness and sexual risk-taking: effects of alcohol intoxication and adult victimization. Addictive Behaviors 2008;33(9):1167-76.R08XP08/3; R10XP08/3; R15XP08/3
Wood E, Stoltz J-A, Montaner JSG, Kerr T. Evaluating methamphetamine use and risks of injection initiation among street youth: The ARYS study. Harm Reduction Journal 2006;3, 2006. Article Number.R05XP08/1; R10XP08/1;
 3 codes
Kapadia F, Latka MH, Hudson SM, Golub ET, Campbell JV, Bailey S et al. Correlates of consistent condom use with main partners by partnership patterns among young adult male injection drug users from five US cities. Drug and Alcohol Dependence 2007;91 Suppl 1:S56-S63.R10XP08/3; R05XP08/3; R07XP08/3; COPIED FROM RCT TO COHORTS
Morrow KM, Fava JL, Rosen RK, Christensen AL, Vargas S, Barroso C. Willingness to use microbicides varies by race/ethnicity, experience with prevention products, and partner type. Health psychology : official journal of the Division of Health Psychology, American Psychological Association 2007;26(6):777-86.R03XP08/3; R04XP08/3; R08XP08/3
Stoner SA, Norris J, George WH, Morrison DM, Zawacki T, Davis KC et al. Women's condom use assertiveness and sexual risk-taking: effects of alcohol intoxication and adult victimization. Addictive Behaviors 2008;33(9):1167-76.R08XP08/3; R10XP08/3; R15XP08/3

Descriptive epidemiology (P09)
1 code
Adimora AA, Schoenbach VJ, Martinson FEA, Coyne-Beasley T, Doherty I, Stancil TR et al. Heterosexually transmitted HIV infection among African Americans in North Carolina. Journal of Acquired Immune Deficiency Syndromes 2006;41(5):616-23.R03XP09/3; R09XP09/3;
Aggarwal I, Smith M, Tatt ID, Murad S, Osner N, Geretti AM et al. Evidence for onward transmission of HIV-1 non-B subtype strains in the United Kingdom. Journal of Acquired Immune Deficiency Syndromes: JAIDS 2006;41(2):201-9.R03XP09/1; 
Aidala AA, Lee G, Howard JM, Caban M, Abramson D, Messeri P. HIV-positive men sexually active with women: sexual behaviors and sexual risks. Journal of Urban Health 2006;83(4):637-55.R14XP09/2; 
Baillargeon JG, Paar DP, Wu H, Giordano TP, Murray O, Raimer BG et al. Psychiatric disorders, HIV infection and HIV/hepatitis co-infection in the correctional setting. AIDS Care 2008;20(1):124-9.R13XP09/3; R15XP09/3;
Bautista CT, Sateren WB, Sanchez JL, Rathore Z, Singer DE, Birx DL et al. HIV incidence trends among white and African-American active duty United States Army personnel (1986-2003). Journal of Acquired Immune Deficiency Syndromes 2006;43(3):351-5.R13XP09/3; 
Beckwith CG, DeLong AK, Desjardins SF, Gillani F, Bazerman L, Mitty JA et al. HIV infection in refugees: a case-control analysis of refugees in Rhode Island. International Journal of Infectious Diseases 2009;13(2):186-92.R03XP09/3; 
Bennett WR, Joesch JM, Mazur M, Roy-Byrne P. Characteristics of HIV-positive patients treated in a psychiatric emergency department. Psychiatric Services 2009;60(3):398-401.R15XP09/3; 
Brennan DJ, Hellerstedt WL, Ross MW, Welles SL. History of childhood sexual abuse and HIV risk behaviors in homosexual and bisexual men. American Journal of Public Health 2007;97(6):1107-12.R15XP09/3; 
Brewer-Smyth K, Bucurescu G, Shults J, Metzger D, Sacktor N, van GW et al. Neurological function and HIV risk behaviors of female prison inmates. Journal of Neuroscience Nursing 2007;39(6):361-72.R13XP09/3; R08XP09/3;
Burt RD, Hagan H, Garfein RS, Sabin K, Weinbaum C, Thiede H. Trends in hepatitis B virus, hepatitis C virus, and human immunodeficiency virus prevalence, risk behaviors, and preventive measures among Seattle injection drug users aged 18-30 years, 1994-2004. Journal of Urban Health 2007;84(3):436-54.R10XP09/3; 
Calzavara L, Ramuscak N, Burchell AN, Swantee C, Myers T, Ford P et al. Prevalence of HIV and hepatitis C virus infections among inmates of Ontario remand facilities.[see comment][erratum appears in CMAJ. 2007 Aug 28;177(5):489]. CMAJ Canadian Medical Association Journal 2007;177(3):257-61.R13XP09/2; 
Cedar PP, Pearce ME, Christian WM, Patterson K, Norris K, Moniruzzaman A et al. The Cedar Project: historical trauma, sexual abuse and HIV risk among young Aboriginal people who use injection and non-injection drugs in two Canadian cities. Social Science & Medicine 2008;66(11):2185-94.R10XP09/3; R04XP09/3;
Creighton S, Tariq S, Perry G. Sexually transmitted infections among UK street-based sex workers. Sexually Transmitted Infections 2008;84(1):32-3.R12XP09/3;  QA - AGREE
Davis M, Hart G, Bolding G, Sherr L, Elford J. Sex and the internet: gay men, risk reduction and serostatus. Culture 2006;8(2):161-74.R01XP09/3; 
Degenhardt L, Hall W, Warner-Smith M. Using cohort studies to estimate mortality among injecting drug users that is not attributable to AIDS. [Review] [110 refs]. Sexually Transmitted Infections 2006;82 Suppl 3:iii56-iii63.R10XP09/3; 
Dodds JP, Johnson AM, Parry JV, Mercey DE. A tale of three cities: persisting high HIV prevalence, risk behaviour and undiagnosed infection in community samples of men who have sex with men.[erratum appears in Sex Transm Infect. 2007 Oct;83(6):500]. Sexually Transmitted Infections 2007;83(5):392-6.R01XP09/3; R01XP06/3;
Drummond PD, Mizan A, Wright B. HIV/AIDS knowledge and attitudes among West African immigrant women in Western Australia. Sexual Health 2008;5(3):251-9.R03XP09/3; R08XP09/3;
Edwards JM, Iritani BJ, Hallfors DD. Prevalence and correlates of exchanging sex for drugs or money among adolescents in the United States. Sexually Transmitted Infections 2006;82(5):354-8.R05XP09/3; R12XP09/3; QA - AGREE
El-Bassel N, Gilbert L, Wu E, Chang M, Gomes C, Vinocur D et al. Intimate partner violence prevalence and HIV risks among women receiving care in emergency departments: implications for IPV and HIV screening. Emergency Medicine Journal 2007;24(4):255-9.R15XP09/3; R08XP09/3;
Elam G, Macdonald N, Hickson FCI, Imrie J, Power R, McGarrigle CA et al. Risky sexual behaviour in context: qualitative results from an investigation into risk factors for seroconversion among gay men who test for HIV. Sexually Transmitted Infections 2008;84(6):473-7.R01XP09/3; 
Eyzaguirre L, Brouwer KC, Nadai Y, Patterson TL, Ramos R, Firestone CM et al. First molecular surveillance report of HIV type 1 in injecting drug users and female sex workers along the U.S.-Mexico border. AIDS Research & Human Retroviruses 2007;23(2):331-4.R15XP09/3; R10XP09/3;
Fisher HH, Eke AN, Cance JD, Hawkins SR, Lam WK. Correlates of HIV-related risk behaviors in African American adolescents from substance-using families: patterns of adolescent-level factors associated with sexual experience and substance use. Journal of Adolescent Health 2008;42(2):161-9.R03XP09/3; R05XP09/3; R15XP09/3
Forna FM, Fitzpatrick L, Adimora AA, Lellan-Lemal E, Leone P, Brooks JT et al. A case-control study of factors associated with HIV infection among black women. Journal of the National Medical Association 2006;98(11):1798-804.R03XP09/3; 
Gallagher KM, Sullivan PS, Lansky A, Onorato IM. Behavioral surveillance among people at risk for HIV infection in the U.S.: The national HIV behavioral surveillance system. Public Health Reports 2007;122(SUPPL. 1):32-8.R01XP09/1; R09XP09/1; R10XP09/1; COPIED FROM SR
Garofalo R, Mustanski BS, McKirnan DJ, Herrick A, Donenberg GR. Methamphetamine and young men who have sex with men: understanding patterns and correlates of use and the association with HIV-related sexual risk. Archives of Pediatrics & Adolescent Medicine 2007;161(6):591-6.R10XP09/3; R01XP09/3; R05XP09/3
George C, Alary M, Hogg RS, Otis J, Remis RS, Masse B et al. HIV and ethnicity in Canada: is the HIV risk-taking behaviour of young foreign-born MSM similar to Canadian born MSM?  AIDS Care 2007;19(1):9-16.R01XP09/3; 
George C, Alary M, Otis J, Demers E, Masse B, Lavoie R et al. Nonnegligible increasing temporal trends in unprotected anal intercourse among men who have sexual relations with other men in Montreal. Journal of Acquired Immune Deficiency Syndromes 2006;41(3):365-70.R01XP09/1; 
Grulich AE, Kaldor JM. Trends in HIV incidence in homosexual men in developed countries. [Review] [58 refs]. Sexual Health 2008;5(2):113-8.R14XP09/3; 
Guy RJ, McDonald AM, Bartlett MJ, Murray JC, Giele CM, Davey TM et al. Characteristics of HIV diagnoses in Australia, 1993-2006. Sexual Health 2008;5(2):91-6.R14XP09/3; 
Hallfors DD, Iritani BJ, Miller WC, Bauer DJ. Sexual and drug behavior patterns and HIV and STD racial disparities: the need for new directions. American Journal of Public Health 2007;97(1):125-32.R10XP09/3; R04XP09/3;
Hamers FF, Phillips AN. Diagnosed and undiagnosed HIV-infected populations in Europe. HIV Medicine 2008;9(SUPPL. 2):6-12.R16XP09/3; 
Hammett TM, Drachman-Jones A. HIV/AIDS, sexually transmitted diseases, and incarceration among women: National and southern perspectives. Sexually Transmitted Diseases 2006;33(7 SUPPL.):S17-S22.R03XP09/3; R08XP09/3; R13XP09/3
Hebert MR, Rose JS, Rosengard C, Clarke JG, Stein MD. Levels of trauma among women inmates with HIV risk and alcohol use disorders: behavioral and emotional impacts. Journal of Trauma & Dissociation 2007;8(2):27-46.R08XP09/3; R13XP09/3;
Herbst JH, Jacobs ED, Finlayson TJ, McKleroy VS, Neumann MS, Crepaz N et al. Estimating HIV prevalence and risk behaviors of transgender persons in the United States: a systematic review. [Review] [90 refs]. AIDS & Behavior 2008;12(1):1-17.R11XP09/3; 
Hightow LB, Leone PA, Macdonald PD, McCoy SI, Sampson LA, Kaplan AH. Men who have sex with men and women: a unique risk group for HIV transmission on North Carolina College campuses.[see comment]. Sexually Transmitted Diseases 2006;33(10):585-93.R01XP09/3; R09XP09/3;
Houck CD, Hadley W, Lescano CM, Pugatch D, Brown LK, Project SHIELD Study Group. Suicide attempt and sexual risk behavior: relationship among adolescents. Archives of Suicide Research 2008;12(1):39-49.R05XP09/1; 
Jin F, Prestage GP, McDonald A, Ramacciotti T, Imrie JC, Kippax SC et al. Trend in HIV incidence in a cohort of homosexual men in Sydney: data from the Health in Men Study. Sexual Health 2008;5(2):109-12.R01XP09/1; 
Kaufman CE, Shelby L, Mosure DJ, Marrazzo J, Wong D, De RL et al. Within the hidden epidemic: Sexually transmitted diseases and HIV/AIDS among American Indians and Alaska natives.  Sexually Transmitted Diseases 2007;34(10):767-77.R04XP09/3; 
Kelley CF, Hernandez-Ramos I, Franco-Paredes C, del RC. Clinical, epidemiologic characteristics of foreign-born Latinos with HIV/AIDS at an urban HIV clinic.[see comment]. AIDS Reader 1978;17(2):73-4.R14XP09/2;  QA - AGREE
Kerr T, Stoltz JA, Strathdee S, Li K, Hogg RS, Montaner JS et al. The impact of sex partners' HIV status on HIV seroconversion in a prospective cohort of injection drug users. Journal of Acquired Immune Deficiency Syndromes: JAIDS 2006;41(1):119-23.R10XP09/3; 
Kiarie JN, Farquhar C, Richardson BA, Kabura MN, John FN. Domestic violence and prevention of mother-to-child transmission of HIV-1. AIDS 2007;20(13):1763-9.R02XP09/3; 
Kremer H, Ironson G. To tell or not to tell: why people with HIV share or don't share with their physicians whether they are taking their medications as prescribed. AIDS Care 2006;18(5):520-8.R14XP09/2; 
Lampinen TM, Mattheis K, Chan K, Hogg RS. Nitrite inhalant use among young gay and bisexual men in Vancouver during a period of increasing HIV incidence. BMC Public Health 2007;7:35.R10XP09/1; R01XP09/1; QA - MSM - code added 8.03.10
Lavoie E, Alary M, Remis RS, Otis J, Vincelette J, Turmel B et al. Determinants of HIV seroconversion among men who have sex with men living in a low HIV incidence population in the era of highly active antiretroviral therapies. Sexually Transmitted Diseases 2008;35(1):25-9.R01XP09/1; 
Lee LM, McKenna MT. Monitoring the incidence of HIV infection in the United States. Public Health Reports 2007;122(SUPPL. 1):72-9.R16XP09/3; R16XP13/3; QA - QUERY - is this prevention or measuring prevention? does this matter?! COPIED FROM SR - Code added METHODOLOGY 08.03.10
MacKellar DA, Valleroy LA, Behel S, Secura GM, Bingham T, Celentano DD et al. Unintentional HIV exposures from young men who have sex with men who disclose being HIV-negative. AIDS 2006;20(12):1637-44.R01XP09/3; 
March JC, Oviedo-Joekes E, Romero M. Factors associated with reported hepatitis C and HIV among injecting drug users in ten European cities. Enfermedades Infecciosas y Microbiologia Clinica 2007;25(2):91-7.R10XP09/3; R13XP09/3;
Marks G, Millett GA, Bingham T, Bond L, Lauby J, Liau A et al. Understanding differences in HIV sexual transmission among Latino and black men who have sex with men: The Brothers y Hermanos Study. AIDS & Behavior 2009;13(4):682-90.R01XP09/3; R03XP09/3; R04XP09/3
Marshall BD, Wood E, Li K, Kerr T. Elevated syringe borrowing among men who have sex with men: a prospective study. Journal of Acquired Immune Deficiency Syndromes: JAIDS 2007;46(2):248-52.R10XP09/3; R01XP09/3;
Mathers BM, Degenhardt L, Phillips B, Wiessing L, Hickman M, Strathdee SA et al. Global epidemiology of injecting drug use and HIV among people who inject drugs: a systematic review.[see comment]. [Review] [22 refs]. Lancet 2008;372(9651):1733-45.R10XP09/3; 
McAllister SM, Dickson NP, Sharples K, Reid MR, Morgan JM, MacDonald EJ et al. Unlinked anonymous HIV prevalence among New Zealand sexual health clinic attenders: 2005-2006. International Journal of STD & AIDS 2008;19(11):752-7.R16XP09/3; 
McKenna MT, Hu X. Recent trends in the incidence and morbidity that are associated with perinatal human immunodeficiency virus infection in the United States. American Journal of Obstetrics and Gynecology 2007;197(3 SUPPL.):S10-S16.R02XP09/3; 
McQuillan GM, Kruszon-Moran D, Kottiri BJ, Kamimoto LA, Lam L, Cowart MF et al. Prevalence of HIV in the US household population: The national health and nutrition examination surveys, 1988 to 2002. Journal of Acquired Immune Deficiency Syndromes 2006;41(5):651-6.R16XP09/3;  COPIED FROM SR
Mehrabadi A, Craib KJP, Patterson K, Adam W, Moniruzzaman A, Ward-Burkitt B et al. The Cedar Project: A comparison of HIV-related vulnerabilities amongst young Aboriginal women surviving drug use and sex work in two Canadian cities. International Journal of Drug Policy 2008;19(2):159-68.R04XP09/3; R08XP09/3; R12XP09/3
Mehta SH, Galai N, Astemborski J, Celentano DD, Strathdee SA, Vlahov D et al. HIV incidence among injection drug users in Baltimore, Maryland (1988-2004). Journal of Acquired Immune Deficiency Syndromes: JAIDS 2006;43(3):368-72.R10XP09/1; 
Melendez RM, Exner TA, Ehrhardt AA, Dodge B, Remien RH, Rotheram-Borus MJ et al. Health and health care among male-to-female transgender persons who are HIV positive. American Journal of Public Health 2006;96(6):1034-7.R14XP09/2; R11XP09/2;
Merchant RC, Nettleton JE, Mayer KH, Becker BM. Blood or body fluid exposures and HIV postexposure prophylaxis utilization among first responders. Prehospital Emergency Care 2009;13(1):6-13.R16XP09/1; 
Merchant RC, Chee KJ, Liu T, Mayer KH. Incidence of visits for health care worker blood or body fluid exposures and HIV postexposure prophylaxis provision at Rhode Island emergency departments. Journal of Acquired Immune Deficiency Syndromes: JAIDS 2008;47(3):358-68.R16XP09/1 - HEALTH WORKERS/PEP; 
Merchant RC, Nettleton JE, Mayer KH, Becker BM. HIV post-exposure prophylaxis among police and corrections officers. Occupational Medicine 2008;58(7):502-5.R16XP09/1; 
Miller CL, Strathdee SA, Li K, Kerr T, Wood E. A longitudinal investigation into excess risk for blood-borne infection among young injection drug users (IUDs). American Journal of Drug & Alcohol Abuse 2007;33(4):527-36.R10XP09/1; 
Miller CL, Kerr T, Frankish JC, Spittal PM, Li K, Schechter MT et al. Binge drug use independently predicts HIV seroconversion among injection drug users: implications for public health strategies.[see comment]. Substance Use & Misuse 2006;41(2):199-210.R10XP09/3; 
Miller M, Korves CT, Fernandez T. The social epidemiology of HIV transmission among African American women who use drugs and their social network members. AIDS Care 2007;19(7):858-65.R03XP09/3; R10XP09/3; R08XP09/3
Millett GA, Flores SA, Peterson JL, Bakeman R. Explaining disparities in HIV infection among black and white men who have sex with men: a meta-analysis of HIV risk behaviors. AIDS 2007;21(15):2083-91.R01XP09/3; 
Millett GA, Peterson JL, Wolitski RJ, Stall R. Greater risk for HIV infection of black men who have sex with men: a critical literature review. [Review] [148 refs]. American Journal of Public Health 2006;96(6):1007-19.R01XP09/3; R03XP09/3;
Morris M, Handcock MS, Miller WC, Ford CA, Schmitz JL, Hobbs MM et al. Prevalence of HIV infection among young adults in the United States: results from the Add Health study. American Journal of Public Health 2006;96(6):1091-7.R05XP09/3; 
Murphy DA, Brecht ML, Herbeck D, Evans E, Huang D, Hser YI. Longitudinal HIV risk behavior among the Drug Abuse Treatment Outcome Studies (DATOS) adult sample. Evaluation Review 2008;32(1):83-112.R10XP09/3; 
Mustanski B, Garofalo R, Herrick A, Donenberg G. Psychosocial health problems increase risk for HIV among urban young men who have sex with men: preliminary evidence of a syndemic in need of attention. Annals of Behavioral Medicine 2007;34(1):37-45.R01XP09/3; R05XP09/3;
Operario D, Soma T, Underhill K. Sex work and HIV status among transgender women: Systematic review and meta-analysis. Journal of Acquired Immune Deficiency Syndromes 2008;48(1):97-103.R11XP09/3; R12XP09/3;
Patterson KB, Leone PA, Fiscus SA, Kuruc J, McCoy SI, Wolf L et al. Frequent detection of acute HIV infection in pregnant women. AIDS 2007;21(17):2303-8.R02XP09/3; 
Pence BW, Thielman NM, Whetten K, Ostermann J, Kumar V, Mugavero MJ. Coping strategies and patterns of alcohol and drug use among HIV-infected patients in the United States Southeast. AIDS Patient Care & Stds 2008;22(11):869-77.R14XP09/2; 
Pottie K, Janakiram P, Topp P, McCarthy A. Prevalence of selected preventable and treatable diseases among government-assisted refugees: Implications for primary care providers. Canadian Family Physician 2007;53(11):1928-34.R03XP09/3; 
Poulin C, Alary M, Lambert G, Godin G, Landry S, Gagnon H et al. Prevalence of HIV and hepatitis C virus infections among inmates of Quebec provincial prisons.[see comment]. CMAJ Canadian Medical Association Journal 2007;177(3):252-6.R13XP09/2; R10XP09/2; QA - Drug users - code added 08.03.10
Prestage G, Mao L, Jin F, Grulich A, Kaldor J, Kippax S. Sex work and risk behaviour among HIV-negative gay men. AIDS Care 2007;19(7):931-4.R01XP09/1; R12XP09/1; QA - AGREE
Prestage G, Jin F, Zablotska I, Grulich A, Imrie J, Kaldor J et al. Trends in agreements between regular partners among gay men in Sydney, Melbourne and Brisbane, Australia. AIDS & Behavior 2008;12(3):513-20.R01XP09/3; 
Prestage G, Jin F, Zablotska I, Imrie J, Kaldor JM, Grulich AE. Trends in HIV prevalence among homosexual and bisexual men in eastern Australian states. Sexual Health 2008;5(2):103-7.R01XP09/3; 
Prestage G, Fogarty AS, Rawstorne P, Grierson J, Zablotska I, Grulich A et al. Use of illicit drugs among gay men living with HIV in Sydney. AIDS 2007;21(SUPPL. 1):S49-S55.R01XP09/3; R10XP09/3;
Raj A, Reed E, Welles SL, Santana MC, Silverman JG. Intimate partner violence perpetration, risky sexual behavior, and STI/HIV diagnosis among heterosexual African American men. American Journal of Mens Health 2008;2(3):291-5.R03XP09/3; R09XP09/3;
Rangel MC, Gavin L, Reed C, Fowler MG, Lee LM. Epidemiology of HIV and AIDS among adolescents and young adults in the United States.[see comment]. Journal of Adolescent Health 2006;39(2):156-63.R05XP09/3; 
Ravi A, Blankenship KM, Altice FL. The association between history of violence and HIV risk: a cross-sectional study of HIV-negative incarcerated women in Connecticut. Womens Health Issues 2007;17(4):210-6.R08XP09/1; R13XP09/1; R15XP09/1
Raymond HF, McFarland W. Racial mixing and HIV risk among men who have sex with men. AIDS & Behavior 2009;13(4):630-7.R01XP09/3; R03XP09/3; R04XP09/3; QA - AGREE
Romero EG, Teplin LA, McClelland GM, Abram KM, Welty LJ, Washburn JJ. A longitudinal study of the prevalence, development, and persistence of HIV/sexually transmitted infection risk behaviors in delinquent youth: implications for health care in the community. Pediatrics 2007;119(5):e1126-e1141.R10XP09/3; R05XP09/3; R13XP09/3
Santibanez SS, Garfein RS, Swartzendruber A, Purcell DW, Paxton LA, Greenberg AE. Update and overview of practical epidemiologic aspects of HIV/AIDS among injection drug users in the United States. Journal of Urban Health 2006;83(1):86-100.R10XP09/3; 
Saylors K, Daliparthy N. Violence against Native women in substance abuse treatment. American Indian & Alaska Native Mental Health Research (Online) 2006;13(1):32-51.R04XP09/3; R15XP09/3;
Schlosser AV, Abdallah AB, Callahan CL, Bradford S, Cottler LB. Does readiness to change predict reduced crack use in human immunodeficiency virus prevention? Journal of Substance Abuse Treatment 2008;35(1):28-35.R10XP09/1; 
Schwarcz S, Scheer S, McFarland W, Katz M, Valleroy L, Chen S et al. Prevalence of HIV infection and predictors of high-transmission sexual risk behaviors among men who have sex with men. American Journal of Public Health 2007;97(6):1067-75.R01XP09/3; 
Semple SJ, Zians J, Grant I, Patterson TL. Sexual risk behavior of HIV-positive methamphetamine-using men who have sex with men: the role of partner serostatus and partner type. Archives of Sexual Behavior 2006;35(4):461-71.R14XP09/2; 
Sethi G, Holden BM, Gaffney J, Greene L, Ghani AC, Ward H. HIV, sexually transmitted infections, and risk behaviours in male sex workers in London over a 10 year period. Sexually Transmitted Infections 2006;82(5):359-63.R12XP09/3; 
Sevelius JM, Reznick OG, Hart SL, Schwarcz S. Informing interventions: the importance of contextual factors in the prediction of sexual risk behaviors among transgender women. AIDS Education & Prevention 2009;21(2):113-27.R11XP09/3; 
Shah NG, Galai N, Celentano DD, Vlahov D, Strathdee SA. Longitudinal predictors of injection cessation and subsequent relapse among a cohort of injection drug users in Baltimore, MD, 1988-2000. Drug & Alcohol Dependence 2006;83(2):147-56.R10XP09/3; 
Shannon K, Bright V, Gibson K, Tyndall MW, Maka PP. Sexual and drug-related vulnerabilities for HIV infection among women engaged in survival sex work in Vancouver, Canada. Canadian Journal of Public Health 2007;Revue Canadienne de Sante Publique. 98(6):465-9.R12XP09/3; 
Sidat MM, Mijch AM, Lewin SR, Hoy JF, Hocking J, Fairley CK. Incidence of putative HIV superinfection and sexual practices among HIV-infected men who have sex with men. Sexual Health 2008;5(1):61-7.R14XP09/2; 
Spittal PM, Hogg RS, Li K, Craib KJ, Recsky M, Johnston C et al. Drastic elevations in mortality among female injection drug users in a Canadian setting. AIDS Care 2006;18(2):101-8.R10XP09/3; 
Spittal PM, Craib KJ, Teegee M, Baylis C, Christian WM, Moniruzzaman AK et al. The Cedar project: prevalence and correlates of HIV infection among young Aboriginal people who use drugs in two Canadian cities. International Journal of Circumpolar Health 2007;66(3):226-40.R04XP09/3; 
Templeton DJ, Mao L, Prestage G, Kaldor JM, Kippax S, Grulich AE. Demographic predictors of circumcision status in a community-based sample of homosexual men in Sydney, Australia. Sexual Health  2006;3(3):191-3.R01XP09/3; 
Thiede H, Jenkins RA, Carey JW, Hutcheson R, Thomas KK, Stall RD et al. Determinants of recent HIV infection among Seattle-area men who have sex with men. American Journal of Public Health 2009;99 Suppl 1:S157-S164.R01XP09/3; 
Thorne C, Newell ML. Injecting drug use in pregnant HIV-infected women in Europe. Medycyna Wieku Rozwojowego 2006;10(4):1005-16.R02XP09/3; R10XP09/3;
Thorne C. The mother-to-child HIV transmission epidemic in Europe: Evolving in the East and established in the West. AIDS 2006;20(10):1419-27.R02XP09/3; 
Tian LH, Peterman TA, Tao G, Brooks LC, Metcalf C, Malotte CK et al. Heterosexual anal sex activity in the year after an STD clinic visit.[see comment]. Sexually Transmitted Diseases 2008;35(11):905-9.R09XP09/1; 
Tillerson K. Explaining racial disparities in HIV/AIDS incidence among women in the U.S.: a systematic review. [Review] [24 refs]. Statistics in Medicine 2008;27(20):4132-43.R03XP09/3; R08XP09/3; R04XP09/3
Tyndall MW, Wood E, Zhang R, Lai C, Montaner JSG, Kerr T. HIV seroprevalence among participants at a Supervised Injection Facility in Vancouver, Canada: Implications for prevention, care and treatment. Harm Reduction Journal 2006;3, 2006. Article Number.R10XP09/3; 
Vaudrey J, Raymond HF, Chen S, Hecht J, Ahrens K, McFarland W. Indicators of use of methamphetamine and other substances among men who have sex with men, San Francisco, 2003-2006. Drug & Alcohol Dependence 2007;90(1):97-100.R01XP09/3; R10XP09/3;
Washington TA, Galai N, Cohn S, Celentano DD, Vlahov D, Strathdee SA. The relationship between self-reported sexual orientation and behavior in a sample of middle-aged male injection drug users. Archives of Sexual Behavior 2006;35(1):67-74.R10XP09/3; R01XP09/3;
Weir BW, Bard RS, O'Brien K, Casciato CJ, Stark MJ. Violence against women with HIV risk and recent criminal justice system involvement: prevalence, correlates, and recommendations for intervention. Violence Against Women 2008;14(8):944-60.R08XP09/3; R15XP09/3;
Werb D, Kerr T, Small W, Li K, Montaner J, Wood E. HIV risks associated with incarceration among injection drug users: implications for prison-based public health strategies. Journal of Public Health 2008;30(2):126-32.R10XP09/3; R13XP09/3;
Williams CC, Newman PA, Sakamoto I, Massaquoi NA. HIV prevention risks for Black women in Canada. Social Science and Medicine 2009;68(1):12-20.R03XP09/3; R08XP09/3;
Wood E, Montaner JS, Li K, Zhang R, Barney L, Strathdee SA et al. Burden of HIV infection among aboriginal injection drug users in Vancouver, British Columbia. American Journal of Public Health 2008;98(3):515-9.R10XP09/3; 
Wood E, Kerr T, Marshall BD, Li K, Zhang R, Hogg RS et al. Longitudinal community plasma HIV-1 RNA concentrations and incidence of HIV-1 among injecting drug users: prospective cohort study. BMJ 2009;338:b1649.R10XP09/3; 
Xia Q, Osmond DH, Tholandi M, Pollack LM, Zhou W, Ruiz JD et al. HIV prevalence and sexual risk behaviors among men who have sex with men: results from a statewide population-based survey in California. Journal of Acquired Immune Deficiency Syndromes: JAIDS 2006;41(2):238-45.R01XP09/3; 
Xia Q, Molitor F, Osmond DH, Tholandi M, Pollack LM, Ruiz JD et al. Knowledge of sexual partner's HIV serostatus and serosorting practices in a California population-based sample of men who have sex with men. AIDS 2006;20(16):2081-9.R01XP09/3;  QA - AGREE
Zablotska IB, Prestage G, Grulich AE, Imrie J. Differing trends in sexual risk behaviours in three Australian states: New South Wales, Victoria and Queensland, 1998-2006. Sexual Health 2008;5(2):125-30.R01XP09/3; 
Zenner D, Tomkins S, Charlett A, Wellings K, Ncube F. HIV prone occupational exposures: epidemiology and factors associated with initiation of post-exposure prophylaxis. Journal of Epidemiology and Community Health 2009;63(5):373-8.R16XP09/1; 
2 codes
Adimora AA, Schoenbach VJ, Martinson FEA, Coyne-Beasley T, Doherty I, Stancil TR et al. Heterosexually transmitted HIV infection among African Americans in North Carolina. Journal of Acquired Immune Deficiency Syndromes 2006;41(5):616-23.R03XP09/3; R09XP09/3;
Baillargeon JG, Paar DP, Wu H, Giordano TP, Murray O, Raimer BG et al. Psychiatric disorders, HIV infection and HIV/hepatitis co-infection in the correctional setting. AIDS Care 2008;20(1):124-9.R13XP09/3; R15XP09/3;
Brewer-Smyth K, Bucurescu G, Shults J, Metzger D, Sacktor N, van GW et al. Neurological function and HIV risk behaviors of female prison inmates. Journal of Neuroscience Nursing 2007;39(6):361-72.R13XP09/3; R08XP09/3;
Cedar PP, Pearce ME, Christian WM, Patterson K, Norris K, Moniruzzaman A et al. The Cedar Project: historical trauma, sexual abuse and HIV risk among young Aboriginal people who use injection and non-injection drugs in two Canadian cities. Social Science & Medicine 2008;66(11):2185-94.R10XP09/3; R04XP09/3;
Drummond PD, Mizan A, Wright B. HIV/AIDS knowledge and attitudes among West African immigrant women in Western Australia. Sexual Health 2008;5(3):251-9.R03XP09/3; R08XP09/3;
Edwards JM, Iritani BJ, Hallfors DD. Prevalence and correlates of exchanging sex for drugs or money among adolescents in the United States. Sexually Transmitted Infections 2006;82(5):354-8.R05XP09/3; R12XP09/3; QA - AGREE
El-Bassel N, Gilbert L, Wu E, Chang M, Gomes C, Vinocur D et al. Intimate partner violence prevalence and HIV risks among women receiving care in emergency departments: implications for IPV and HIV screening. Emergency Medicine Journal 2007;24(4):255-9.R15XP09/3; R08XP09/3;
Eyzaguirre L, Brouwer KC, Nadai Y, Patterson TL, Ramos R, Firestone CM et al. First molecular surveillance report of HIV type 1 in injecting drug users and female sex workers along the U.S.-Mexico border. AIDS Research & Human Retroviruses 2007;23(2):331-4.R15XP09/3; R10XP09/3;
Fisher HH, Eke AN, Cance JD, Hawkins SR, Lam WK. Correlates of HIV-related risk behaviors in African American adolescents from substance-using families: patterns of adolescent-level factors associated with sexual experience and substance use. Journal of Adolescent Health 2008;42(2):161-9.R03XP09/3; R05XP09/3; R15XP09/3
Gallagher KM, Sullivan PS, Lansky A, Onorato IM. Behavioral surveillance among people at risk for HIV infection in the U.S.: The national HIV behavioral surveillance system. Public Health Reports 2007;122(SUPPL. 1):32-8.R01XP09/1; R09XP09/1; R10XP09/1; COPIED FROM SR
Garofalo R, Mustanski BS, McKirnan DJ, Herrick A, Donenberg GR. Methamphetamine and young men who have sex with men: understanding patterns and correlates of use and the association with HIV-related sexual risk. Archives of Pediatrics & Adolescent Medicine 2007;161(6):591-6.R10XP09/3; R01XP09/3; R05XP09/3
Hallfors DD, Iritani BJ, Miller WC, Bauer DJ. Sexual and drug behavior patterns and HIV and STD racial disparities: the need for new directions. American Journal of Public Health 2007;97(1):125-32.R10XP09/3; R04XP09/3;
Hammett TM, Drachman-Jones A. HIV/AIDS, sexually transmitted diseases, and incarceration among women: National and southern perspectives. Sexually Transmitted Diseases 2006;33(7 SUPPL.):S17-S22.R03XP09/3; R08XP09/3; R13XP09/3
Hebert MR, Rose JS, Rosengard C, Clarke JG, Stein MD. Levels of trauma among women inmates with HIV risk and alcohol use disorders: behavioral and emotional impacts. Journal of Trauma & Dissociation 2007;8(2):27-46.R08XP09/3; R13XP09/3;
Hightow LB, Leone PA, Macdonald PD, McCoy SI, Sampson LA, Kaplan AH. Men who have sex with men and women: a unique risk group for HIV transmission on North Carolina College campuses.[see comment]. Sexually Transmitted Diseases 2006;33(10):585-93.R01XP09/3; R09XP09/3;
Lampinen TM, Mattheis K, Chan K, Hogg RS. Nitrite inhalant use among young gay and bisexual men in Vancouver during a period of increasing HIV incidence. BMC Public Health 2007;7:35.R10XP09/1; R01XP09/1; QA - MSM - code added 8.03.10
March JC, Oviedo-Joekes E, Romero M. Factors associated with reported hepatitis C and HIV among injecting drug users in ten European cities. Enfermedades Infecciosas y Microbiologia Clinica 2007;25(2):91-7.R10XP09/3; R13XP09/3;
Marks G, Millett GA, Bingham T, Bond L, Lauby J, Liau A et al. Understanding differences in HIV sexual transmission among Latino and black men who have sex with men: The Brothers y Hermanos Study. AIDS & Behavior 2009;13(4):682-90.R01XP09/3; R03XP09/3; R04XP09/3
Marshall BD, Wood E, Li K, Kerr T. Elevated syringe borrowing among men who have sex with men: a prospective study. Journal of Acquired Immune Deficiency Syndromes: JAIDS 2007;46(2):248-52.R10XP09/3; R01XP09/3;
Mehrabadi A, Craib KJP, Patterson K, Adam W, Moniruzzaman A, Ward-Burkitt B et al. The Cedar Project: A comparison of HIV-related vulnerabilities amongst young Aboriginal women surviving drug use and sex work in two Canadian cities. International Journal of Drug Policy 2008;19(2):159-68.R04XP09/3; R08XP09/3; R12XP09/3
Melendez RM, Exner TA, Ehrhardt AA, Dodge B, Remien RH, Rotheram-Borus MJ et al. Health and health care among male-to-female transgender persons who are HIV positive. American Journal of Public Health 2006;96(6):1034-7.R14XP09/2; R11XP09/2;
Miller M, Korves CT, Fernandez T. The social epidemiology of HIV transmission among African American women who use drugs and their social network members. AIDS Care 2007;19(7):858-65.R03XP09/3; R10XP09/3; R08XP09/3
Millett GA, Peterson JL, Wolitski RJ, Stall R. Greater risk for HIV infection of black men who have sex with men: a critical literature review. [Review] [148 refs]. American Journal of Public Health 2006;96(6):1007-19.R01XP09/3; R03XP09/3;
Mustanski B, Garofalo R, Herrick A, Donenberg G. Psychosocial health problems increase risk for HIV among urban young men who have sex with men: preliminary evidence of a syndemic in need of attention. Annals of Behavioral Medicine 2007;34(1):37-45.R01XP09/3; R05XP09/3;
Operario D, Soma T, Underhill K. Sex work and HIV status among transgender women: Systematic review and meta-analysis. Journal of Acquired Immune Deficiency Syndromes 2008;48(1):97-103.R11XP09/3; R12XP09/3;
Poulin C, Alary M, Lambert G, Godin G, Landry S, Gagnon H et al. Prevalence of HIV and hepatitis C virus infections among inmates of Quebec provincial prisons.[see comment]. CMAJ Canadian Medical Association Journal 2007;177(3):252-6.R13XP09/2; R10XP09/2; QA - Drug users - code added 08.03.10
Prestage G, Mao L, Jin F, Grulich A, Kaldor J, Kippax S. Sex work and risk behaviour among HIV-negative gay men. AIDS Care 2007;19(7):931-4.R01XP09/1; R12XP09/1; QA - AGREE
Prestage G, Fogarty AS, Rawstorne P, Grierson J, Zablotska I, Grulich A et al. Use of illicit drugs among gay men living with HIV in Sydney. AIDS 2007;21(SUPPL. 1):S49-S55.R01XP09/3; R10XP09/3;
Raj A, Reed E, Welles SL, Santana MC, Silverman JG. Intimate partner violence perpetration, risky sexual behavior, and STI/HIV diagnosis among heterosexual African American men. American Journal of Mens Health 2008;2(3):291-5.R03XP09/3; R09XP09/3;
Ravi A, Blankenship KM, Altice FL. The association between history of violence and HIV risk: a cross-sectional study of HIV-negative incarcerated women in Connecticut. Womens Health Issues 2007;17(4):210-6.R08XP09/1; R13XP09/1; R15XP09/1
Raymond HF, McFarland W. Racial mixing and HIV risk among men who have sex with men. AIDS & Behavior 2009;13(4):630-7.R01XP09/3; R03XP09/3; R04XP09/3; QA - AGREE
Romero EG, Teplin LA, McClelland GM, Abram KM, Welty LJ, Washburn JJ. A longitudinal study of the prevalence, development, and persistence of HIV/sexually transmitted infection risk behaviors in delinquent youth: implications for health care in the community. Pediatrics 2007;119(5):e1126-e1141.R10XP09/3; R05XP09/3; R13XP09/3
Saylors K, Daliparthy N. Violence against Native women in substance abuse treatment. American Indian & Alaska Native Mental Health Research (Online) 2006;13(1):32-51.R04XP09/3; R15XP09/3;
Thorne C, Newell ML. Injecting drug use in pregnant HIV-infected women in Europe. Medycyna Wieku Rozwojowego 2006;10(4):1005-16.R02XP09/3; R10XP09/3;
Tillerson K. Explaining racial disparities in HIV/AIDS incidence among women in the U.S.: a systematic review. [Review] [24 refs]. Statistics in Medicine 2008;27(20):4132-43.R03XP09/3; R08XP09/3; R04XP09/3
Vaudrey J, Raymond HF, Chen S, Hecht J, Ahrens K, McFarland W. Indicators of use of methamphetamine and other substances among men who have sex with men, San Francisco, 2003-2006. Drug & Alcohol Dependence 2007;90(1):97-100.R01XP09/3; R10XP09/3;
Washington TA, Galai N, Cohn S, Celentano DD, Vlahov D, Strathdee SA. The relationship between self-reported sexual orientation and behavior in a sample of middle-aged male injection drug users. Archives of Sexual Behavior 2006;35(1):67-74.R10XP09/3; R01XP09/3;
Weir BW, Bard RS, O'Brien K, Casciato CJ, Stark MJ. Violence against women with HIV risk and recent criminal justice system involvement: prevalence, correlates, and recommendations for intervention. Violence Against Women 2008;14(8):944-60.R08XP09/3; R15XP09/3;
Werb D, Kerr T, Small W, Li K, Montaner J, Wood E. HIV risks associated with incarceration among injection drug users: implications for prison-based public health strategies. Journal of Public Health 2008;30(2):126-32.R10XP09/3; R13XP09/3;
Williams CC, Newman PA, Sakamoto I, Massaquoi NA. HIV prevention risks for Black women in Canada. Social Science and Medicine 2009;68(1):12-20.R03XP09/3; R08XP09/3;
 3 codes
Fisher HH, Eke AN, Cance JD, Hawkins SR, Lam WK. Correlates of HIV-related risk behaviors in African American adolescents from substance-using families: patterns of adolescent-level factors associated with sexual experience and substance use. Journal of Adolescent Health 2008;42(2):161-9.R03XP09/3; R05XP09/3; R15XP09/3
Gallagher KM, Sullivan PS, Lansky A, Onorato IM. Behavioral surveillance among people at risk for HIV infection in the U.S.: The national HIV behavioral surveillance system. Public Health Reports 2007;122(SUPPL. 1):32-8.R01XP09/1; R09XP09/1; R10XP09/1; COPIED FROM SR
Garofalo R, Mustanski BS, McKirnan DJ, Herrick A, Donenberg GR. Methamphetamine and young men who have sex with men: understanding patterns and correlates of use and the association with HIV-related sexual risk. Archives of Pediatrics & Adolescent Medicine 2007;161(6):591-6.R10XP09/3; R01XP09/3; R05XP09/3
Hammett TM, Drachman-Jones A. HIV/AIDS, sexually transmitted diseases, and incarceration among women: National and southern perspectives. Sexually Transmitted Diseases 2006;33(7 SUPPL.):S17-S22.R03XP09/3; R08XP09/3; R13XP09/3
Marks G, Millett GA, Bingham T, Bond L, Lauby J, Liau A et al. Understanding differences in HIV sexual transmission among Latino and black men who have sex with men: The Brothers y Hermanos Study. AIDS & Behavior 2009;13(4):682-90.R01XP09/3; R03XP09/3; R04XP09/3
Mehrabadi A, Craib KJP, Patterson K, Adam W, Moniruzzaman A, Ward-Burkitt B et al. The Cedar Project: A comparison of HIV-related vulnerabilities amongst young Aboriginal women surviving drug use and sex work in two Canadian cities. International Journal of Drug Policy 2008;19(2):159-68.R04XP09/3; R08XP09/3; R12XP09/3
Miller M, Korves CT, Fernandez T. The social epidemiology of HIV transmission among African American women who use drugs and their social network members. AIDS Care 2007;19(7):858-65.R03XP09/3; R10XP09/3; R08XP09/3
Ravi A, Blankenship KM, Altice FL. The association between history of violence and HIV risk: a cross-sectional study of HIV-negative incarcerated women in Connecticut. Womens Health Issues 2007;17(4):210-6.R08XP09/1; R13XP09/1; R15XP09/1
Raymond HF, McFarland W. Racial mixing and HIV risk among men who have sex with men. AIDS & Behavior 2009;13(4):630-7.R01XP09/3; R03XP09/3; R04XP09/3; QA - AGREE
Romero EG, Teplin LA, McClelland GM, Abram KM, Welty LJ, Washburn JJ. A longitudinal study of the prevalence, development, and persistence of HIV/sexually transmitted infection risk behaviors in delinquent youth: implications for health care in the community. Pediatrics 2007;119(5):e1126-e1141.R10XP09/3; R05XP09/3; R13XP09/3
Tillerson K. Explaining racial disparities in HIV/AIDS incidence among women in the U.S.: a systematic review. [Review] [24 refs]. Statistics in Medicine 2008;27(20):4132-43.R03XP09/3; R08XP09/3; R04XP09/3

Social factors / Population level interventions (P10)
  1 code
Anderson M, Elam G, Gerver S, Solarin I, Fenton K. HIV/AIDS-related stigma and discrimination: Accounts of HIV-positive Caribbean people in the United Kingdom. Social Science and Medicine 2008;67(5):790-8.R04XP10/2; 
Cao D, Marsh JC, Shin HC. Gender and racial/ethnic disparities in the impact of HIV prevention programming in substance abuse treatment. American Journal of Drug & Alcohol Abuse 2008;34(6):730-40.R10XP10/3; 
ckson-Gomez J, Hilario H, Convey M, Corbett AM, Weeks M, Martinez M. The relationship between housing status and HIV risk among active drug users: a qualitative analysis. Substance Use & Misuse 2009;44(2):139-62.R10XP10/3; 
Corneil TA, Kuyper LM, Shoveller J, Hogg RS, Li K, Spittal PM et al. Unstable housing, associated risk behaviour, and increased risk for HIV infection among injection drug users. Health & Place 2006;12(1):79-85.R10XP10/3; 
Emlet CA. "You're awfully old to have this disease": experiences of stigma and ageism in adults 50 years and older living with HIV/AIDS. Gerontologist 2006;46(6):781-90.R06XP10/2; 
Falagas ME, Zarkadoulia EA, Pliatsika PA, Panos G. Socioeconomic status (SES) as a determinant of adherence to treatment in HIV infected patients: a systematic review of the literature. [Review] [38 refs]. Retrovirology 2008;5:13.R14XP10/2; 
Hallett TB, Garnett GP, Mupamberiyi Z, Gregson S. Measuring effectiveness in community randomized trials of HIV prevention. International Journal of Epidemiology 2008;37(1):77-87.R16XP10/1; R16XP13/1 - METHODOLOGY; R16XP02/1
Helion AM, Reddy DM, Kies AL, Morris DR, Wilson CM. Influence of communicator's race on efficacy of an HIV/STD prevention intervention among African American and Caucasian college students. Public Health Nursing 2008;25(5):440-50.R03XP10/1; 
Kerrigan D, Andrinopoulos K, Chung SE, Glass B, Ellen J. Gender ideologies, socioeconomic opportunities, and HIV/STI-related vulnerability among female, African-American adolescents. Journal of Urban Health 2008;85(5):717-26.R03XP10/3; R08XP10/3; R05XP10/3
Kidder DP, Wolitski RJ, Royal S, Aidala A, Courtenay-Quirk C, Holtgrave DR et al. Access to housing as a structural intervention for homeless and unstably housed people living with HIV: rationale, methods, and implementation of the housing and health study. AIDS & Behavior 2007;11(6 Suppl):149-61.R13XP10/2; 
Kinsler JJ, Wong MD, Sayles JN, Davis C, Cunningham WE. The effect of perceived stigma from a health care provider on access to care among a low-income HIV-positive population. AIDS Patient Care & Stds 2007;21(8):584-92.R14XP10/2; 
Latkin CA, Curry AD, Hua W, Davey MA. Direct and Indirect Associations of Neighborhood Disorder With Drug Use and High-Risk Sexual Partners. American Journal of Preventive Medicine 2007;32(6 SUPPL.):S234-S241.R16XP10/3; 
Maas B, Fairbairn N, Kerr T, Li K, Montaner JS, Wood E. Neighborhood and HIV infection among IDU: place of residence independently predicts HIV infection among a cohort of injection drug users. Health & Place 2007;13(2):432-9.R10XP10/3; 
Mahajan AP, Sayles JN, Patel VA, Remien RH, Sawires SR, Ortiz DJ et al. Stigma in the HIV/AIDS epidemic: a review of the literature and recommendations for the way forward. [Review] [89 refs]. AIDS 2008;22 Suppl 2:S67-S79.R16XP10/3; 
Marshall BD, Kerr T, Shoveller JA, Montaner JS, Wood E. Structural factors associated with an increased risk of HIV and sexually transmitted infection transmission among street-involved youth. BMC Public Health 2009;9:7.R05XP10/3; R15XP10/3;
Mimiaga MJ, Skeer M, Mayer KH, Safren SA. Study participation as a social group influencing sexual behaviours in an HIV-prevention trial for men who have sex with men. AIDS Care 2008;20(3):346-55.R01XP10/1; 
Prado G, Pantin H, Briones E, Schwartz SJ, Feaster D, Huang S et al. A randomized controlled trial of a parent-centered intervention in preventing substance use and HIV risk behaviors in Hispanic adolescents. Journal of Consulting & Clinical Psychology 2007;75(6):914-26.R05XP10/1; R04XP10/1;
Sandelowski M, Barroso J, Voils CI. Gender, race/ethnicity, and social class in research reports on stigma in HIV-positive women. [Review] [32 refs]. Health Care for Women International 2009;30(4):273-88.R14XP10/3; 
Warren JC, Fernandez MI, Harper GW, Hidalgo MA, Jamil OB, Torres RS. Predictors of unprotected sex among young sexually active African American, Hispanic, and White MSM: the importance of ethnicity and culture. AIDS & Behavior 2008;12(3):459-68.R01XP10/3; R05XP10/3; R04XP10/3
2 codes
Kerrigan D, Andrinopoulos K, Chung SE, Glass B, Ellen J. Gender ideologies, socioeconomic opportunities, and HIV/STI-related vulnerability among female, African-American adolescents. Journal of Urban Health 2008;85(5):717-26.R03XP10/3; R08XP10/3; R05XP10/3
Marshall BD, Kerr T, Shoveller JA, Montaner JS, Wood E. Structural factors associated with an increased risk of HIV and sexually transmitted infection transmission among street-involved youth. BMC Public Health 2009;9:7.R05XP10/3; R15XP10/3;
Prado G, Pantin H, Briones E, Schwartz SJ, Feaster D, Huang S et al. A randomized controlled trial of a parent-centered intervention in preventing substance use and HIV risk behaviors in Hispanic adolescents. Journal of Consulting & Clinical Psychology 2007;75(6):914-26.R05XP10/1; R04XP10/1;
Warren JC, Fernandez MI, Harper GW, Hidalgo MA, Jamil OB, Torres RS. Predictors of unprotected sex among young sexually active African American, Hispanic, and White MSM: the importance of ethnicity and culture. AIDS & Behavior 2008;12(3):459-68.R01XP10/3; R05XP10/3; R04XP10/3
3 codes
Kerrigan D, Andrinopoulos K, Chung SE, Glass B, Ellen J. Gender ideologies, socioeconomic opportunities, and HIV/STI-related vulnerability among female, African-American adolescents. Journal of Urban Health 2008;85(5):717-26.R03XP10/3; R08XP10/3; R05XP10/3
Warren JC, Fernandez MI, Harper GW, Hidalgo MA, Jamil OB, Torres RS. Predictors of unprotected sex among young sexually active African American, Hispanic, and White MSM: the importance of ethnicity and culture. AIDS & Behavior 2008;12(3):459-68.R01XP10/3; R05XP10/3; R04XP10/3

Combination prevention packages (P11)
1 code
Albarracin J, Albarracin D, Durantini M. Effects of HIV-prevention interventions for samples with higher and lower percents of Latinos and Latin Americans: a meta-analysis of change in condom use and knowledge. [Review] [39 refs]. AIDS & Behavior 2008;12(4):521-43.R04XP11/3; 
Copenhaver MM, Johnson BT, Lee IC, Harman JJ, Carey MP, SHARP Research Team. Behavioral HIV risk reduction among people who inject drugs: meta-analytic evidence of efficacy. Journal of Substance Abuse Treatment 2006;31(2):163-71.R10XP11/3;  QA - AGREE
Herbst JH, Beeker C, Mathew A, McNally T, Passin WF, Kay LS et al. The effectiveness of individual-, group-, and community-level HIV behavioral risk-reduction interventions for adult men who have sex with men: a systematic review.[see comment]. [Review] [165 refs]. American Journal of Preventive Medicine 2007;32(4 Suppl):S38-S67.R01XP11/3;  QA - AGREE
Perrino T, Fernandez MI, Bowen GS, Arheart K. Main partner's resistance to condoms and HIV protection among disadvantaged, minority women. Women and Health 42(3)()(pp 37-56), 2006 Date of Publication: 05 Apr 2006 2006;(3):37-56.R08XP11/3;  COPIED FROM RCT
Ritter A, Cameron J. A review of the efficacy and effectiveness of harm reduction strategies for alcohol, tobacco and illicit drugs. [Review] [193 refs]. Drug & Alcohol Review 2006;25(6):611-24.R10XP11/3; 
Sales JM, Milhausen RR, DiClemente RJ. A decade in review: building on the experiences of past adolescent STI/HIV interventions to optimise future prevention efforts. [Review] [65 refs]. Sexually Transmitted Infections 2006;82(6):431-6.R05XP11/3; 
Slesnick N, Kang MJ. The impact of an integrated treatment on HIV risk behavior among homeless youth: a randomized controlled trial. Journal of Behavioral Medicine 2008;31(1):45-59.R05XP11/1; R13XP11/1;
Underhill K, Operario D, Montgomery P. Systematic review of abstinence-plus HIV prevention programs in high-income countries. [Review] [157 refs]. PLoS Medicine / Public Library of Science 2007;4(9):e275.R16XP11/1; 
Volmink JA, Marais BJ. HIV: mother-to-child transmission. Clinical Evidence 2008;2008, 2008.R02XP11/3; 
2 codes
Slesnick N, Kang MJ. The impact of an integrated treatment on HIV risk behavior among homeless youth: a randomized controlled trial. Journal of Behavioral Medicine 2008;31(1):45-59.R05XP11/1; R13XP11/1;

International adaptation research (P12)
1 code
Bonell C, Strange V, Allen E, Barnett Page E. HIV prevention outreach in commercial gay venues in large cities: evaluation findings from London. Health Education Research 2006;21(4):452-64.R01XP12/3;  QA - AGREE
Morrison DM, Hoppe MJ, Gillmore MR, Kluver C, Higa D, Wells EA. Replicating an intervention: the tension between fidelity and adaptation. AIDS Education & Prevention 2009;21(2):128-40.R05XP12/1; 
Zometa CS, Dedrick R, Knox MD, Westhoff W, Siri RS, Debaldo A. Translation, cross-cultural adaptation and validation of an HIV/AIDS knowledge and attitudinal instrument. AIDS Education & Prevention 2007;19(3):231-44.R05XP12/3;  COPIED FROM SR

Other (P13)
1 code
Brown T, Grassly NC, Garnett G, Stanecki K. Improving projections at the country level: the UNAIDS Estimation and Projection Package 2005. [Review] [10 refs]. Sexually Transmitted Infections 2006;82 Suppl 3:iii34-iii40.R16XP13/3;  COPIED FROM SR
Bull SS, Vallejos D, Levine D, Ortiz C. Improving recruitment and retention for an online randomized controlled trial: experience from the Youthnet study. AIDS Care 2008;20(8):887-93.R05XP13/1; 
Caceres CF, Celentano DD, Coates TJ, Hartwell TD, Kasprzyk D, Kelly JA et al. Challenges and processes of selecting outcome measures for the NIMH collaborative HIV/STD prevention trial. AIDS 21(SUPPL 2)()(pp S29-S36), 2007 Date of Publication: Apr 2007 2007;(SUPPL. 2):S29-S36.R16XP13/3 - METHODOLOGY; 
Campbell JC, Baty ML, Ghandour RM, Stockman JK, Francisco L, Wagman J. The intersection of intimate partner violence against women and HIV/AIDS: a review. [Review] [104 refs]. International Journal of Injury Control & Safety Promotion 2008;15(4):221-31.R08XP13/3 - RESEARCH GAP IDENTIFICATION; R15XP13/3;
Chesson HW, White PJ. Influence of epidemic phase on the cost effectiveness of a prevention intervention for sexually transmitted infection: an exploratory analysis (DARE structured abstract). Sexually Transmitted Infections 2007;83:i25-i29.R16XP13/3 - COST EFFECTIVENESS;  QA - WAS PROTOCOL ONLY - UPDATED 20.01.10
Cohen DA, Wu S, Farley TA. Structural interventions to prevent HIV/sexually transmitted disease: are they cost-effective for women in the Southern United States? (DARE structured abstract). Sexually Transmitted Diseases 2006;33:S46-S49.R08XP13/1 - COST EFFECTIVENESS; 
Freedberg KA, Hirschhorn LR, Schackman BR, Wolf LL, Martin LA, Weinstein MC et al. Cost-effectiveness of an intervention to improve adherence to antiretroviral therapy in HIV-infected patients (DARE structured abstract). Journal of Acquired Immune Deficiency Syndromes 2006;43:S113-S118.R14XP13/2 - COST EFFECTIVENESS; 
Garfein RS, Swartzendruber A, Ouellet LJ, Kapadia F, Hudson SM, Thiede H et al. Methods to recruit and retain a cohort of young-adult injection drug users for the Third Collaborative Injection Drug Users Study/Drug Users Intervention Trial (CIDUS III/DUIT). Drug & Alcohol Dependence 2007;91 Suppl 1:S4-17.R10XP13/1; 
Guy R, Goller J, Leslie D, Thorpe R, Grierson J. No increase in HIV or sexually transmissible infection testing following a social marketing campaign among men who have sex with men. Journal of Epidemiology and Community Health 2009;63(5):391-6.R01XP13/3 - Evaluation of intervention; 
Heeren GA, Jemmott JB, III, Mandeya A, Tyler JC. Theory-based predictors of condom use among university students in the United States and South Africa. AIDS Education & Prevention 2007;19(1):1-12.R05XP13/3 - METHODOLOGY; 
Hoare A, Wilson DP, Regan DG, Kaldor J, Law MG. Using mathematical modelling to help explain the differential increase in HIV incidence in New South Wales, Victoria and Queensland: Importance of other sexually transmissible infections. Sexual Health 2008;5(2):169-87.R14XP13/3 - METHODOLOGY;  QA - Disagree - but correct - QUERY - add R1? no - R14 trumps
Hornberger J, Holodniy M, Robertus K, Winnike M, Gibson E, Verhulst E. A systematic review of cost-utility analyses in HIV/AIDS: implications for public policy. [Review] [128 refs]. Medical Decision Making 2007;27(6):789-821.R16XP13/3 - COST EFFECTIVENESS; R16XP03/3 - POLICY;
Hughes JP, Kulich M. Cluster randomized trials for HIV prevention. Current Opinion in HIV & AIDS 2006;1(6):471-5.R16XP13/3 - METHODOLOGY; 
Ingram BL, Flannery D, Elkavich A, Rotheram-Borus MJ. Common processes in evidence-based adolescent HIV prevention programs. AIDS & Behavior 2008;12(3):374-83.R05XP13/3 - ELEMENTS OF DESIGN; 
Link MW, Battaglia MP, Frankel MR, Osborn L, Mokdad AH. Address-based versus random-digit-dial surveys: comparison of key health and risk indicators. American Journal of Epidemiology 2006;164(10):1019-25.R16XP13/3; 
Lyles CM, Crepaz N, Herbst JH, Kay LS, HIV/AIDS Prevention Research Synthesis Team. Evidence-based HIV behavioral prevention from the perspective of the CDC's HIV/AIDS Prevention Research Synthesis Team.  AIDS Education & Prevention 2006;18(4 Suppl A):21-31.R16XP13/3  METHODOLOGY; 
Maisto SA, Conigliaro JC, Gordon AJ, McGinnis KA, Justice AC. An experimental study of the agreement of self-administration and telephone administration of the Timeline Followback interview. Journal of Studies on Alcohol & Drugs 2008;69(3):468-71.R10XP13/3; 
Malekinejad M, Johnston LG, Kendall C, Kerr LR, Rifkin MR, Rutherford GW. Using respondent-driven sampling methodology for HIV biological and behavioral surveillance in international settings: a systematic review. [Review] [40 refs]. AIDS & Behavior 2008;12(4 Suppl):S105-S130.R16XP13/3 - METHODOLOGY; 
McAuliffe TL, DiFranceisco W, Reed BR. Effects of question format and collection mode on the accuracy of retrospective surveys of health risk behavior: a comparison with daily sexual activity diaries. Health Psychology 2007;26(1):60-7.R16XP13/3 - METHODOLOGY; 
Mills E, Cooper C, Wu P, Rachlis B, Singh S, Guyatt GH. Randomized trials stopped early for harm in HIV/AIDS: A systematic survey. HIV Clinical Trials 7(1)()(pp 24-33), 2006 Date of Publication: Jan 2006 2006;(1):24-33.R14XP13/2 - METHODOLOGY; 
Mizuno Y, Purcell DW, Mackenzie S, Tobin KE, Wunch T, Arnsten JH et al. Acceptability of A-CASI by HIV-positive IDUs in a multisite, randomized, controlled trial of behavioral intervention (INSPIRE). Journal of Acquired Immune Deficiency Syndromes: JAIDS 2007;46 Suppl 2:S48-S54.R14XP13/2; 
Newman PA, Duan N, Lee SJ, Rudy E, Seiden D, Kakinami L et al. Willingness to participate in HIV vaccine trials: the impact of trial attributes. Preventive Medicine 2007;44(6):554-7.R15XP13/1 - METHODOLOGY;  COPIED FROM SR
NIMH Collaborative HIV/STD Prevention Trial Group. Challenges and processes of selecting outcome measures for the NIMH Collaborative HIV/STD Prevention Trial. AIDS 2007;21 Suppl 2:S29-S36.R16XP13/3 - METHODOLOGY; 
NIMH Multisite HIV/STD Prevention Trial for African American Couples Group. Measure of HIV/STD risk-reduction: strategies for enhancing the utility of behavioral and biological outcome measures for African American couples. Journal of acquired immune deficiency syndromes (1999) 2008;49 Suppl 1:S35-S41.R14XP13/3 - METHODOLOGY;  COPIED FROM RCT
NIMH Multisite HIV/STD Prevention Trial for African American Couples Group. Methodological overview of an African American couple-based HIV/STD prevention trial. Journal of Acquired Immune Deficiency Syndromes: JAIDS 2008;49 Suppl 1:S3-14.R03XP13/3; 
Noguchi K, Albarracin D, Durantini MR, Glasman LR. Who participates in which health promotion programs? A meta-analysis of motivations underlying enrollment and retention in HIV-prevention interventions.  Psychological Bulletin 2007;133(6):955-75.R16XP13/3 - METHODOLOGY; 
Pals SL, Beaty BL, Posner SF, Bull SS. Estimates of intraclass correlation for variables related to behavioral HIV/STD prevention in a predominantly African American and Hispanic sample of young women. Health Education & Behavior 2009;36(1):182-94.R03XP13/3; R04XP13/3; R05XP13/3; R08XP13/3 - QA - AGREE BM
Pinto RM, McKay MM, Bapiste D, Bell CC, Madison Boyd S. Motivators and barriers to participation of ethnic minority families in a family-based HIV prevention program. Social Work in Mental Health 2007;5(112):187-201.R04XP13/3 - METHODOLOGY; 
Prado G, Pantin H, Schwartz SJ, Lupei NS, Szapocznik J. Predictors of engagement and retention into a parent-centered, ecodevelopmental HIV preventive intervention for Hispanic adolescents and their families. Journal of Pediatric Psychology 2006;31(9):874-90.R04XP13/3 - METHODOLOGY; R05XP13/3; COPIED FROM RCT
Sales JM, Spitalnick J, Milhausen RR, Wingood GM, DiClemente RJ. Validation of the worry about sexual outcomes scale for use in STI/HIV prevention interventions for adolescent females. Health Education Research 2009;24(1):140-52.R05XP13/3 - METHODOLOGY; R08XP13/3;
Sandelowski M, Voils CI, Barroso J, Lee EJ. "Distorted into clarity": a methodological case study illustrating the paradox of systematic review. [Review] [72 refs]. Research in Nursing & Health  2008;31(5):454-65.R14XP13/2 - METHOLOGY; 
Schwarcz S, Spindler H, Scheer S, Valleroy L, Lansky A. Assessing representativeness of sampling methods for reaching men who have sex with men: a direct comparison of results obtained from convenience and probability samples. AIDS & Behavior 2007;11(4):596-602.R01XP13/3 - METHODOLOGY; 
Townsend CL, Cortina-Borja M, Peckham CS, de RA, Lyall H, Tookey PA. Low rates of mother-to-child transmission of HIV following effective pregnancy interventions in the United Kingdom and Ireland, 2000-2006. AIDS 2008;22(8):973-81.R02XP13/3 - EFFICACY OF INTERVENTIONS;  QA - AGREE
Villarruel AM, Jemmott LS, Jemmott JB, Eakin BL. Recruitment and retention of Latino adolescents to a research study: lessons learned from a randomized clinical trial. J Spec Pediatr Nurs 2006;11(4):244-50.R04XP13/3; R05XP13/3;
Wang L, Li Y, Peng L, Wen J, Sun X. The quality of reporting of systematic reviews or meta-analyses on peripartum antiretroviral therapies for preventing the risk of mother-to-child transmission of HIV [abstract]. XV Cochrane Colloquium; 2007 Oct 23 27; Sao Paulo, Brazil 2007;146.R02XP13/1 - METHODOLOGY; 
Wong WCW, Cheung CSK, Hart GJ. Development of a quality assessment tool for systematic reviews of observational studies (QATSO) of HIV prevalence in men having sex with men and associated risk behaviours. Emerging Themes in Epidemiology 5, 2008 Article Number: 23 Date of Publication: 2008 2008.R01XP13/3 - METHODOLOGY; 
Woods ER, Klein JD, Wingood GM, Rose ES, Wypij D, Harris SK et al. Development of a new Adolescent Patient-Provider Interaction Scale (APPIS) for youth at risk for STDs/HIV. The Journal of adolescent health : official publication of the Society for Adolescent Medicine 2006;38(6):753.R05XP13/3 - METHODOLOGY; 
Zaric GS, Bayoumi AM, Brandeau ML, Owens DK. The cost-effectiveness of counseling strategies to improve adherence to highly active antiretroviral therapy among men who have sex with men (DARE structured abstract). Medical Decision Making 2008;28:359-76.R14XP13/2 - COST EFFECTIVENESS;  QA - WAS PROTOCOL ONLY - UPDATED 20.01.10
Zimmerman RS, Noar SM, Feist-Price S, Dekthar O, Cupp PK, Anderman E et al. Longitudinal test of a multiple domain model of adolescent condom use. Journal of Sex Research 2007;44(4):380-94.R05XP13/3 - METHODOLOGY; 
2 codes
Barham L, Lewis D, Latimer N. One to one interventions to reduce sexually transmitted infections and under the age of 18 conceptions: A systematic review of the economic evaluations. Sexually Transmitted Infections 83(6)()(pp 441-447), 2007 Date of Publication: Oct 2007 2007;(6):441-7.R05XP04/3; R05XP13/3 - ECONOMIC EVALUATION;
Campbell JC, Baty ML, Ghandour RM, Stockman JK, Francisco L, Wagman J. The intersection of intimate partner violence against women and HIV/AIDS: a review. [Review] [104 refs]. International Journal of Injury Control & Safety Promotion 2008;15(4):221-31.R08XP13/3 - RESEARCH GAP IDENTIFICATION; R15XP13/3;
Hallett TB, Garnett GP, Mupamberiyi Z, Gregson S. Measuring effectiveness in community randomized trials of HIV prevention. International Journal of Epidemiology 2008;37(1):77-87.R16XP10/1; R16XP13/1 - METHODOLOGY; R16XP02/1
Lee LM, McKenna MT. Monitoring the incidence of HIV infection in the United States. Public Health Reports 2007;122(SUPPL. 1):72-9.R16XP09/3; R16XP13/3; QA - QUERY - is this prevention or measuring prevention? does this matter?! COPIED FROM SR - Code added METHODOLOGY 08.03.10
Pals SL, Beaty BL, Posner SF, Bull SS. Estimates of intraclass correlation for variables related to behavioral HIV/STD prevention in a predominantly African American and Hispanic sample of young women. Health Education & Behavior 2009;36(1):182-94.R03XP13/3; R04XP13/3; R05XP13/3; R08XP13/3 - QA - AGREE BM
Prado G, Pantin H, Schwartz SJ, Lupei NS, Szapocznik J. Predictors of engagement and retention into a parent-centered, ecodevelopmental HIV preventive intervention for Hispanic adolescents and their families. Journal of Pediatric Psychology 2006;31(9):874-90.R04XP13/3 - METHODOLOGY; R05XP13/3; COPIED FROM RCT
Sales JM, Spitalnick J, Milhausen RR, Wingood GM, DiClemente RJ. Validation of the worry about sexual outcomes scale for use in STI/HIV prevention interventions for adolescent females. Health Education Research 2009;24(1):140-52.R05XP13/3 - METHODOLOGY; R08XP13/3;
Vidanapathirana J, Randeniya M. Publication of responses to a Cochrane systematic review on mass media interventions for HIV testing [abstract]. XV Cochrane Colloquium; 2007 Oct 23 27; Sao Paulo, Brazil 2007;149-50.R16XP05/1; R16XP13/1 - DISSEMINATION;
Villarruel AM, Jemmott LS, Jemmott JB, Eakin BL. Recruitment and retention of Latino adolescents to a research study: lessons learned from a randomized clinical trial. J Spec Pediatr Nurs 2006;11(4):244-50.R04XP13/3; R05XP13/3;
3 codes
Pals SL, Beaty BL, Posner SF, Bull SS. Estimates of intraclass correlation for variables related to behavioral HIV/STD prevention in a predominantly African American and Hispanic sample of young women. Health Education & Behavior 2009;36(1):182-94.R03XP13/3; R04XP13/3; R05XP13/3; R08XP13/3 - QA - AGREE BM


 
